# A Global-Scale Time Series Dataset for Groundwater Studies within the Earth System

**DOI:** 10.1038/s41597-026-06966-1

**Published:** 2026-03-09

**Authors:** Annemarie Bäthge, Claudia Ruz Vargas, Gunnar Lischeid, Raoul Collenteur, Mark Cuthbert, Jan Fleckenstein, Martina Flörke, Inge de Graaf, Sebastian Gnann, Andreas Hartmann, Xander Huggins, Nils Moosdorf, Yoshihide Wada, Thorsten Wagener, Robert Reinecke

**Affiliations:** 1 Institute of Geography, Johannes Gutenberg-University Mainz, Mainz, Germany; 2 International Groundwater Resources Assessment Centre (IGRAC), Delft, The Netherlands; 3 Research Area 4: ‘Simulation & Data Science’, Leibniz Centre for Agricultural Landscape Research, Müncheberg, Germany; 4 Institute of Environmental Science and Geography, University of Potsdam, Potsdam, Germany; 5 Department Water Resources and Drinking Water, Eawag, Dübendorf, Switzerland; 6 School of Earth and Environmental Sciences, Cardiff University, Cardiff, UK; 7 Department of Hydrogeology, Helmholtz Center for Environmental Research - UFZ, Leipzig, Germany; 8 Institute of Engineering Hydrology and Water Resources Management, Ruhr University Bochum, Bochum, Germany; 9 Earth Systems and Global Change Group, Wageningen University and Research, Wageningen, the Netherlands; 10 Chair of Hydrology, University of Freiburg, Freiburg, Germany; 11 Institute of Groundwater Management, Technical University of Dresden, Dresden, Germany; 12 Institute for Resources, Environment, and Sustainability, University of British Columbia, Vancouver, BC Canada; 13 High Meadows Environmental Institute, Princeton University, Princeton, NJ USA; 14 Stockholm Resilience Centre, Stockholm University, Stockholm, Sweden; 15 Leibniz Centre for Tropical Marine Research (ZMT), Bremen, Germany; 16 Institute of Geosciences, Kiel University, Kiel, Germany; 17 Biological and Environmental Science and Engineering Division, King Abdullah University of Science and Technology, Thuwal, Saudi Arabia

**Keywords:** Hydrology, Environmental sciences

## Abstract

Groundwater is a central component of the Earth system. However, our understanding of how it is dynamically interlinked with the atmosphere, hydrosphere, cryosphere, biosphere, geosphere, and anthroposphere remains limited. In the pursuit of understanding groundwater dynamics across diverse global settings, we present GROW (the global-scale integrated GROundWater package). This analysis-ready, quality-controlled dataset combines depth to groundwater and level time series from 55 countries, 91% from North America, India, Europe, and Australia, with associated Earth system variables. The dataset contains >200,000 time series with either daily, monthly, or yearly temporal resolution, accompanied by 36 time series or static attributes of meteorological, hydrological, geophysical, vegetation, and anthropogenic variables (e.g., precipitation, drainage density, rock type, NDVI, land use). 34 data flags regarding well features (e.g., coordinates and country), as well as time series characteristics (e.g., gap fraction or autocorrelation), facilitate quick data filtering. GROW provides a foundation for understanding large-scale groundwater processes in space and time, as well as for calibrating and evaluating models that simulate groundwater dynamics within the Earth system.

## Background & Summary

Access to groundwater is pivotal for humans and ecosystems that depend on this freshwater source^1–3^. Groundwater is the largest store of unfrozen freshwater on Earth, accounting for approximately 99% of the Earth’s accessible freshwater^3^. It is estimated to contribute between 19% (PCR-GLOBWB, period 2000–2015^4^) and 22% (WaterGAP v2.2e, period 1991–2019^5^) of total global water withdrawal. Beyond its role as a source for anthropogenic water consumption, groundwater plays a key role for biodiversity. It sustains various ecosystems, especially phreatophytic vegetation in drylands, surface waters, and wetlands^6^. Saccò *et al*.^2^ estimated that 75% of habitable land area is ecologically interconnected with groundwater. Ecosystems that rely on groundwater, also called groundwater-dependent ecosystems^7^, provide multiple benefits. They regulate climate, floods, water quality, host biodiversity and have recreational and cultural value^2,8^.

While critical to humans and ecosystems, groundwater accessibility is threatened by human-driven changes in hydrological storages and fluxes. Among others, groundwater accessibility is limited by the depth of the water table. When water tables decline, groundwater may become inaccessible for anthropogenic water supply^9^ and groundwater-dependent ecosystems^6^. Rohde *et al*.^6^ showed that 53% of groundwater-dependent ecosystems in drylands are at risk from declining regional water tables. One of the biggest threats is over-abstraction, which has led to widespread groundwater depletion^3,10,11^. Based on estimations for 2010, 70% of global groundwater abstraction was attributed to irrigation^1^. The pressure that irrigation exerts on groundwater accessibility was highlighted by Jasechko *et al*.^12^. They^12^ found that aquifer systems with water tables in rapid decline are most common in agricultural drylands. Consequently, it is not surprising that the water scarcity hotspots in the world occur in arid and semiarid regions where groundwater withdrawal for irrigation is prevalent (i.e., Pakistan, northeastern China, and the Middle East)^11,13^. Humans further impact water tables through land use changes, e.g., urbanization, wetland loss, and conversion to agricultural land^11,14,15^. This, in turn, changes infiltration^16^, interception, evapotranspiration^17^, runoff^14^ and recharge^11,17^. Additionally, climate change and its consequences alter the water cycle in manifold ways (e.g., soil moisture depletion^18^, sea level rise^18,19^, melting of glaciers, and thawing of permafrost^17^). However, the quantification of these impacts on groundwater systems, and their variation in space and time, remain underevaluated^3,20–22^.

To better understand how these changes may affect water table dynamics, we must investigate groundwater as an integral part of the Earth system^23–26^. Studying groundwater not in isolation, but together with environmental variables within the Earth system (here called Earth system variables), reveals their interactions with and influence on groundwater. In this way, controls behind water table dynamics can be addressed and their respective importance compared^24^. Groundwater’s embeddedness within the Earth system suggests that its dynamics may be shaped more by the configuration of multiple Earth system variables across these interconnected systems than by individual system properties^27^. We understand that the Earth system is not only composed of natural components like the atmosphere, geosphere, hydrosphere, cryosphere, and biosphere^28^ but also includes the anthroposphere^14^.

In Table 1, we present relationships of groundwater within different Earth system components that have been identified as crucial for quantitative groundwater dynamics. The selection of the Earth system variables is rooted in existing literature and limited due to data availability. To provide a clear overview, the Earth system variables are categorized into six components: Atmosphere (1), Geosphere (2), Hydrosphere (3), Cryosphere (4), Biosphere (5), and Anthroposphere (6).

**Table 1:** Overview of prominent relationships that highlight groundwater’s interconnectedness within the Earth system.

Earth system component + Earth system variables in GROW^56^	Relationship with groundwater
**1 Atmosphere** + Precipitation + Potential evapotranspiration + Actual evapotranspiration + Air temperature + Koeppen-Geiger classification + Hydrobelt	⦁ Precipitation controls the amount of atmospheric water that is available to replenish groundwater^30,53,99^. ⦁ In case of a bidirectional connection of atmosphere and groundwater, water is lost to the atmosphere by actual evapotranspiration^100,101^. ⦁ Precipitation and potential evapotranspiration together form temporal (seasons) and spatial (climate zones) gradients along which groundwater recharge patterns emerge^24,48,102,103^. ⦁ Air temperature dictates the capacity of ambient air to hold water vapor. With higher air temperature, the saturation vapor pressure increases, and more water is present in gaseous form. By that, potential evapotranspiration can increase with temperature^73^. Air temperature also affects processes such as vegetation growth and snow accumulation, which in turn impact groundwater processes (see Cryosphere, Biosphere.
**2 Geosphere** + Ground elevation + Topographic slope + Rock type + Permeability + Total porosity + Aquifer type + Saturated hydraulic conductivity for topsoil and subsoil + Soil texture class for topsoil and subsoil	⦁ Topography is a core driver of groundwater flow, where discharge areas develop within topographic lows^49,104^. ⦁ Topographic slope influences the partitioning of water into groundwater recharge. Steeper slopes can promote less diffuse recharge because water moves overland and downhill rather than percolating into the ground^30,101^. On the other hand, steeper slopes are also related to deep infiltration and deep groundwater flow paths^104,105^. ⦁ Groundwater is stored in and flows through pores and fractures of geological layers^106^. Geological properties like permeability and porosity strongly influence groundwater flow, response time^100,101,107^ and storage capacity^96^. ⦁ Soil characteristics like permeability, among others determined by soil texture, affect capillary rise and the percolation of water^30,101^. With larger sand fractions in soil, permeability and consequently recharge usually increase^101,108^.
**3 Hydrosphere** + Distance between perennial streams + Drainage density	⦁ Groundwater buffers surface water level fluctuations and may sustain streamflow during dry periods^11,17,109^. ⦁ Infiltration from surface waters is often the main source of groundwater recharge in drylands^53,109^.
**4 Cryosphere** + Permafrost cover + Glacier cover + Snow depth + (Average) days per year with snow cover	⦁ In cold regions, soil may be permanently frozen (permafrost), which inhibits groundwater recharge^110^, groundwater flow and groundwater-surface water connectivity^17^. ⦁ Where surface catchments are (partly) covered with glaciers, their meltwater can be an important source of recharge^111^. ⦁ In snowy climates, snow cover accumulates during cold periods and is released during melt, delaying the recharge response^23,99,112^.
**5 Biosphere** + Interception loss + NDVI + Leaf area index – low vegetation + Leaf area index – high vegetation + Groundwater-dependent ecosystems	⦁ Vegetation affects interception^101^, transpiration, and soil structure^30,101,113^, thereby determining the water fluxes to and from groundwater. It is generally accepted that high vegetation density is associated with reduced groundwater recharge^30,108^, but this can change under specific settings (e.g., dry tropical regions)^114^. ⦁ Groundwater supplies the soil root zone with water and can serve as a reliable water source in dry climates^49,115^. As a result, the water table can regulate the depth of plant roots and biomass^116^. ⦁ Where vegetation roots are directly abstracting from groundwater (phreatophytes), their health is sensitive to water table fluctuations^117^.
**6 Anthroposphere** + Total water withdrawal for industrial and domestic use + Land use fraction of rainfed cropland, irrigated cropland, pastures, forests & natural vegetation and urban areas + Main land use + Groundwaterscapes	⦁ Groundwater withdrawal can lead to short-term or persistent decline of water tables^9,109^. ⦁ Land use practices like surface water-fed irrigation, forest clearance or urbanization can influence groundwater recharge^101^.

For each Earth system component, the associated Earth system variables included in GROW^56^ are listed. More information about the Earth system variables is provided in Tables 4 & 5 and the dataset documentation on Zenodo: 10.5281/zenodo.15149480. Some Earth system variables can be related to multiple components but are only named in one. The listed literature on relationships with groundwater is exemplary and does not represent a systematic literature review spanning all processes within an Earth system component.

The general understanding of the complex interconnectedness of groundwater within the Earth system is still incomplete^17,20,29^. The knowledge gaps include a lack of observations in environments like high-latitudes^17,30^ and regions without agricultural influence^27^, a lack of knowledge regarding environmental characteristics like the spatial heterogeneity of the subsurface hydraulic conductivity^31,32^ and insufficient knowledge about measurement errors^33^. Groundwater modeling is constrained by the uncertainty regarding processes and state variables, particularly evident for input and boundary conditions. Other restricting factors in modelling are the oversimplification of processes and the lack of clarity regarding the optimal implementation^29,33,34^. These limitations contribute to highly uncertain groundwater simulations^17,34^.

*In situ* measurements are key to closing these knowledge gaps^17,23,35^, because they provide the closest approximation of ‘ground truth’. In combination with variables that are driving and influencing groundwater dynamics, new groundwater-related relationships can be uncovered^17,29,36^. This enhances our integrated process understanding of groundwater within the Earth system^36^ and, consequently, can lead to improved conceptual/perceptual models. Other than that, global models could highly benefit from global time-varying groundwater datasets for parametrization and model evaluation^34^. A dataset built for these purposes should preferably:

1. be a **global-scale dataset** that captures the natural variability of the included variables, which enables better intercomparison between regions^37^ and helps reveal large-scale processes and connections^3,11,23,38^. It can be used to develop, calibrate, and evaluate large-scale hydrological, land-surface, and climate models^29,35,39^;
2. contain **time series data** that provide insights into temporal patterns (e.g., seasonality), extreme events, regime shifts and cause-effect lags^3,14,34,36,40^;
3. combine groundwater observations with **associated variables of the Earth system** that can be used to explain groundwater dynamics^23,30,41^. Huggins *et al*.^36^ emphasized that there is an underrealized potential in combining existing groundwater and Earth system data to uncover relationships that have not yet been recognized;
4. include **metadata** to improve comparability and the assessment of data uncertainty according to best practices in large-sample datasets^20,29,37^;
5. be **standardized, harmonized** and **freely available** according to the FAIR principles to enhance applicability and accessibility^36,37,42^. The use of data is further incentivized by ‘analysis-ready’ data. A consistent temporal resolution, gap-filling and data characterizing flags significantly reduce preprocessing time for users.

A dataset that fulfills most of these aspects, but has a focus on streamflow data, is CARAVAN^39^. It combines national collections of measured daily streamflow time series with meteorological forcing data from ERA5-Land^43^ and catchment attributes from HydroAtlas^44^. *In situ* groundwater time series are not included in CARAVAN. There are datasets that have been developed to understand quantitative groundwater dynamics. Regional sets of groundwater level time series accompanied with additional environmental information exist, e.g., for Chile^26^, Switzerland^45^ or the Baltic countries^46^. But a comparison of these datasets without extensive preprocessing is difficult as they are standardized differently and contain different variables. At the global scale, static datasets such as Fan *et al*.^38^ with depth to groundwater and Moeck *et al*.^30^ with groundwater recharge provide steady-state groundwater records for many locations worldwide. The Global Groundwater Monitoring Network (GGMN)^47^ contains groundwater level or depth time series from different regions of the world, but lacks an extensive set of associated Earth system variables. Consequently, past studies that analyzed observational data have either focused on steady-state data^48,49^, which can only be used to a limited extent to understand temporal patterns, or have concentrated on regional^41,50–52^ to continental scales^53–55^. The results of regional to continental studies^52,55^ can help us to understand global-scale patterns like atmospheric-ocean oscillations (e.g., climate teleconnections such as El Niño–Southern Oscillation)^52^. A global-scale dataset can provide even further insights as teleconnections, global interlinkages^29^, and the diversity of environmental settings can be investigated with a consistent method. To our knowledge, Jasechko *et al*.^12^ were the first to analyze temporally dynamic groundwater level data on a global scale in relation to two influencing variables: Aridity index and land fraction under cultivation. They focused on time series with yearly resolution. This leaves potential for more comprehensive studies, integrating more timescales and a wider set of Earth system variables.

Here, we present GROW (the global-scale integrated GROundWater package)^56^, a quality-controlled dataset that accompanies groundwater depth and level time series from around the world with associated Earth system variables. GROW^56^ is designed to enable large-sample spatiotemporal groundwater analysis without further preprocessing, making it ‘analysis-ready.’ The groundwater data included in GROW^56^ have been sourced from the Global Groundwater Monitoring Network^47^ and Groundwater Observations Repository^57^ by the International Groundwater Resources Assessment Centre (IGRAC). The source data were temporally harmonized to either a daily, monthly, or yearly resolution to ensure equal time step intervals. Per time series, gap fraction and single gap lengths were constrained to stay below certain thresholds (10%). Remaining gaps were linearly filled. Records that indicate measurement errors were flagged. Duplicate time series and wells were discarded. Overall, GROW^56^ contains 204,292 time series. 85% of the time series have a yearly resolution, 9% have a monthly resolution and 6% have a daily resolution. 51% of the time series are at least 10 years long. Covering all major Earth system components, the groundwater data are accompanied by 36 groundwater-associated variables (time series and attributes) that are categorized into: atmosphere (n = 6), geosphere (10), hydrosphere (2), cryosphere (4), biosphere (5) and anthroposphere (9) (see also Table 1). 34 data flags related to well features (e.g., location coordinates, country), as well as time series characteristics (e.g., time series length, trend direction, autocorrelation, total gap fraction), enable targeted filtering.

With GROW^56^, we offer a dataset to the global community that can be used to understand spatiotemporal groundwater dynamics in the Earth system. Cumulative effects of multiple controls on groundwater time series can be studied in space and time. Processes conceptualized for specific environmental settings can be transferred to regions with no data but the same environmental conditions. GROW^56^ offers great potential for tools like machine learning^35,51,58,59^ and principal component analysis^60^, which can be used to uncover hidden connections and test conceptual assumptions in large datasets. In addition to this, the harmonized groundwater time series in GROW^56^ provide a ready-to-use calibration and validation dataset for global hydrological, land-surface, or climate modeling. We not only encourage hydrologists to use the dataset but also experts and scientists from other fields because groundwater dynamics feed back into other storages and fluxes of the Earth system. It should be noted that the dataset shows a spatial bias, with most wells located in North America (51% of the wells), India (17%), Europe (13%), and Australia (10%). Additional biases occur toward arid (26%) and temperate (58%) climates, low elevations (62% of the wells are located below 200 m altitude), and a higher anthropogenic impact (67% of the wells are located on anthropogenically used land). Furthermore, uncertainties from the utilized data products are inherited by the GROW dataset^56^. The limited global representativeness and data uncertainties have implications for analysis. They are discussed in the Technical Validation section. Guidance on addressing these limitations is provided in the Usage Notes section.

## Methods

### Groundwater time series

The *in situ* groundwater time series in GROW^56^ were derived from the global groundwater datasets Global Groundwater Monitoring Network (GGMN)^47^ and Groundwater Observations Repository^57^ hosted by the International Groundwater Resources Assessment Centre (IGRAC). For the GGMN dataset^47^, IGRAC collects and shares monitoring groundwater data like groundwater level, groundwater quality parameters, and groundwater-related metadata from national and subnational authorities. This data is regularly updated. In addition, IGRAC maintains the Groundwater Observations Repository^57^ that contains groundwater data from research studies and other sources, only added once. IGRAC standardizes the format of the data obtained from the provider and enriches it with descriptive data flags but does not further alter the groundwater records. These source datasets were used because they are currently the most comprehensive and publicly accessible collection of observed groundwater time series. Among others, groundwater time series from the study of Jasechko *et al*.^12^ are partially included in Groundwater Observations Repository^57^ and make up 86% of latter dataset. The two datasets together contain 251,396 time series (as of August 2025) and have heterogeneous temporal scales and reference point elevations. The groundwater records of a time series are either given as depth from the ground elevation to groundwater (86% in GROW^56^), depth from the top of the well to groundwater (<1%), or groundwater level elevation above mean sea level (14%). We do not label the groundwater data as hydraulic head because of the following reasons. The depth to groundwater records would need to be transferred to groundwater level elevation, but the ground elevation is only specified by the original data provider for 24% of the groundwater depth time series in GROW^56^. Other than that, the information on whether the aquifer is confined or unconfined is only provided for 6% of the time series in GROW^56^. An estimation is not possible with the available data. For readability, we use ‘water table’ as a term in the rest of the article when referring to both ‘depth to groundwater’ and ‘groundwater level’ records. This does not affect the published data, which contains groundwater information for both reference points (either depth to groundwater or groundwater level). The reference point per time series is given in the attributes table of the final dataset. For clarity, each reference point is assigned its own column in the groundwater time series table. In addition to the time series, the GGMN^47^ and Groundwater Observations^57^ provide static attributes for each well, including the reference point, coordinates, country, data license, confinement, and more.

The preprocessing of the time series data consists of eleven steps (Table 2, steps 1–11). In addition, attribute data were checked for duplicates and erroneous coordinates (Table 3, steps 12–14). The individual preprocessing steps are described below. Tables 2, 3 provide an overview of the number and percentage of discarded or flagged time series and the data flags created during preprocessing. The Supplements Tables S.1-1, S.1-4 describe the effects of preprocessing on time series characteristics. Examples of discarded or flagged time series are provided in the Supplements Figures S.1-1–S.1-5.

1. **Discarding time series with fewer than 2 records**
  Empty records and duplicates are removed. In this study, time series are defined as containing a minimum of two records at different timestamps. This criterion is checked at the start and after every preprocessing step in which data are aggregated or removed.
2. **Reconciling records to ensure that only one groundwater reference point elevation is provided per time series**
  Records with more than one reference point in the same time series exist. 3.8% of the time series contain both depth to groundwater and groundwater level information for every timestamp. To ensure consistency and comparability, depth to groundwater records are removed from these time series, because we prefer groundwater level records. They have the same reference height (elevation above mean sea level) and can be compared directly between different wells.
3. **Removing numeric placeholders for missing records**
  Because the entries ‘- 999’ and’- 9,999’ are common to mark missing records and are below realistic value ranges, they are removed from groundwater level time series. Ground elevation at the well location and top of the well elevation which is ‘-999’ or ‘-9,999’ is replaced with ‘NA’ (Not available). Both data flags are provided by the original data provider for 24% (ground elevation) and <1% (top of the well elevation) of the wells.
4. **Temporal aggregation of time series to either daily, monthly, or yearly resolution**
  In the original data, a timestamp is provided for each record. However, it is unknown whether the water table was measured at that specific timestamp or if it represents the mean over a specific day, month, or year. For more straightforward analysis, the temporal resolution was harmonized to ensure equal time step intervals. The time series were aggregated to either daily, monthly, or yearly means. The rationale applied to this time step aggregation was: When a time series is aggregated, there should not be more than 10% of the time series with data gaps (missing days, months or years). Consequently, 90% of the time series should be at least in that certain resolution. Specifically, this means that 90% of the time intervals between the records should be at least daily to be classified as daily time series. In case of monthly time series, we decided that 90% of the time intervals should be at least 40 days. We made this decision because many time series have a loose monthly measurement schedule between 20 and 50 days. These would probably be classified as yearly time series with a threshold of 31 days although such a loose schedule will not necessarily cause gaps in the monthly data. We chose 40 days as a compromise so that only a record on the 22^nd^ day of a month or later followed by a 40 day time interval to the next record will lead to a gap (1 missing month). This also means that for monthly data more than 10% gaps are still possible in case of the described example. Every other time series where the 90th percentile of the time step intervals between the records is larger than 40 days is aggregated to a yearly resolution. For transparency, the number of records aggregated to daily, monthly, or yearly means is flagged in cases where time series do not solely contain the first day of the month [year] (e.g., 2010-01-01) or the zeroth hour of the day (e.g., 2010-01-01 00:00:00). In latter case, we assume that the records already represent aggregated means of multiple records whose number is unknown (aggregated_from_n_values = ‘NA’).
5. **Capping the gap fraction and gap length**
  GROW^56^ provides an additional data field for every time series where gaps are linearly filled. To minimize the impact of the gap-filling on the statistical characteristics of the time series, the total gap fraction and gap length are capped accordingly. As the total gap fraction is determining most of the thresholds in the preprocessing (temporal aggregation, single gap length, plateau length), we performed a sensitivity analysis on the gap fraction and tested 10%, 20% and 30%. Based on this, we selected 10% because this threshold guarantees the highest data quality while resulting in only minor variations in processing outcomes and dataset characteristics across the different threshold settings. The total number of time series after preprocessing (steps 1–10) differed by fewer than 200. The proportions of the three temporal resolutions (daily, monthyl, yearly) varied by no more than 4%. With a threshold of 10%, there are 4% fewer daily or monthly time series after preprocessing compared to a threshold of 30%. The percentage of data lost due to capping of gap fraction and gap lengths varied by less than 1%. The full results of the sensitivity analysis are presented in the Supplements Table S.1-4.
  - The total allowed gap fraction is 10%. For time series that exceed this threshold, the longest sequence of years with less than 10% gaps is extracted for daily and monthly data. For yearly data, only the sequence without gaps is extracted.
  - The maximum allowed gap length is derived from the total gap fraction of 10%. We selected the thresholds so that the gap lengths are not longer than 10% of a month (daily data), year (monthly data), or the total time series length (yearly data). Consequently, the maximum allowed gap length is 3 days for daily data, 1 month for monthly data, 0 gaps for yearly data up to 9 years, 1 year for yearly data between 10 and 19 years long, and 2 years for time series that are 20 years or longer. To account for natural variance within time series, the allowed gap length can be smaller when a time series exhibits high scatter. Based on the autocorrelation of every time series, the allowed gap between two steps is determined to be the maximum time lag for which the autocorrelation is equal to or larger than 0.6 (spearman rank correlation coefficient^61^), considering only time lags up to the point where the correlation coefficient becomes negative for the first time. Still, the maximum gap length is the upper limit. If there is no autocorrelation exceeding 0.6 within the considered time lags, no gaps are allowed.The sequence is extracted in which the gaps do not exceed the individual gap length threshold, and the preprocessing is continued.
6. **Flagging negative depth to groundwater records**
  Depth to groundwater records that are negative, indicating that the hydraulic head is above ground, naturally exist in the case of flowing artesian wells^62^ or riparian zones^63^. However, we cannot manually check all 2,528 wells with negative values to separate plausible data from potential measurement errors. Therefore, time series with some negative records and time series with solely negative records were flagged.
7. **Flagging autocorrelation of time series**
  The autocorrelation is calculated again.
8. **Flagging outliers and change points with DBSCAN**
  Outliers (noise or spikes^64^) and change points (breaks - sudden increases or decreases of a variable^64^) in time series can result from sensor failures. However, this can also be attributed to the natural variability of highly dynamic aquifers, weather extremes, or anthropogenic pumping. We flagged time series that potentially contain outliers and/or change points. The purpose of the flag is to be a first point of reference for anomalies (i.e. measurement errors, human impacts, extreme events). For actual time series repair, knowledge about the study area and visual inspection to validate detected outliers are highly recommended^59,60,65^. Users who want to correct the time series can use the flag for prioritization. Instead of correction, the flagged time series could also be filtered out. We implemented an outlier and change point detection with the DBSCAN algorithm^66^ as in Nolte *et al*.^59^ DBSCAN is a density-based clustering method with noise^66^. The algorithm groups the data based on their groundwater values in different clusters and noise. Every time series that contains one cluster and noise or multiple clusters, is flagged to contain outliers and/or change points. The single records assigned to noise or a minority cluster are flagged as outliers as well. The two determining parameters in DBSCAN are set to *Eps = 2x standard deviation of time series* and *MinPts = 0.5x total number of records in time series*. Eps defines the maximum distance between two data records to be considered neighbours. MinPts determines the number of neighbours required for a data record to be considered as core point^66^.
9. **Flagging uninterrupted sequences of static water table values as potential measurement error**
  Uninterrupted sequences of the exact same water table (plateaus) are flagged in each time series with their plateau length. Like the flagging of outliers and change points, this indicates possible measurement errors. Plateaus are treated as potential data gaps and deemed problematic when they exceed thresholds for the maximum allowed gap length. Accordingly, sequences of the exact same water table in sequential time steps are flagged as plateaus starting from 4 days for daily data, 2 months for monthly data, 2 years for yearly data shorter than 20 years and 3 years for yearly data that is 20 years or longer.
10. **Calculating Mann-Kendall trend direction and Sen’s slope**
  The trend direction and the Sen’s slope, in case of a significant trend (p-value < 0.05), were derived for each time series. If the time series was flagged to be autocorrelated (see step 4), the Hamed and Rao Modified Mann-Kendall test was used to perform the trend analysis. Here, a variance correction is applied to account for serial autocorrelation^67^. Otherwise, the original Mann-Kendall test from the python package pyMannKendall was utilized^68^. Independent from the reference point, an increasing trend is associated with a rising water table and vice versa. Therefore, the trend direction of time series containing depth to groundwater records was switched (increasing < - > decreasing) so that, for example, a rising water table (decreasing depth) is flagged as ‘increasing’. Additionally, the sign of the trend slope is switched for these cases. It should be noted that the effect size and significance of a test should be critically scrutinized because of its dependence on sample size^69^.
11. **Adding further data flags**
  Additional flags were generated per time series and added to the well attributes (see Table 2). That are: the mean and median water table per time series, the first and last date of a time series, the length of the time series in years and the median number of records in the original data that were aggregated to one record in GROW^56^.
12. **Removing duplicates in well attributes**
  There are three types of duplicates apparent in the dataset. Duplicate by well ID and country (a), duplicate by location and groundwater time series (with different ID; b), and duplicate from the Jasechko *et al*.’s^12^ dataset with same data source but altered attributes and records (c):
  - To assign the attributes to the time series a unique key in every table is necessary. As time series and attributes in the source data were sorted in different folders by country, the well ID and country can be used to merge the data. Consequently, duplicate records with duplicate ID from the same country are both deleted as it is not feasible to resolve which IDs should be assigned to each time series.
  - Furthermore, wells with the same coordinates, starting date, ending date and mean water table under two different IDs were identified as duplicates. In this case, only one well record was removed.
  - 108,328 wells of Jasechko *et al*.’s study^12^ are part of the groundwater source dataset Groundwater Observation Repository^57^. A subset of them overlaps with wells from the GGMN^47^ as they come from the same original data provider. However, the time series published by Jasechko *et al*.^12^ were modified. That means that they are not identical with the original data from the provider. Well coordinates were truncated, different well ID’s were used and the time series were aggregated to a yearly resolution. Thus, the mean water table as well as the start and end date could deviate from the duplicate. For this reason, the detection method in (b), above, was not applicable for those cases. To detect and remove these duplicates, the latitude and longitude coordinates were rounded to one decimal place and the mean water table was rounded to zero decimal places. Afterwards, duplicates within these three parameters that originate from different organizations (data origin) were filtered. Within this subset, all duplicates originating from Jasechko *et al*.^12^ were removed (9,712 wells). We rounded the coordinates and the mean water table very strongly because we would rather remove more time series that are no duplicates than keep real duplicates. This rounding was only applied to find duplicates and is not applied to the final dataset.
13. **Checking for coordinates outside the plausible range**
  The coordinates of the well location are projected in WGS 84. In this coordinate system, the latitude must be between −90° and 90°, and the longitude between −180° and 180°. We searched for outliers to remove them from the dataset because a manual data correction is not feasible for such a large dataset. No outliers were found.
14. **Removing empty attribute columns**
  34 columns that were either empty or contained only one test entry were removed.
15. **Trimming time series based on kept attributes**
  Time series whose well attributes were lost during the preprocessing (steps 12-13) were removed.

**Table 2:** Overview of preprocessing steps for the groundwater time series with the number and percentage of discarded/flagged time series and the flags created per step.

Preprocessing step	Percentage and (number) of discarded time series	Percentage and (number) of flagged time series	Created data flag
1. Discarding time series with less than 2 records	8.81% (22,136)	0	/
2. Reconciling records to ensure that only one groundwater reference point elevation is provided per time series	0.53% (1,342)	0	/
3. Removing numeric placeholders for missing records	0	0	/
4. Temporal aggregation of time series to either daily, monthly, or yearly resolution	1.86% (4,680)	1 - 85% YS (174,337), 9% MS (18,670), 6% d (11,285) 2 -44% not-NA (90,757)	*1 - interval* [d,MS,YS] 2 - *aggregated_from_n_values* [number]
5. Capping the gap fraction and gap length a) gap fraction b) gap length	a) 0,64% (1,617) b) 2,42% (6,094)	a) 100% not-NA	*a) gap_fraction* [number]
6. Flagging negative depth to groundwater records	0	96% No (196,747), 0.8% Some (1,617), 0.4% All (911)	negative_signs_wtd [‘No’,’Some’,’All’]
7. Flagging autocorrelation	0	16% True (33,142)	*autocorrelation* [True/False]
8. Flagging outliers and change points with DBSCAN	0	6% True (14,524)	*outliers_change_points* [True/False]
9. Flagging uninterrupted sequences of the exact same water table as potential measurement error	0	1 - 11% True (22,623) 2 - 100% not-NA	1 - Attribute table: *plateaus* [True/False] 2 - Time series table: *plateaus* [plateau length in time steps]
10. Calculating Mann-Kendall trend direction and Sen’s slope	0	1 - 66% no trend (134,157), 22% decreasing (45,506), 12% increasing (24,629) 2 - 34% not-NA (70,135)	*1 - trend_direction* [‘no trend’, ‘decreasing’, ‘increasing’] *2 - trend_slope* [m/year]
11. Adding further data flags	0	100% not-NA	*starting_date* [date], *ending_date* [date], *length_years* [number], *aggregated_from_n_values_median* [number], *groundwater_mean_m* [number], *groundwater_median_m* [number]

All data flags are listed in Table 6.

**Table 3:** Overview of preprocessing steps for the groundwater attributes with the number and percentage of discarded/flagged wells and the flags created per step.

Preprocessing step	Percentage and (number) of discarded time series	Percentage and (number) of flagged time series	Created data flag
12. Removing duplicates in well attributes a) Well ID – country duplicates b) Location and groundwater time series – duplicates c) Jasechko wells - duplicates	a) 0.5% (1,256) b) 0.1% (255) c) 3.87% (9,724)	a) 0 b) 0 c) 0	a) / b) / c) /
13. Checking for coordinates outside the plausible range	0	0	/
14. Removing empty attribute columns	0	0	/
15. Trimming time series based on kept attributes	0	0	/

All data flags are listed in Table 6.

### Earth system variables

The set of Earth system variables added to GROW^56^ are based on a selection of processes most relevant to water table dynamics (see Table 1). To expand the groundwater time series data with these variables, data products were identified that fulfil the following criteria. (1) The data product must be published under a license that permits modification and redistribution. (2) Instead of using multiple regional data products per variable, only global data products were considered to enable comparability within GROW^56^. (3) When choosing between different data products, preference was given to data products based on the following priorities (in decreasing order): Greater temporal coverage, outperforming comparable data products as indicated in the literature, higher temporal resolution, and higher spatial resolution. (4) Furthermore, variables and data products were chosen that could either be downloaded automatically or did not need extensive preprocessing.

In total, thirty-six Earth system variables were selected to complement the groundwater attributes and time series. The added Earth system variables were derived from observation-based data, reanalysis data, or modelled data. Table 4 (attributes) and Table 5 (time series) give an overview of every variable and its source. Additional information about data access, spatial or temporal resolution, original units, and record coverage is provided in the Supplements Tables S.2-1, S.2-2. The variables are organized by the six Earth system components: Atmosphere, Hydrosphere, Geosphere, Biosphere, Cryosphere, and Anthroposphere (Table 1).

**Table 4:** Overview of all added Earth system attributes (no temporal dimension), their unit and data source.

Earth system variable	Column name in GROW	Unit in GROW	Data source (Source type)
Koeppen-Geiger classification	koeppen_geiger_class	/	CHELSA v2.1 - kg0^118^ (Downscaled Reanalysis)
Hydrobelt	hydrobelt_class	/	Meybeck *et al*.^119^ (Model + Observation-based)
Ground elevation	ground_elevation_merit_m_asl	m	MERIT DEM v1.0.3^92^ (Observation-based)
Topographic slope	topographic_slope_degree	°	Geomorpho90m^120^ (Observation-based)
Rock type	rock_type_0-100_m_class	/	GLiM v1^77^ (Observation-based)
Aquifer type	aquifer_type_class	/	WHYMAP WOKAM^76^; GLiM v1^77^(Observation-based)
Permeability for 0-100 m depth	permeability_0-100_m_m-2	m²	GLHYMPS 2.0^121^ (Observation-based)
Total porosity for 0-100 m depth	total_porosity_0-100_m_fraction	/	GLHYMPS^122^ (Observation-based)
Soil texture class in topsoil (0-30 cm)	soil_texture_0-30_cm_class	/	HiHydroSoil v2.0^123^ (Model)
Soil texture class in subsoil (30-200 cm)	soil_texture_30-200_cm_class	/	HiHydroSoil v2.0^123^ (Model)
Saturated hydraulic conductivity of topsoil (0-30 cm)	soil_saturated_conductivity_0-30_cm_cm_d-1	cm/day	HiHydroSoil v2.0^123^ (Model)
Saturated hydraulic conductivity of subsoil (30-200 cm)	soil_saturated_conductivity_30-200_cm_cm_d-1	cm/day	HiHydroSoil v2.0^123^ (Model)
Distance between perennial streams	distance_perennial_streams_m	m	Cuthbert, Gleeson *et al*.^54^ - 0.1 cubic metres per second flow threshold (Observation-based)
Drainage density	drainage_density_m-1	m^−1^	HydroRivers^78^; BasinATLAS Level 9^44^ (Observation-based)
Glacier cover in surface catchment	glacier_cover_fraction	/	BasinATLAS Level 9^44^ (Observation-based)
Permafrost cover in surface catchment	permafrost_cover_fraction	/	BasinATLAS Level 9^44^ (Model)
Average days per year with snow cover	days_with_snow_cover_average_days_year-1	days/year	ERA5-Land^79,94^ (Model)
Groundwater dependent ecosystems	groundwater_dependent_ecosystems_class	/	Huggins *et al*.^124^ (Multi-source)
Main land use	main_landuse_class	/	Volkholz & Ostberg 2024^125^ (Model)
Groundwaterscapes	groundwaterscapes_ID_class	/	Huggins *et al*.^126^ (Multi-source)

The source type “Observation-based” also includes remote sensing products. A table with additional information about data access, spatial resolution, unit and records coverage is provided in the Supplements Table S.2-1.

**Table 5:** Overview of all added time-varying Earth system variables, their unit, and data source.

Earth system variable	Column name in GROW	Unit in GROW	Data source (Source type)
Precipitation	precipitation_mswep_mm_year-1	mm/year	MSWEP V2^70^ (Multi-source)
Precipitation	precipitation_gpcc_mm_year-1	mm/year	GPCC^71^ (Observation-based)
Potential Evapo-transpiration	potential_evapotranspiration_era5_mm_year-1	mm/year	ERA5-Land^79,94^ (Model)
Potential Evapo-transpiration	potential_evapotranspiration_gleam_mm_year-1	mm/year	GLEAM4^75^ (Model)
Actual Evapotranspiration	actual_evapotranspiration_mm_year-1	mm/year	GLEAM4^75^ (Model)
Interception loss	interception_mm_year-1	mm/year	GLEAM4^75^ (Model)
Air temperature	air_temperature_°C	°C	ERA5-Land^79,94^ (Reanalysis)
Snow depth	snow_depth_m	m	ERA5-Land^79,94^ (Model)
Days per year with snow cover	days_with_snow_cover_days_year-1	days/year	ERA5-Land^79,94^ (Model)
NDVI	ndvi_ratio	/	1981-2013: AVHRR NDVI^91^; 2014-2024: VIIRS NDVI^127^ (Observation-based)
Leaf area index of low vegetation	lai_low_vegetation_ratio	/	ERA5-Land^79,94^ (Model)
Leaf area index of high vegetation	lai_high_vegetation_ratio	/	ERA5-Land^79,94^ (Model)
Total water withdrawal for industrial use	withdrawal_industrial_m3_year-1	m³/year	Wada *et al*.^128^ - indww_histsoc_annual (Model)
Total water withdrawal for domestic use	withdrawal_domestic_m3_year-1	m³/year	Wada *et al*.^128^ - domww_histsoc_annual (Model)
Fraction of urban areas	urban_area_fraction	/	Volkholz & Ostberg^125^ - landuse-urbanareas_histsoc_annual (Model)
Fraction of pastures	pastures_fraction	/	Volkholz & Ostberg^125^ - landuse-totals_histsoc_annual (Model)
Fraction of rainfed cropland	cropland_rainfed_fraction	/	Volkholz & Ostberg^125^ - landuse-totals_histsoc_annual (Model)
Fraction of irrigated cropland	cropland_irrigated_fraction	/	Volkholz & Ostberg^125^ - landuse-totals_histsoc_annual (Model)
Fraction of forests and natural vegetation	forests_natural_vegetation_fraction	/	Volkholz & Ostberg^125^ - landuse-totals_histsoc_annual (Model)

The source type “Observation-based” also includes remote sensing products. A table with additional information about data access, spatial and temporal resolution, unit and records coverage is provided in the Supplements Table S.2-2.

It is desirable to associate groundwater time series with precipitation and potential evapotranspiration records as comprehensive as possible as these are widely recognized to be the most important, large-scale, non-anthropogenic variables driving groundwater dynamics^12,45,48,54^. To increase temporal coverage and account for some uncertainty in precipitation and potential evapotranspiration data products, two complementary products per variable were added to GROW^56^, each one as separate column. For the MSWEP 3-hour precipitation data product^70^ multiple precipitation data sources (gauge-measurements, satellite, or reanalysis) were merged. Per location that data source with the highest quality was selected. MSWEP has a high temporal resolution and can cover the most recent groundwater records as it is almost updated in real-time with a latency of 3 hours. Following this, we selected this product due to its high data quality standards, high temporal resolution, and coverage of the most recent records. However, it does not cover records before 1979. Early groundwater records are therefore supplemented with monthly precipitation data from GPCC^71^, which can cover records before 1901 (begins in 1891) and have a higher spatial resolution (0.25°) in comparison to other products that start before 1901 like Observed Land Surface Precipitation Data (2.5°)^72^. Both datasets cover 99.99% of the groundwater time series, while MSWEP only achieves a record coverage of 98% (Supplements Table S.2-2). Regarding potential evapotranspiration, the ERA-5 Land dataset^43^ covers the longest time span (1950-now) but is known to overestimate potential evapotranspiration^73,74^, especially for arid regions^43^. To account for this uncertainty, we included another product derived from a different data source. The Global Land Evaporation Amsterdam Model (GLEAM4) was selected because it calculates potential evapotranspiration with Penman’s equation^75^ unlike ERA5-Land. Latter’s potential evapotranspiration is simulated with a land surface model ‘for agricultural land as if it is well watered and assuming that the atmosphere is not affected by this artificial surface condition’^43^.

To merge Earth system variables with each groundwater well, the value of the raster pixel or polygon containing the well’s location is extracted and associated with that well. This means that the variables are not measured at the explicit location of a well, but are, due to the nature of raster or polygon data, averaged over a certain region. With this in mind, the results should be understood as local to regional characteristics, representative for the respective spatial resolutions of the Earth system variables (with a spatial resolution range from 3 arc seconds to 0.5°, Supplements Tables S.2-1, S.2-2). Since the well coordinates are projected in WGS 84, the used data products were reprojected to WGS 84 if they were initially available in a different coordinate system. The static variables (no temporal dimension) were added as columns in the attributes table. The time-varying variables were first aggregated to the temporal scale of each individual groundwater time series and merged by ID and date to the time series table.

Some variables were only available at monthly (precipitation from GPCC) or yearly resolutions (water withdrawal, land use fractions and days per year with snow cover). We include these data also for daily and monthly time series as they can be used to split the dataset based on thresholds. Therefore, these products are combined with daily [monthly] groundwater data so that each day [month] within a specific month [year] is matched with the corresponding value for that month [year].

The time series with different temporal resolutions are stored in a single table, all with the same unit of quantity (mm/year) for parameters such as precipitation. This enables the straightforward derivation of further aggregations across different time series resolutions, subsets, and statistics, regardless of the temporal resolution. Because 85% of the time series have a yearly resolution, quantitative units are provided for an annual period (mm/year). This means that monthly and daily time series also have units of mm/year.

In the following, we briefly describe the processing steps needed for a few variables that require additional processing:

- The aquifer type [porous, fractured, porous/fractured, karst and water_body] was estimated based on the World Karst Aquifer Map (WOKAM)^76^ and the GLiM^77^. Wells, which are located in a WOKAM-karstifiable region, are assigned ‘karst’ as aquifer type. For the rest of the wells the following classification based on GLiM rock types was applied. The aquifer type is porous when the rock type is unconsolidated sediments (GLiM class: su). As aquifers in mixed sedimentary rocks and siliciclastic sedimentary rocks (GLiM class: sm,ss) can be porous or fractured, wells within those rock types are assigned ‘porous/fractured’. The class ‘water_body’ is directly transferred from GLiM’s class ‘water bodies’. The rest is classified as 'fractured'.
- Drainage density was calculated by dividing the sum of all river lengths in a catchment by the area of that catchment. HydroRIVERS^78^ and BasinATLAS (level 9)^44^ were utilized for that purpose. Both datasets were reprojected into the global metric coordinate system World-Eckert-IV before calculating the fraction of river lengths sum per basin area.
- The (average) days per year with snow cover were derived from the daily ERA5-Land^43,79^ snow cover data. For each year and each well, the number of days with snow cover >0 m was calculated and added to the time series table. The variable ‘days per year with snow cover’ was set to NA if any data were missing for that year. Otherwise, missing values would have been interpreted as 0 days, which would have biased the results. The multi-year average was added to the attributes table.
- The main land use type in the attributes table is that land use whose average fraction over time is the highest.

## Data Records

The final GROW dataset^56^ consists of two files: A table containing time series data and a table with static attribute data. Both can be downloaded as either a comma-separated values (CSV) or parquet file from Zenodo: 10.5281/zenodo.15149480 and used under a Creative Commons Attribution Non-Commercial ShareAlike 4.0 International License (CC-BY-NC-SA 4.0). Some groundwater time series and Earth system variables are published under an individual license, but none of them is more restrictive than CC-BY-NC-SA 4.0. Please note the full license information provided in the Readme file on Zenodo. A dataset documentation on Zenodo (Readme file) provides descriptions of every variable/column for both tables. To subset the data, the attributes table can be filtered based on user-specified criteria, and afterwards the time series table can be filtered for the remaining well IDs in the attributes table. A total of 34 data flags are available to help with this filtering (Table 6). With data flags like the average water table (‘groundwater_mean_m’), the number of values that were aggregated to one water table record (‘aggregated_from_n_values’) or water table records that were detected to be potential outliers/change points (‘outliers_change_points’), users can select data based on their individual (quality) needs. For example, a data subset is possible in which yearly data was either not aggregated (always the first day of the year) or aggregated from at least four values. To provide another example, a user can decide to drop all time series that contain outliers and change points (7% of the time series) or plateaus (11% of the time series). A best-practice script for data selection can be viewed on GitHub (https://github.com/EarthSystemModelling/GROW/blob/main/usage_example.py) or Zenodo: 10.5281/zenodo.15149480.

**Table 6:** List of all data flags that are added to the time series and attributes table.

Data flags in time series table	Data flags in attributes table
⦁ interval (d/ MS/ YS) ⦁ year (e.g., 2008) ⦁ month (e.g., 2008-02) ⦁ aggregated_from_n_values (e.g., 1) ⦁ outliers_change_points (True/ False) ⦁ plateaus (length of plateau in time steps, e.g., 10)	⦁ interval (d/ MS/ YS) ⦁ starting_date (e.g., 2010-01-01) ⦁ ending_date (e.g., 2013-03-08) ⦁ length_years (in years, e.g., 9) ⦁ autocorrelation (True/ False) ⦁ aggregated_from_n_values_median (e.g., 2) ⦁ gap_fraction (e.g., 0.1) ⦁ negative_signs_wtd (All/ Some/ No) ⦁ outliers_change_points (True/ False) ⦁ plateaus (True/ False) ⦁ trend_direction (no trend/ increasing/ decreasing) ⦁ trend_slope_m_year-1 (e.g., 0.9) ⦁ groundwater_mean_m (e.g., 34) ⦁ groundwater_median_m (e.g., 34) [feature_type, purpose, status, description, latitude, longitude, provider_ground_elevation_m_asl, top_of_well_ elevation_m_asl, total_drilling_depth_m, country, address, aquifer_name, confinement, organisation, manager, license, parameter (renamed to ‘reference_point’)]

[The flags in brackets] were already part of the GGMN^47^ and Groundwater Observation Repository^57^ datasets.

## Data Overview

**Fig. 1:**
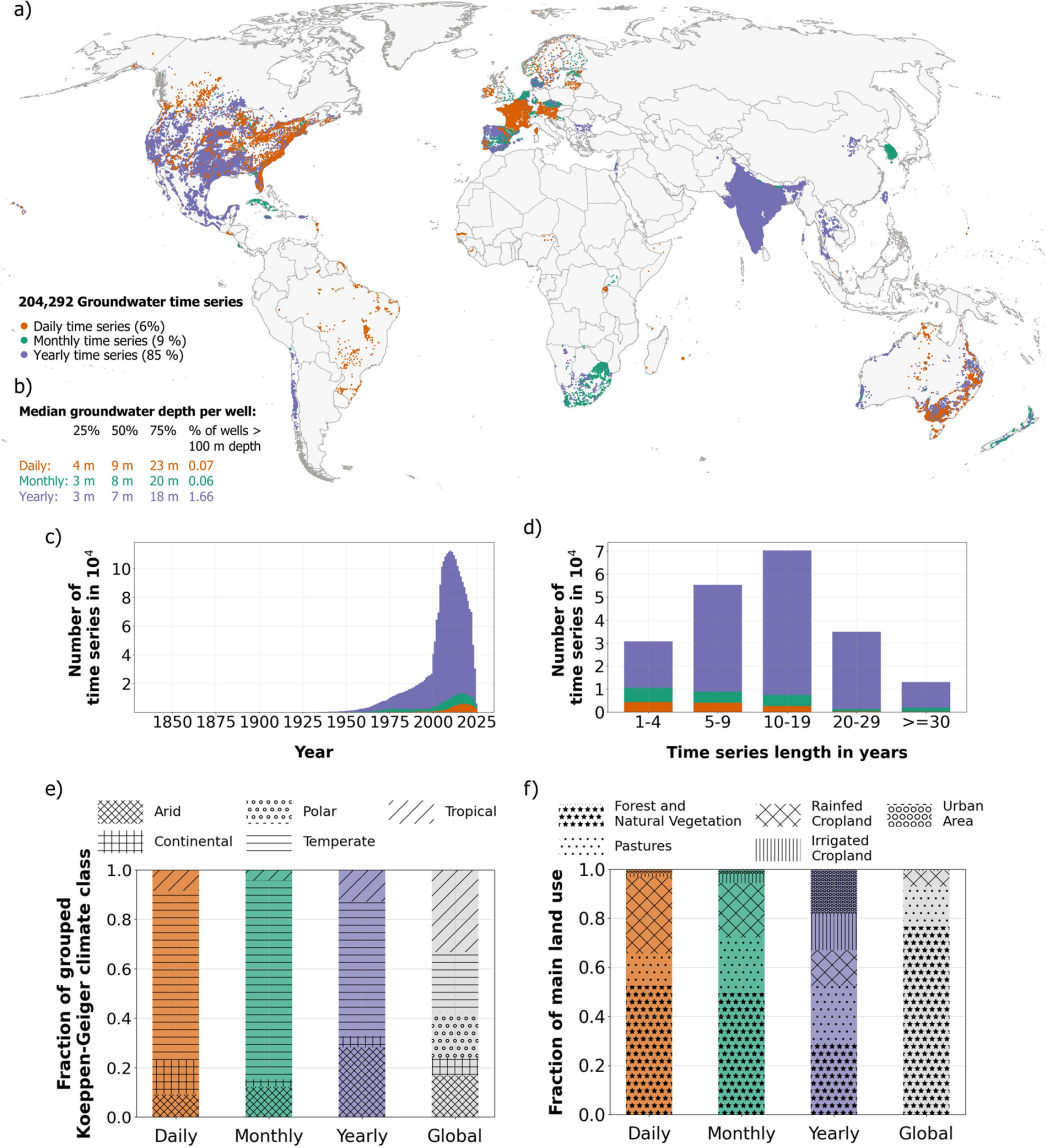
Descriptive overview of groundwater data and selected Earth system variables contained in GROW. (**a**) The map displays the locations of all time series, classified by temporal resolution. The color indicates whether a time series has a daily (orange), monthly (green) or yearly (purple) resolution. (**b**) Additionally, the 25% percentile, 50% percentile and 75% percentile of the median depth to groundwater per well and the percentage of wells with a median groundwater depth above 100 m is given for each temporal resolution. Below, (**c**) the number of time series available per year, and (**d**) the number of time series per length class in years are displayed. At the bottom, the distributions of selected Earth system variables in GROW compared to their respective global distributions are shown. The stacked bar charts show (**e**) the fraction of grouped Koeppen-Geiger climate classes and (**f**) the fraction of main land uses in GROW.

A map showing the global distribution of the wells included in GROW^56^ (Fig. 1a) highlights the geographic patterns in data availability. This mapping reveals a bias to locations within North America (51% of the wells), India (17% of the wells), Europe (13% of the wells), and Australia (10% of the wells), which contain 91% of the data records (Supplements Figure S.1–6). With a median depth to groundwater of 8 m, GROW^56^ represents shallow groundwater (here defined with depth to groundwater below 10 m like in Cuthbert *et al*.^54^). 34% of the time series are between 10 and 19 years long (70,302). 17% of the time series are 20 years and longer (34,504) with a maximum length of 135 years. Time series of 20 years and longer are more common to have a yearly resolution (91%) compared to all time series (85%). On the other hand, short time series of 1 to 4 years have the highest fraction of daily (9%) and monthly (14%) time series among the length classes (Fig. 1d).

The characteristics of the Earth system variables in GROW^56^ differ from their global distributions (Fig. 1e,f). The global distributions are derived from all pixel values in the respective raster data with area-weighting to correct for the area distortion of the WGS 84 coordinate system. The area-weighting is described in more detail in the Supplements section S2. Based on the Koeppen-Geiger climate classification^80^, the wells in GROW^56^ are disproportionately located in temperate (55%–80% in GROW^56^ vs. 25% globally) and arid (9%–28% in GROW^56^ vs. 17% globally) climates (Fig. 1e). The main land use in GROW^56^ is characterized by a higher anthropogenic influence compared to the global land use distribution (Fig. 1f). While the proportion of urban areas is below 1% globally, it is very prominent in the yearly resolution data with 15%. Compared to global data, the proportion of agriculturally used land (pastures and cropland) is higher in GROW^56^ by 23%, 26%, and 32% in the daily, monthly, and yearly data. The wells are located at lower elevations (median ground elevation of 105 m in comparison to 366 m globally; Supplements Figure S.2-3). The distributions of the rest of the Earth system variables in GROW^56^ compared to their global distributions are given in the Supplements section S2.

## Technical Validation

### Quality control of groundwater data

The preprocessing of the groundwater time series ensures that the data are harmonized, gap-limited, and checked for potential measurement errors, incorrect coordinates, and duplicates. In total, 47,104 of 251,396 time series (19%) were discarded due to not meeting the quality criteria (Tables 2, 3). 9% of the time series were rejected because they were empty or contained only one record. The largest data loss in the attribute tables was due to duplicates originating from Jasechko *et al*.^12^ (4%). Examples of discarded time series are given in the Supplements section S1. Of the remaining groundwater time series, 13,751 contain gaps (7% of all time series in GROW^56^). 34% of these time series are in a monthly resolution, and 36% are in a daily resolution. Therefore, higher-resolution time series were found to possess incomplete records more often compared to time series with yearly time steps. The median gap fraction of the incomplete time series is 1.7%. Yearly data have the highest median gap fraction (4.2%), and daily data have the lowest (0.1%). The median gap lengths for daily, monthly and yearly data are 1 day, month, or year long. The total gap fraction and single gap lengths per well do not exceed the defined thresholds. A figure with the distribution of the gap fraction and mean gap length per time series and temporal resolution is displayed in the Supplements Figure S.1-7.

### Uncertainty sources

The observed groundwater time series as well as the Earth system variables contain data gaps and uncertainties that propagate to GROW^56^. In the following, we give an overview with examples of uncertainty sources in the dataset. Uncertainties in groundwater observations arise from a lack of knowledge about metadata, the exact measurement time, and spatial data bias. Metadata such as measurement method (not reported at all), borehole construction details (only drilling depth provided for 5% of wells), and aquifer confinement (available for 6%), are largely incomplete across the dataset. Dependent on the measurement method, uncertainties associated with the groundwater data and the susceptibility to certain measurement errors differ. Reading precision is lower for manual measurements, which are mainly performed with dip meters. On the other hand, automated pressure measurements hold the risk of sensor failures and internal clock drifts. Following, errors like spikes, water table drifts and erroneous timestamps are more likely with automated measurements^64^. The point of head error is further influenced by construction details like screen length or borehole inclination^64^. As the measurement methods and construction details, except for drilling depth and top of the well elevation, are unknown for the GROW wells, the quality of the groundwater data cannot be assessed and compared^64^. The lack of knowledge about confinement hinders a correct interpretation of the water table records as water table head. Further, it is impossible to determine whether water table records above the ground are measurement errors or belong to artesian wells. Knowing whether an aquifer is confined or unconfined is important for groundwater analysis as many theoretical concepts only apply to either unconfined or confined conditions. Other than that, the interpretation of water table changes should be adapted in regard to confinement. For example, storage changes lead initially to stronger head declines in confined compared to unconfined aquifers^81^. As the groundwater time series are provided by different data holders, the preprocessing of the data is challenging. For example, it is unclear whether the timestamp of a record indicates the exact day of measurement or an already aggregated mean for the respective period (daily, monthly, or yearly). Due to a lack of groundwater observations, the dataset is not able to represent the globally possible value ranges of all variables (Fig. 1). Groundwater observations are recorded at relatively shallow depths less than 10 m and in places with high anthropogenic impact (agriculture as primary land use) because wells are built where water is cost-effectively available (shallow), needed (high anthropogenic impact), and where funds for recording and sharing observations are available (wealthy countries)^82^. Groundwater time series are not available for many water-scarcity hotspots like Pakistan, northeastern China and the Middle East^11,13^. This suggests that the calculated proportion of decreasing trends in GROW^56^ (22%) is likely underestimating the actual global decreasing trends. On the other hand, the high anthropogenic impact might skew the trend direction to more decreasing trends. Less data is available for processes related to high altitudes, tropics, polar zones, snow accumulation, glaciers, and permafros. Therefore, it could be more difficult to achieve statistical significance in an analysis regarding those processes. A bias of *in situ* data towards an anthropogenic impact and high-income countries is a common problem in hydrological research^83,84^. Along comes a higher proportion of research and data sets originating from regions with more *in situ* data and lower risk of adverse impacts. This inhibits a truly global perspective^36,83^. GROW^56^ makes the unavailability of *in situ* measurements in certain regions and further implications on groundwater analysis visible.

An additional source of uncertainty in GROW^56^ originates from the Earth system variables that were combined with the observed groundwater data. Uncertainties in the modelled variables arise from the oversimplification of Earth system processes and differing assumptions on how they are implemented^20,24,33,34^. Especially, human impacts are complex and difficult to quantify^35,85^. This is emphasized for the water withdrawal product in GROW^56^, which was derived from a multi-model ensemble^86^. Wada *et al*.^86^ found substantial differences between the three global models simulating water withdrawal trends for the industrial sector. Among other things, the authors attributed the discrepancy to differing model approaches (e.g., the distinction between electricity and manufacturing water use). Another source of uncertainty is input data^31,33,85,87^. For example, GLEAM4 primarily uses reanalysis and satellite data as simulation input^75^, which pass their uncertainties on to the model output. Finally, a lack of observations^48,88^, the non-applicability of local methods that are based on expert knowledge, and even computational limits^29,88^ inhibit comprehensive calibration and evaluation methods. Notably, field measurements of actual evapotranspiration^89^ and actual water withdrawal^86^ are scarce. There are also no satellite observations as both parameters cannot be directly measured with the sensors^89,90^.

There are other sources of uncertainty coming from Earth system variables that are not modelled. Satellite-based data products, such as AVHRR NDVI^91^ or MERIT DEM^92^, have the advantage of wide temporal and spatial coverage^93^. However, satellite observations are no direct point measurements but an average characteristic over a particular spatial resolution. In this sense, the MERIT DEM lacks detailed topography, such as small lakes or levees^92^. Although reanalysis products (e.g., ERA5-Land^94^) seem less uncertain than modelled data because they have incorporated observations, they contain their own pitfalls: They are sensitive to data availability, measurement errors, and spatial as well as temporal representativeness. Therefore, data-scarce regions and times are less reliable^93^. The same applies to gauge-based data products like GPCC, where observation gaps are interpolated^93^. Uncertainties in categorical data like geological maps (e.g., GLiM) are not characterized by magnitude of deviation but by ambiguities in group classification. In that case, wrong mapping and deviations between regional geological maps are examples of uncertainty sources^95^. Furthermore, structural details like fault zones are neglected on the global scale^96^, and there is no mapping of vertical heterogeneity^77,96,97^.

Lastly, the combination of point observations with gridded data creates its own uncertainty: Commensurability errors occur when point observations are compared with grid values that represent several square kilometers. Naturally, extreme events are often not fully captured in these contexts. The greater the mismatch in (spatial) scale, the higher the commensurability error^29,93^.

Here, we present a first static version of GROW^56^, marking the commencement of a long-term initiative. In this initiative, we envision to develop a dynamic version of the dataset^56^ that continually grows and benefits from data additions of the global community. The addition of groundwater time series, especially from regions that are underrepresented, reduces the spatial bias and increases global representativeness in such a dataset. We invite the global groundwater community to contribute data to IGRAC’s GGMN^47^ and Groundwater Observations Repository^57^. It is currently the only dynamically growing global dataset of groundwater time series that offers the option to add a comprehensive set of metadata.

## Usage Notes

We envision GROW^56^ to be used for integrated groundwater analysis that addresses the drivers behind groundwater dynamics. Here, we provide guidance and usage examples. The groundwater information is either given as water table depth or level dependent on the time series. To derive both for the same time series, the ground elevation from either the provider or the MERIT DEM^92^ can be used. This is described in more detail in the Readme file on Zenodo (10.5281/zenodo.15149480).

Although the gaps in the dataset incentivize analysis of well-represented environmental settings (e.g., arid or temperate climates and low elevations; see Fig. 1), we encourage the inclusion of underrepresented settings in analyses using GROW^56^. To reduce bias, users could trim the dataset to approximate the global distribution of one or multiple Earth system variables. The descriptive statistics comparing the distribution of the Earth system variables in GROW^56^ with their respective global distributions provide a reference for this (Supplements section S2).

GROW^56^ can help to understand groundwater dynamics within local to regional contexts captured by the data products from which the Earth system variables originate (see Tables 4, 5). Site-specific analysis should be carried out with caution. For example, localized pumping conditions cannot be investigated with the Earth system variables ‘Total water withdrawal for domestic and industrial use’. They represent modelled total water withdrawals in the 0.5° raster pixel in which the well is located. With this dataset and the limited metadata, actual human disturbance in GROW^56^ can only be derived from the groundwater time series. We are not aware of an algorithm that can adequately do that for global-scale data. A method for regional data was presented by Lehr & Lischeid^60^, but local knowledge and visual control were still necessary. We suggest using land use and the regional total water withdrawal to pre-filter data for further analysis. One can suspect that wells with the land use class ‘forests_natural_vegetation’ and 0 m³ regional water withdrawal are more likely to be undisturbed.

A benefit of the dataset is that 36 explanatory variables are provided, which originate from different geodata formats and resolutions. These variables can be used for clustering which may result in groups that resemble hydrological response units^98^ for groundwater. Such a clustering can potentially explain different groundwater behavior around the world, for example, as in Nolte *et al*.^59^ and Chávez *et al*.^51^.

GROW^56^ can additionally be used for model calibration and validation. We do not recommend aggregating the groundwater time series per raster cell for calibration or validation because 1) the aquifers might not be structurally connected, and 2) this creates time series that do not exist. For example, different trends and extreme points may get neutralized. The latter is especially probable as groundwater dynamics can vary greatly locally^51,59^. Additionally, 3) aggregated time series might have different temporal resolutions and lengths. Instead, comparing the local groundwater observations with modelled raster data has the potential to reveal how well a model can represent certain wells within that raster.

## Supplementary information

Supplementary Information for 'A Global-Scale Time Series Dataset for Groundwater Studies within the Earth System'

## Data Availability

The final GROW dataset^56^, containing an attributes table and a time series table, as well as a dataset documentation can be downloaded on Zenodo: 10.5281/zenodo.15149480 as either a CSV or parquet file. GROW^56^ itself is published under Creative Commons Attribution Non-Commercial ShareAlike 4.0 International License (CC-BY-NC-SA 4.0). An example of use that demonstrates how the data are subset and prepared is given on GitHub (https://github.com/EarthSystemModelling/GROW/blob/main/usage_example.py*)* and Zenodo (10.5281/zenodo.15149480).

## Code availability

The version of the code used for the preparation of the dataset^56^ is available from Zenodo (10.5281/zenodo.15149480) and can be viewed and downloaded on GitHub: https://github.com/EarthSystemModelling/GROW*)*. Moreover, a usage example is provided in both repositories.
