# Supplementary material for: A Global-Scale Time Series Dataset for Groundwater Studies within the Earth System
Source: Sci Data. 2026 Mar 9;13:401. doi: 10.1038/s41597-026-06966-1 (PMC12996427; doi:10.1038/s41597-026-06966-1)
Supplement: Supplementary file 1 — Supplementary Information for 'A Global-Scale Time Series Dataset for Groundwater Studies within the Earth System' [file 41597_2026_6966_MOESM1_ESM.pdf]

Supplementary Information for

A Global-Scale Time Series Dataset for Groundwater Studies within the Earth System

Annemarie Bäthge[1*], Claudia Ruz Vargas[2], Gunnar Lischeid[3,4], Raoul Collenteur[5], Mark Cuthbert[6], Jan Fleckenstein[7], Martina Flörke[8], Inge de Graaf[9], Sebastian Gnann[10], Andreas Hartmann[11], Xander Huggins[12-14], Nils Moosdorf[15,16], Yoshihide Wada[17], Thorsten Wagener[4], Robert Reinecke[1]

[1]Institute of Geography, Johannes Gutenberg-University, Mainz, Mainz, Germany
[2]International Groundwater Resources Assessment Centre (IGRAC), Delft, The Netherlands
[3]Research Area 4: 'Simulation & Data Science', Leibniz Centre for Agricultural Landscape Research, Müncheberg, Germany
[4]Institute of Environmental Science and Geography, University of Potsdam, Germany
[5]Department Water Resources and Drinking Water, Eawag, Dübendorf, Zürich, Switzerland
[6]School of Earth and Environmental Sciences, Cardiff University, UK
[7]Department of Hydrogeology, Helmholtz Center for Environmental Research, Leipzig, Germany
[8]Institute of Engineering Hydrology and Water Resources Management, Ruhr University Bochum, Bochum, Germany
[9]Earth Systems and Global Change group, Wageningen University and Research, Wageningen, the Netherlands
[10]Chair of Hydrology, University of Freiburg, Freiburg, Germany
[11]Institute of Groundwater Management, Dresden University of Technology
[12]Institute for Resources, Environment, and Sustainability, University of British Columbia, Vancouver, BC, Canada
[13]High Meadows Environmental Institute, Princeton University, Princeton, NJ, United States of America
[14]Stockholm Resilience Centre, Stockholm University, Stockholm, Sweden
[15]Leibniz Centre for Tropical Marine Research (ZMT), Bremen, Germany
[16]Kiel University, Kiel, Germany
[17]Biological and Environmental Science and Engineering Division, King Abdullah University of Science and Technology, Thuwal, Saudi Arabia
* Correspondence: a.baethge@uni-mainz.de

Content

In the supplements, additional figures regarding discarded or flagged groundwater time series and figures displaying the distribution of the rest of the Earth system variables are given. Additionally, the area-weighting to generate the distribution figures is described. A documentation to the GROW dataset can be found in the Readme file on Zenodo.

S1: PREPROCESSING OF GROUNDWATER TIME SERIES 4

TABLE S.1-1: VARIANCE DEVIATION (VARIANCE DIFFERENCE/VARIANCE OF UNPROCESSED TIME SERIES) OF TIME SERIES AFTER AGGREGATION. 4
TABLE S.1-2: DESCRIPTIVE STATISTICS SHOWING THE MEDIAN NUMBER OF VALUES AGGREGATED INTO EACH RECORD IN A TIME SERIES (DATA FLAG 'AGGREGATED_FROM_N_VALUES_MEDIAN'). 4
TABLE S.1-3: NUMBER OF FLAGGED TIME SERIES BEFORE PREPROCESSING (RAW DATA) AND AFTER EACH PREPROCESSING STEP IN WHICH DATA RECORDS WERE AGGREGATED OR REMOVED. 5
TABLE S.1-4: RESULTS OF SENSITIVITY ANALYSIS WITH THREE DIFFERENT THRESHOLD SETS WHICH DERIVE FROM THE TOTAL ALLOWED GAP FRACTION PER TIME SERIES (10%, 20%, 30%). 5
FIGURE S.1-1: EXAMPLES OF GROUNDWATER TIME SERIES THAT WERE DROPPED BECAUSE THE INDIVIDUAL GAP LENGTH BETWEEN THE TIME STEPS EXCEEDED THE THRESHOLD. 8
FIGURE S.1-2: EXAMPLES OF GROUNDWATER TIME SERIES THAT WERE DROPPED BECAUSE THE TOTAL GAP FRACTION OF THE TIME SERIES EXCEEDED THE THRESHOLD. 9
FIGURE S.1-3: EXAMPLES OF GROUNDWATER TIME SERIES IN THE FINAL GROW DATASET THAT WERE FLAGGED TO CONTAIN NEGATIVE GROUNDWATER DEPTH RECORDS (NEGATIVE RECORDS INDICATE THAT THE HYDRAULIC HEAD IS ABOVE GROUND). 10
FIGURE S.1-4: EXAMPLES OF GROUNDWATER TIME SERIES THAT WERE FLAGGED TO CONTAIN OUTLIERS AND/OR CHANGE POINTS (SPIKES OR BREAKS). 11

FIGURE S.1-5: EXAMPLES OF GROUNDWATER TIME SERIES THAT WERE FLAGGED TO CONTAIN PLATEAUS (UNINTERRUPTED SEQUENCES OF THE EXACT SAME VALUE)......12
FIGURE S1-6: NUMBER OF TIME SERIES PER COUNTRY, CATEGORIZED BY TEMPORAL RESOLUTION......13
FIGURE S.1-7: GAP FRACTION AND MEDIAN GAP LENGTH PER TIME SERIES WITH GAPS......14

S2: DISTRIBUTION OF EARTH SYSTEM VARIABLES IN GROW AND GLOBALLY 15

CALCULATION OF THE GLOBAL DISTRIBUTION/STATISTICS OF THE EARTH SYSTEM VARIABLES......15
TABLE S.2-1: OVERVIEW OF ALL ADDED EARTH SYSTEM ATTRIBUTES (NO TEMPORAL DIMENSION), THEIR DATA SOURCE AND CHARACTERISTICS.......16
TABLE S.2-2: OVERVIEW OF ALL ADDED TIME-VARYING EARTH SYSTEM VARIABLES, THEIR DATA SOURCE, AND CHARACTERISTICS.......18
FIGURE S.2-1: DISTRIBUTION OF GROUPED KOEPPEN-GEIGER CLIMATE CLASS IN THE GROW DATASET COMPARED WITH THE GLOBAL DISTRIBUTION.......20
FIGURE S.2-2: DISTRIBUTION OF GROUPED HYDROBELT CLASS IN THE GROW DATASET COMPARED WITH THE GLOBAL DISTRIBUTION.......21
FIGURE S.2-3: DISTRIBUTION OF GROUND ELEVATION IN THE GROW DATASET COMPARED WITH THE GLOBAL DISTRIBUTION...22
FIGURE S.2-4: DISTRIBUTION OF TOPOGRAPHIC SLOPE IN THE GROW DATASET COMPARED WITH THE GLOBAL DISTRIBUTION. 23
FIGURE S.2-5: DISTRIBUTION OF ROCK TYPE IN THE GROW DATASET COMPARED WITH THE GLOBAL DISTRIBUTION.......24
FIGURE S.2-6: DISTRIBUTION OF AQUIFER TYPE IN THE GROW DATASET COMPARED WITH THE GLOBAL DISTRIBUTION.......25
FIGURE S.2-7: DISTRIBUTION OF PERMEABILITY IN THE GROW DATASET COMPARED WITH THE GLOBAL DISTRIBUTION.......26
FIGURE S.2-8: DISTRIBUTION OF TOTAL POROSITY IN THE GROW DATASET COMPARED WITH THE GLOBAL DISTRIBUTION.......27
FIGURE S.2-9: DISTRIBUTION OF SOIL TEXTURE CLASS (TOPSOIL) IN THE GROW DATASET COMPARED WITH THE GLOBAL DISTRIBUTION.......28
FIGURE S.2-10: DISTRIBUTION OF SOIL TEXTURE CLASS (SUBSOIL) IN THE GROW DATASET COMPARED WITH THE GLOBAL DISTRIBUTION.......29
FIGURE S.2-11: DISTRIBUTION OF SATURATED HYDRAULIC CONDUCTIVITY (TOPSOIL) IN THE GROW DATASET COMPARED WITH THE GLOBAL DISTRIBUTION.......30
FIGURE S.2-12: DISTRIBUTION OF SATURATED HYDRAULIC CONDUCTIVITY (SUBSOIL) IN THE GROW DATASET COMPARED WITH THE GLOBAL DISTRIBUTION.......31
FIGURE S.2-13: DISTRIBUTION OF DISTANCE BETWEEN PERENNIAL STREAMS IN THE GROW DATASET COMPARED WITH THE GLOBAL DISTRIBUTION.......32
FIGURE S.2-14: DISTRIBUTION OF DRAINAGE DENSITY IN THE GROW DATASET COMPARED WITH THE GLOBAL DISTRIBUTION. 33
FIGURE S.2-15: DISTRIBUTION OF GLACIER COVER IN THE SURFACE CATCHMENT IN THE GROW DATASET COMPARED WITH THE GLOBAL DISTRIBUTION.......34
FIGURE S.2-16: DISTRIBUTION OF PERMAFROST COVER IN THE SURFACE CATCHMENT IN THE GROW DATASET COMPARED WITH THE GLOBAL DISTRIBUTION.......35
FIGURE S.2-17: DISTRIBUTION OF GROUNDWATER DEPENDENT ECOSYSTEMS IN THE GROW DATASET COMPARED WITH THE GLOBAL DISTRIBUTION.......36
FIGURE S.2-18: DISTRIBUTION OF MAIN LAND USE IN THE GROW DATASET COMPARED WITH THE GLOBAL DISTRIBUTION.......37
FIGURE S.2-19: DISTRIBUTION OF GROUNDWATERSCAPES IN THE GROW DATASET COMPARED WITH THE GLOBAL DISTRIBUTION.......38
FIGURE S.2-20: DISTRIBUTION OF DAILY PRECIPITATION FROM MSWEP IN THE GROW DATASET COMPARED WITH THE GLOBAL DISTRIBUTION.......39
FIGURE S.2-21: DISTRIBUTION OF MONTHLY PRECIPITATION FROM GPCC IN THE GROW DATASET COMPARED WITH THE GLOBAL DISTRIBUTION.......40
FIGURE S.2-22: DISTRIBUTION OF DAILY POTENTIAL EVAPOTRANSPIRATION FROM ERA5-LAND IN THE GROW DATASET COMPARED WITH THE GLOBAL DISTRIBUTION.......41
FIGURE S.2-23: DISTRIBUTION OF DAILY POTENTIAL EVAPOTRANSPIRATION FROM GLEAM IN THE GROW DATASET COMPARED WITH THE GLOBAL DISTRIBUTION.......42
FIGURE S.2-24: DISTRIBUTION OF DAILY ACTUAL EVAPOTRANSPIRATION FROM GLEAM IN THE GROW DATASET COMPARED WITH THE GLOBAL DISTRIBUTION.......43
FIGURE S.2-26: DISTRIBUTION OF DAILY INTERCEPTION IN THE GROW DATASET COMPARED WITH THE GLOBAL DISTRIBUTION.......44

FIGURE S.2-27: DISTRIBUTION OF DAILY AVERAGE AIR TEMPERATURE IN THE GROW DATASET COMPARED WITH THE GLOBAL DISTRIBUTION. *45*
FIGURE S.2-28: DISTRIBUTION OF DAILY AVERAGE SNOW DEPTH IN THE GROW DATASET COMPARED WITH THE GLOBAL DISTRIBUTION. *46*
FIGURE S.2-29: DISTRIBUTION OF DAILY AVERAGE NDVI IN THE GROW DATASET COMPARED WITH THE GLOBAL DISTRIBUTION. *47*
FIGURE S.2-30: DISTRIBUTION OF DAILY AVERAGE LEAF AREA INDEX OF LOW VEGETATION IN THE GROW DATASET COMPARED WITH THE GLOBAL DISTRIBUTION. *48*
FIGURE S.2-31: DISTRIBUTION OF DAILY AVERAGE LEAF AREA INDEX OF HIGH VEGETATION IN THE GROW DATASET COMPARED WITH THE GLOBAL DISTRIBUTION. *49*
FIGURE S.2-32: DISTRIBUTION OF ANNUAL TOTAL WATER WITHDRAWAL FOR INDUSTRIAL USE IN THE GROW DATASET COMPARED WITH THE GLOBAL DISTRIBUTION. *50*
FIGURE S.2-33: DISTRIBUTION OF ANNUAL TOTAL WATER WITHDRAWAL FOR DOMESTIC USE IN THE GROW DATASET COMPARED WITH THE GLOBAL DISTRIBUTION. *51*
FIGURE S.2-34: DISTRIBUTION OF ANNUAL URBAN AREA FRACTION IN THE GROW DATASET COMPARED WITH THE GLOBAL DISTRIBUTION. *52*
FIGURE S.2-35: DISTRIBUTION OF ANNUAL PASTURES FRACTION IN THE GROW DATASET COMPARED WITH THE GLOBAL DISTRIBUTION. *53*
FIGURE S.2-36: DISTRIBUTION OF ANNUAL RAINFED CROPLAND FRACTION IN THE GROW DATASET COMPARED WITH THE GLOBAL DISTRIBUTION. *54*
FIGURE S.2-37: DISTRIBUTION OF ANNUAL IRRIGATED CROPLAND FRACTION IN THE GROW DATASET COMPARED WITH THE GLOBAL DISTRIBUTION. *55*
FIGURE S.2-38: DISTRIBUTION OF ANNUAL FRACTION OF FORESTS AND NATURAL VEGETATION IN THE GROW DATASET COMPARED WITH THE GLOBAL DISTRIBUTION. *56*

REFERENCES **57**

S1: Preprocessing of groundwater time series

***Table S.1-1: Variance deviation (variance difference/variance of unprocessed time series) of time series after aggregation**. The number of time series whose variance was (not) altered after aggregation is presented in groups.*

Grouped variance deviation	Number of time series
Same variance (no aggregation/ no variance difference after aggregation)	130725 (61 %)
Deviation <= 1%	6829 (3 %)
Deviation >1% & <= 10%	15158 (7 %)
Deviation > 10% & <= 50%	30721 (14 %)
Deviation > 50% & <=100%	30992 (14 %)
Deviation > 100%	1103 (<1 %)

Table S.1-2: Descriptive statistics showing the median number of values aggregated into each record in a time series (data flag 'aggregated_from_n_values_median').

Median number of values that are aggregated to one record per time series	Daily data	Monthly data	Yearly data
Minimum	1	1	1
25th percentile	1	1	2
50th percentile	1	1	3
75th percentile	1	4	4
Maximum	96	31	23
Mean	2	3	4
Percentage of aggregated time series	66%	60%	39%

Table S.1-3: Number of flagged time series before preprocessing (raw data) and after each preprocessing step in which data records were aggregated or removed. *The steps in the brackets refer to the preprocessing step order in Table 2 in the manuscript.*

Number of time series with …	Raw data	After aggregation (step 4)	After gap control (step 5)
Some negative groundwater depths	3007 (1.4%)	2340 (1%)	1690 (0.8%)
Only negative groundwater depths	829 (0.4)	858 (0.4%)	964 (0.4%)
Outliers/change points	30888 (14%)	22678 (11%)	15177 (7%)
Plateaus	47016 (22%)	26714 (12%)	23176 (11%)
Autocorrelation	58008 (27%)	40145 (19%)	34964 (16%)
Trends	No trend: 58%<br>Decreasing: 28%<br>Increasing: 15%	No trend: 61%<br>Decreasing: 26%<br>Increasing: 13%	No trend: 65%<br>Decreasing: 23%<br>Increasing: 12%

Table S.1-4: Results of sensitivity analysis with three different threshold sets which derive from the total allowed gap fraction per time series (10%, 20%, 30%). *The number of time series might differ between the final GROW dataset as further time series were removed during the preprocessing of the attribute tables (i.e. duplicate removal). The entire data processing and the order of the data flag generation can be traced in the method section of the manuscript. The thresholds in the black-bordered column were used in the final dataset.*

	10%	20%	30%
Threshold settings in script	Temporal aggregation - Percentage of the time series that must be within a certain resolution to be aggregated to that resolution (daily and monthly data): 90%<br><br>Maximum allowed total gap percentage: 10%<br><br>Maximum allowed time interval between two records in days:<br>- daily data: 4<br>- monthly data: 62<br>- yearly data, 1-4 a: 366	Temporal aggregation - Percentage of the time series that must be within a certain resolution to be aggregated to that resolution (daily and monthly data): 80%<br><br>Maximum allowed total gap percentage: 20%<br><br>Maximum allowed time interval between two records in days:<br>- daily data: 7<br>- monthly data: 92<br>- yearly data, 1-4 a: 366	Temporal aggregation - Percentage of the time series that must be within a certain resolution to be aggregated to that resolution (daily and monthly data): 70%<br><br>Maximum allowed total gap percentage: 30%<br><br>Maximum allowed time interval between two records in days:<br>- daily data: 10<br>- monthly data: 123<br>- yearly data, 1-4 a: 366

	- yearly data, 5-9 a: 366<br>- yearly data, 10-14 a: 731<br>- yearly data, 15-19 a: 731<br>- yearly data, >20 a: 1096<br><br>Minimum length of plateaus to be flagged:<br>- daily data: 4 days<br>- monthly data: 2 months<br>- yearly data, 1-4 a: 2 years<br>- yearly data, 5-9 a: 2 years<br>- yearly data, 10-14 a: 2 years<br>- yearly data, 15-19 a: 2 years<br>- yearly data, >20 a: 3 years	- yearly data, 5-9 a: 731<br>- yearly data, 10-14 a: 1096<br>- yearly data, 15-19 a: 1461<br>- yearly data, >20 a: 1827<br><br>Minimum length of plateaus to be flagged:<br>- daily data: 7 days<br>- monthly data: 3 months<br>- yearly data, 1-4 a: 2 years<br>- yearly data, 5-9 a: 2 years<br>- yearly data, 10-14 a: 3 years<br>- yearly data, 15-19 a: 4 years<br>- yearly data, >20 a: 5 years	- yearly data, 5-9 a: 731<br>- yearly data, 10-14 a: 1461<br>- yearly data, 15-19 a: 1827<br>- yearly data, >20 a: 2557<br><br>Minimum length of plateaus to be flagged:<br>- daily data: 10 days<br>- monthly data: 5 months<br>- yearly data, 1-4 a: 2 years<br>- yearly data, 5-9 a: 2 years<br>- yearly data, 10-14 a: 4 years<br>- yearly data, 15-19 a: 5 years<br>- yearly data, >20 a: 7 years
**Number of time series after time series preprocessing**			
Total number of timeseries	215528	215323	215344
Number of daily timeseries	11475 (5%)<br>-66% aggregated with average median of 1.9 records per day-	12515 (6%)<br>-66 % aggregated with average median of 1.8 records per day-	13002 (6%)<br>-66 % aggregated with average median of 1.8 records per day-
Number of monthly timeseries	18754 (9%)<br>-60 % aggregated with average median of 3 records per month-	23095 (11%)<br>-63 % aggregated with average median of 1.9 records per month-	25619 (12%)<br>-65 % aggregated with average median of 1.7 records per month-
Number of yearly timeseries	185299 (86%)<br>-39 % aggregated with average median of 3.6 records per year-	179713 (83%)<br>-37 % aggregated with average median of 3.1 records per year-	176723 (82%)<br>-37 % aggregated with average median of 2.9 records per year-
**Time series trimming due to gap check**			
Number of trimmed time series	102283 (47%)	103139 (48%)	103400 (48%)
Average reduction in data amount	18%	19%	18%

Average and median reduction in time span	Mean: 27%<br>Median: 8%	Mean: 27%<br>Median: 8%	Mean: 27%<br>Median: 8%
**Flags**			
Number of time series with gaps	14682 (7%)	21593 (10%)	26339 (12%)
Average gap percentage of time series with gaps	2.5%	5.1%	7.6%
Number of time series with autocorrelation	34964 (16%)	39892 (19%)	41977 (19%)
Number of time series with some or only negative groundwater depth records	Some: 0.8%<br>All: 0.4%	Some: 0.8%<br>All: 0.4%	Some: 0.8%<br>All: 0.4%
Number of time series with outliers/change points	15177 (7%)	15907 (7%)	16436 (8%)
Number of time series with plateaus	23176 (11%)	10998 (5%)	6886 (3%)
Trends	No trend: 65%<br>Decreasing: 23%<br>Increasing: 12%	No trend: 65%<br>Decreasing: 23%<br>Increasing: 12%	No trend: 64%<br>Decreasing: 23%<br>Increasing: 12%

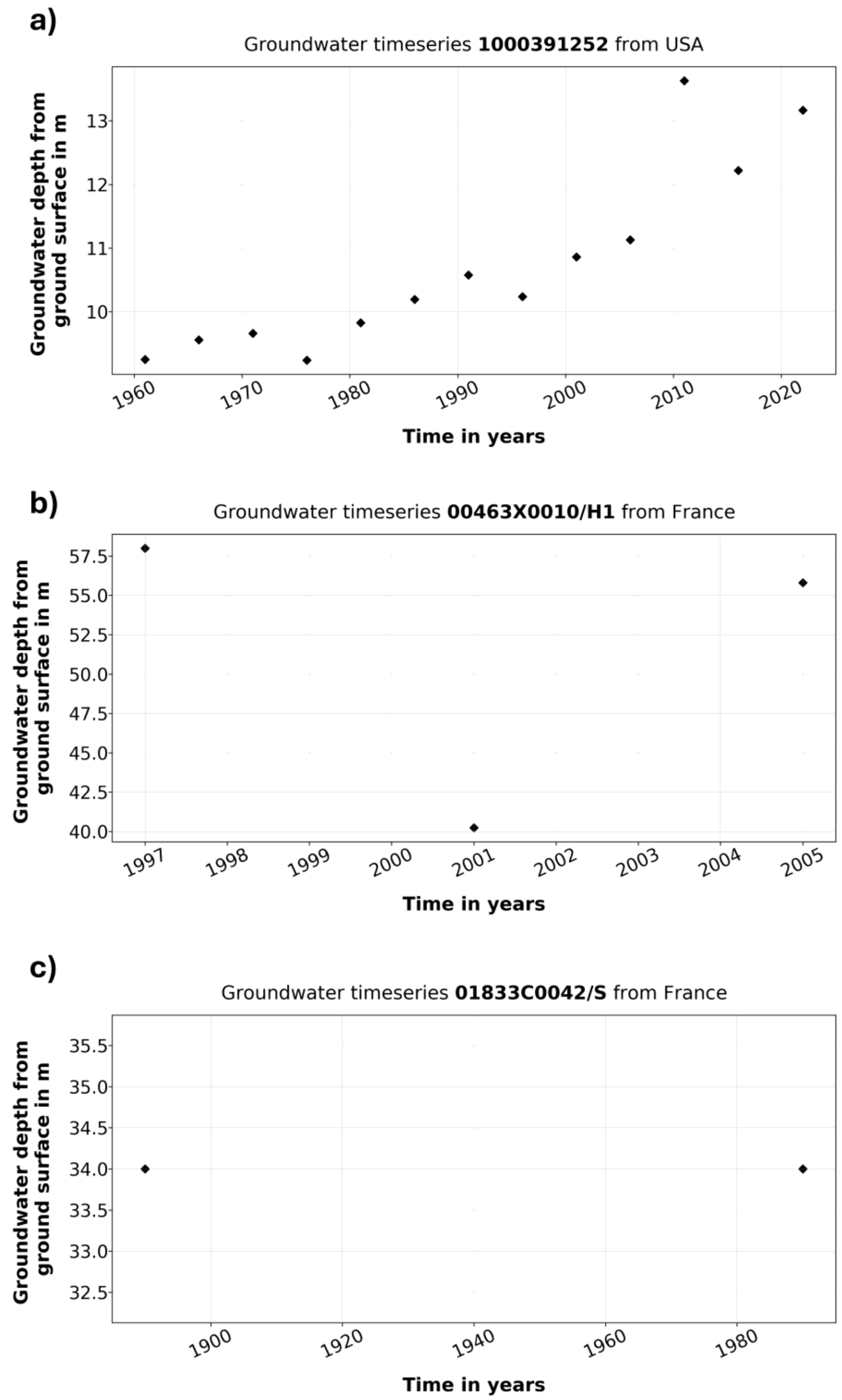

Figure S.1-1: Examples of groundwater time series that were dropped because the individual gap length between the time steps exceeded the threshold. *Groundwater time series are shown in the state after the aggregation processing step (see in manuscript method section – step 5.b). Plot a) shows a yearly time series with gap lengths of mostly 4 years between the records. Plot b) displays a yearly time series with gaps of 3 years, and the yearly time series in plot c) has one large gap of 99 years. The bolt texts in the plot titles are the original ID's from the groundwater data source.*

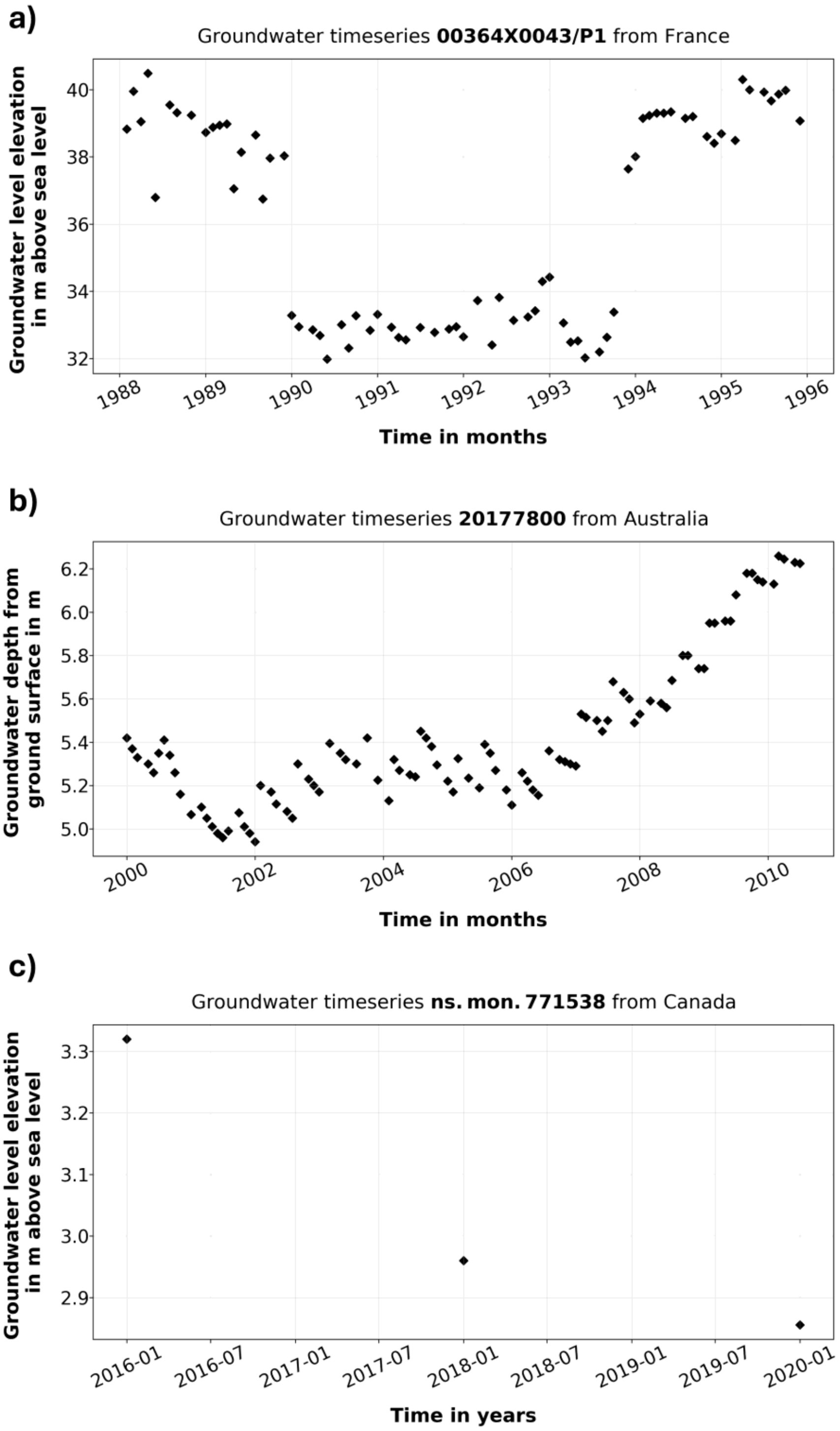

Figure S.1-2: Examples of groundwater time series that were dropped because the total gap fraction of the time series exceeded the threshold. *Groundwater time series are shown in the state after the extraction of sequences in which the time step intervals do not exceed the individual gap length threshold (see in manuscript method section – step 5.a). Plot a) shows a monthly time series with a total gap fraction of 25%. The monthly time series in plot b) has a total gap fraction of 24% and the yearly time series in c) has a total gap fraction of 40%. The bolt texts in the plot titles are the original ID's from the groundwater data source.*

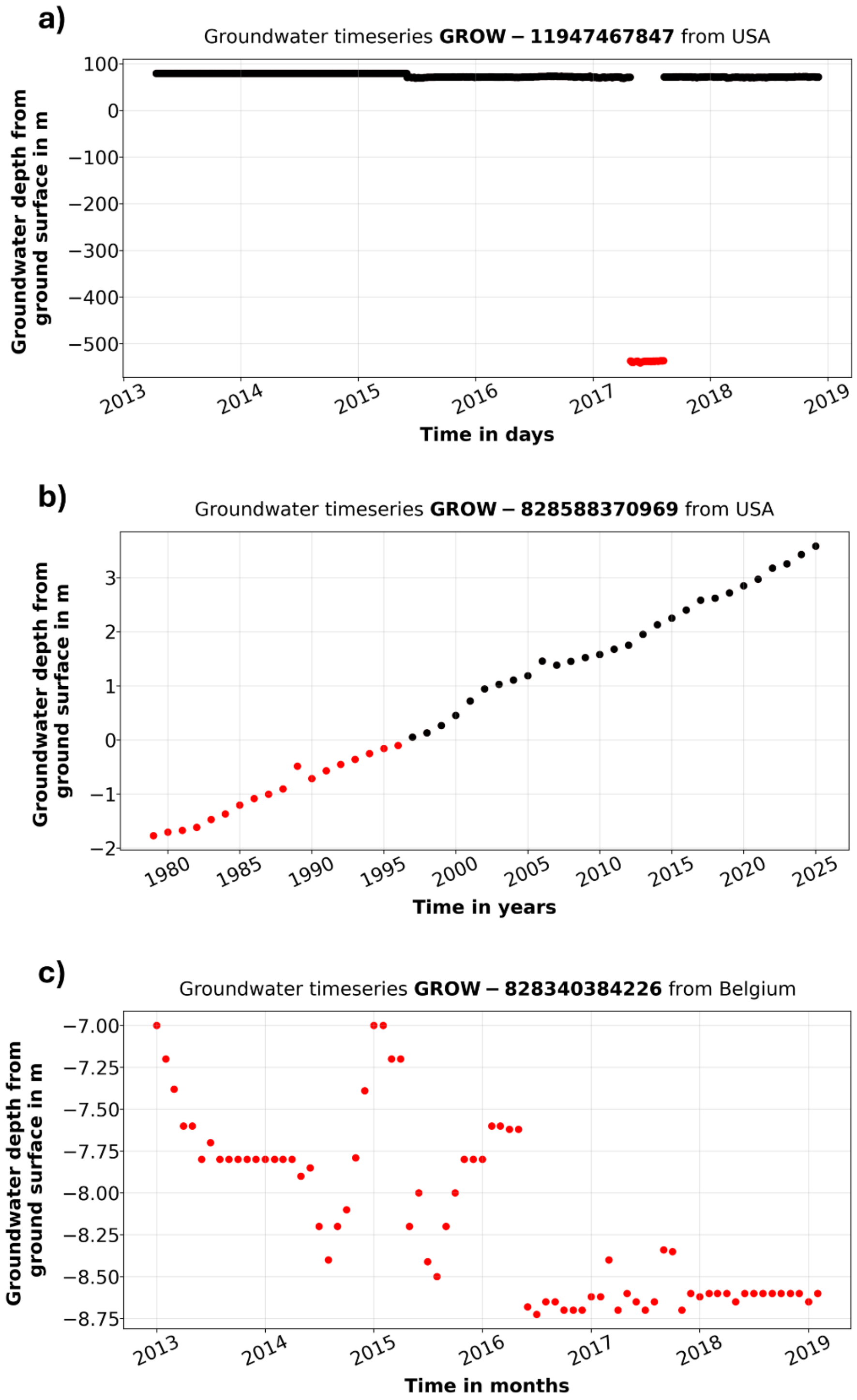

Figure S.1-3: Examples of groundwater time series in the final GROW dataset that were flagged to contain negative groundwater depth records (negative records indicate that the hydraulic head is above ground). *Plots a) and b) show time series with positive (black dots) and negative records (red dots). They are flagged to contain "Some" negative records (data flag 'negative_signs_wtd). Plot c) shows a time series with only negative records (flagged with 'All' in 'negative_signs_wtd). The bolt texts in the plot titles are the GROW-ID's in the final dataset.*

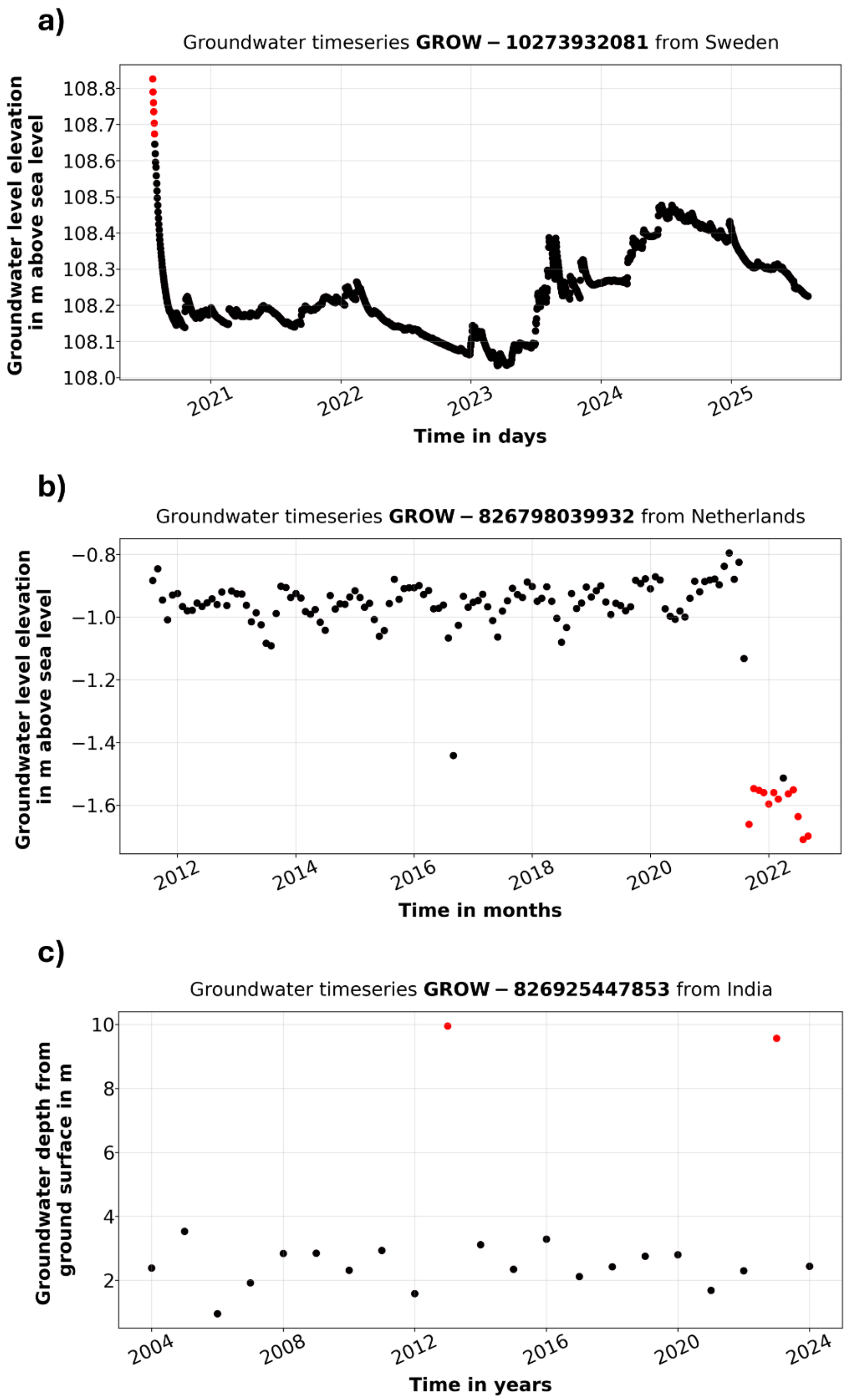

Figure S.1-4: Examples of groundwater time series that were flagged to contain outliers and/or change points (spikes or breaks). *The plots show a a) daily, b) monthly, and c) yearly groundwater time series in GROW in which the DBSCAN algorithm[1] detected outliers and/or change points. The DBSCAN algorithm is explained in more detail in the manuscript's method section (step 8). The detected outliers are marked in red. The bolt texts in the plot titles are the GROW-ID's in the final dataset.*

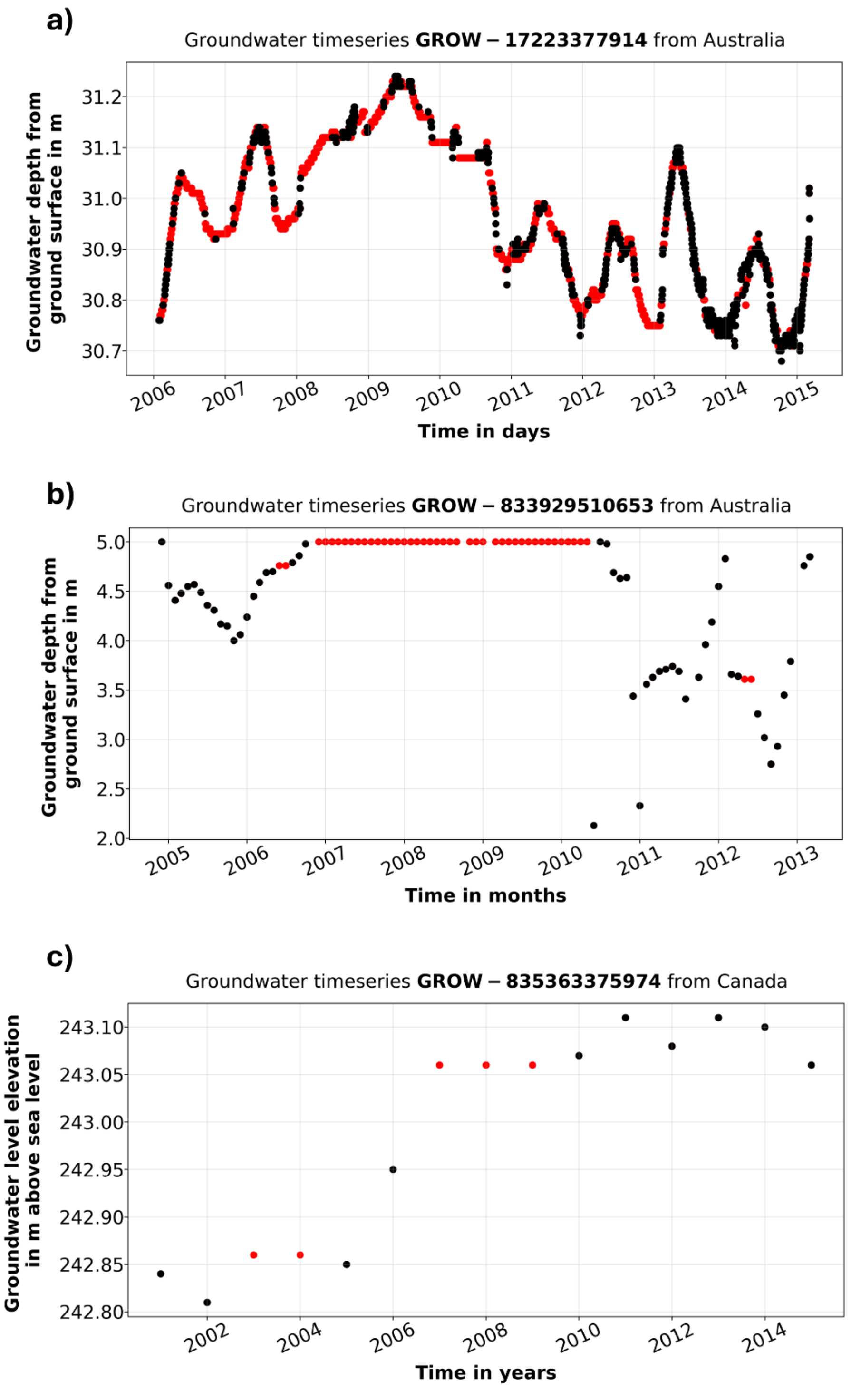

Figure S.1-5: Examples of groundwater time series that were flagged to contain plateaus (uninterrupted sequences of the exact same value). *The plots show a a) daily, b) monthly, and c) yearly groundwater time series in GROW that contain uninterrupted sequences of the exact same value that exceeded determined thresholds (see manuscript method section step 9). The plateaus are marked in red. The bolt texts in the plot titles are the GROW-ID's in the final dataset.*

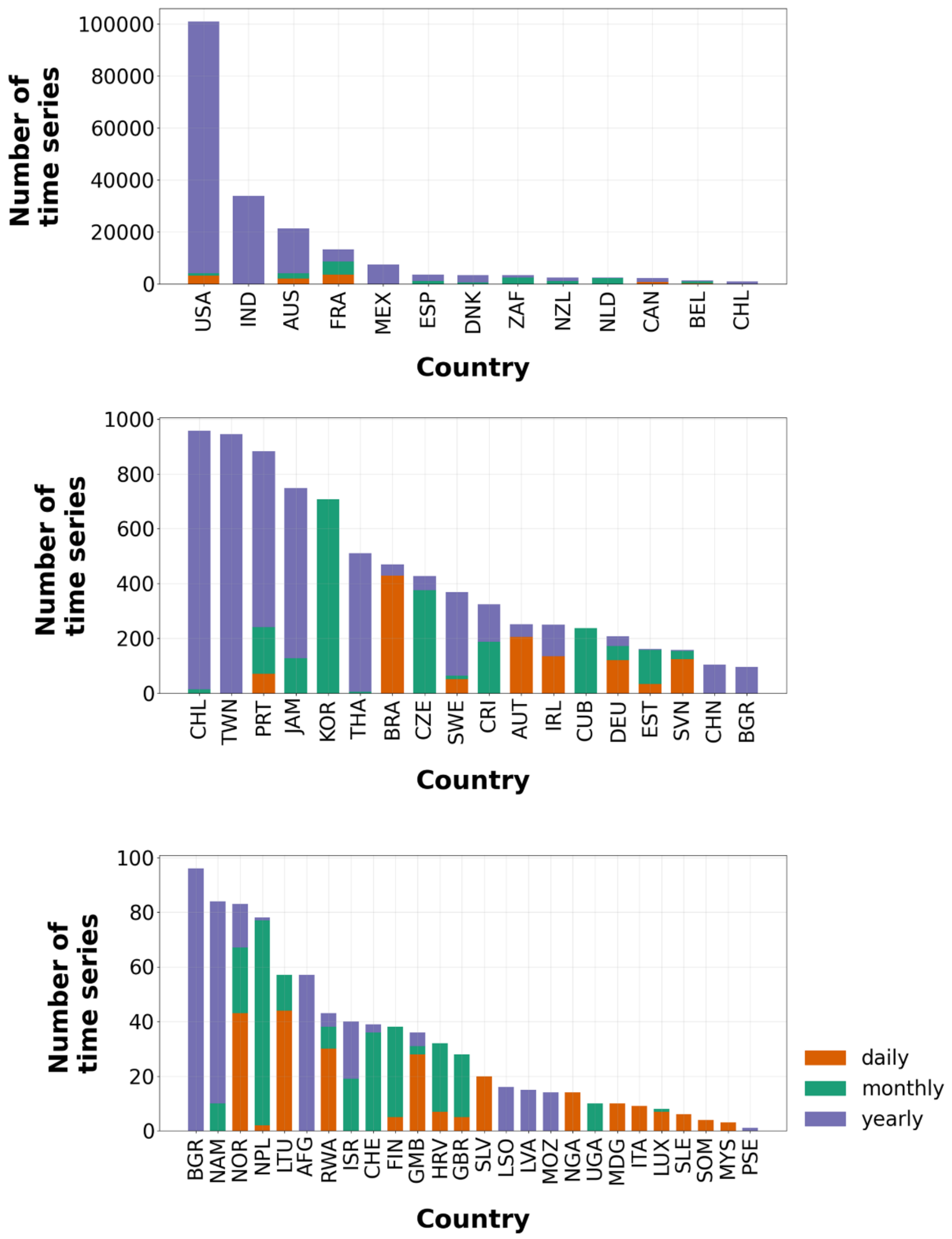

Figure S1-6: Number of time series per country, categorized by temporal resolution. *The country names are abbreviated according to ISO 3166-1 alpha-3.*

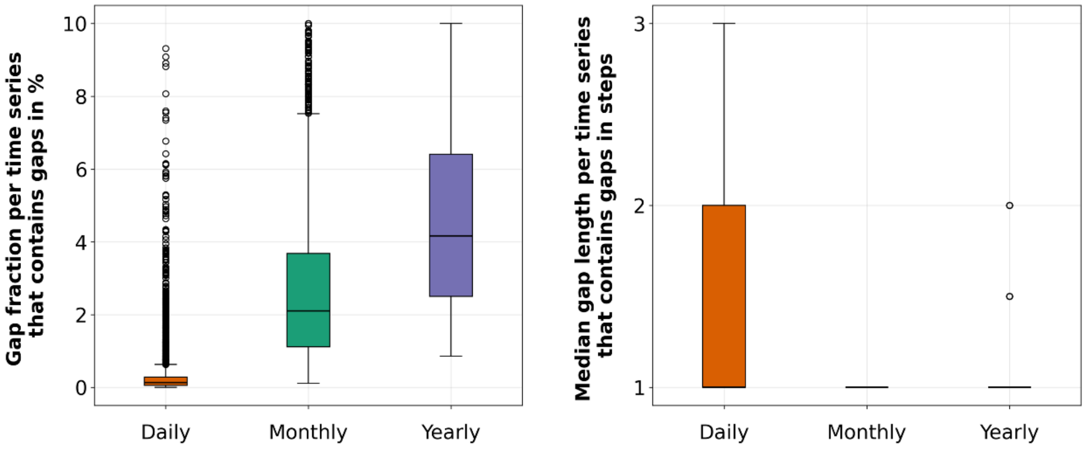

Figure S.1-7: Gap fraction and median gap length per time series with gaps. *Boxplots of median gap fraction and gap length per time series, categorized by temporal resolution. Only time series in GROW that contain gaps are included (14,682). Box plots show the median of a distribution as a black line inside the box. The upper and lower edges of the box are determined by the 25th and 75th percentiles (interquartile range). Whiskers indicate the farthest data point within 1.5 times the interquartile range. Outliers outside this range are displayed as dots.*

S2: Distribution of Earth system variables in GROW and globally

Calculation of the global distribution/statistics of the Earth system variables

To calculate the global distribution/statistics of the Earth system variables, all data products that were available as vector data were transformed into a 0.05° raster to enable comparability. For that purpose, the QGIS 3.28.14 function *"Rasterize"* was utilized.

To account for the area distortion of the WGS 84 coordinate system, area weighting was applied in the following way:

- The area weighting bases on the calculated pixel areas of a global 0.05° WGS 84 grid that were used as scaling factors. The algorithm described in Santini et al.[2] was used for the area calculation. They span from 0.014 km² (latitude of -90°/90°) to 30.773 km² (latitude of 0°).
- To every pixel of a global raster, the according area scaling factor was assigned. To do so, the global raster of the Earth system variable and the raster with the scaling was loaded with the python package xarray. The raster with the scaling was reprojected to the projection, resolution and extent of the global raster with the function *rio.reproject_match* of the python package rioxarray. Both rasters were linked along the latitude and longitude coordinates.
- For the global distribution of the categoric Earth system variables the scaling factors per pixel were summed for every class and divided by the total sum of all scaling factors to end up with a percentage. Latter was used to generate the stacked bar charts.
- For the calculation of the area-weighted median per numeric Earth system variable, the pixel values of the global raster were grouped by exact same value and written to a numpy array (python package numpy). The number of times a value was written to the array depended on the scaling. The scaling factors per pixel were summed for every occurring value. Then, the scaling sum was rounded to a whole number. Scaling sums below 1 were set to 1. The according value was added to the array as many times as the rounded scaling sum was large. The median value of that array was taken as global median. As the array grows very large beyond the actual number of pixels per data product, the data amount was limited for the global statistics to guarantee computational feasibility. In case of daily resolution data, only the year 2014 was utilized. It should be noted that this opens the possibility of the global minimum and maximum value to be larger or smaller than the GROW minimum and maximum. High resolution static data like ground elevation (3 arc seconds), topographic slope (7.5 arc seconds), saturated hydraulic conductivity (8.2 arc seconds) and soil texture class (8.2 arc seconds) were aggregated to 30 arc seconds, beforehand.

Table S.2-1: Overview of all added Earth system attributes (no temporal dimension), their data source and characteristics. *Record coverage refers to the percentage of wells in GROW's attributes table that contain a value for that variable. The source type "Observation-based" also includes remote sensing products.*

Earth system variable	Column name in GROW	Earth system component	Data source (Source type)	Data access	Spatial resolution	Original Unit	Unit in GROW	Record coverage
Koeppen-Geiger classification	koeppen_geiger_class	Atmosphere	CHELSA v2.1 - kg0[3] (Downscaled Reanalysis)	https://doi.org/10.16904/ENVIDAT.228.V2.1	30 arc sec	/	/	100%
Hydrobelt	hydrobelt_class	Atmosphere & Hydrosphere	Meybeck et al. 2013[4] (Model + Observation-based)	https://doi.org/10.1594/PANGAEA.806957	*polygons*	/	/	90%
Ground elevation	ground_elevation_merit_m_asl	Geosphere	MERIT DEM v1.0.3[5] (Observation-based)	http://hydro.iis.u-tokyo.ac.jp/~yamadai/MERIT_DEM/	3 arc sec	m	m	100%
Topographic slope	topographic_slope_degree	Geosphere	Geomorpho90m[6] (Observation-based)	https://hs.pangaea.de/Maps/DEM_Geomorpho90m/dtm_slope_merit.dem_m_250m_s0..0cm_2018_v1.0.tif	7.5 arc sec	°	°	>99%
Rock type	rock_type_0-100_m_class	Geosphere	GLiM v1[7] (Observation-based)	https://doi.pangaea.de/10.1594/PANGAEA.788537	*0.5°*	/	/	96%
Aquifer type	aquifer_type_class	Geosphere	WHYMAP WOKAM[8]; GLiM[9] (Observation-based)	http://doi.org/10.25928/b2.21_sfkq-r406; https://doi.pangaea.de/10.1594/PANGAEA.788537	*Polygons; 0.5°*	/	/	96%
Permeability for 0-100 m depth	permeability_0-100_m_m-2	Geosphere	GLHYMPS2.0[10] (Observation-based)	https://doi.org/10.5683/SP2/TTJNIU	*polygons*	m²	m²	>99%
Total porosity for 0-100 m depth	total_porosity_0-100_m_fraction	Geosphere	GLHYMPS[11] (Observation-based)	https://doi.org/10.5683/SP2/DLGXYO	*polygons*	/	/	>99%
Soil texture class in topsoil (0-30 cm)	soil_texture_0-30_cm_class	Geosphere	HiHydroSoil v2.0[12] (Model)	https://www.futurewater.eu/projects/hihydrosoil-v2-0-global-maps-of-soil-hydraulic-properties-at-250m-resolution/	250 m	/	/	>99%
Soil texture class in subsoil (30-200 cm)	soil_texture_30-200_cm_class	Geosphere	HiHydroSoil v2.0[12] (Model)	https://www.futurewater.eu/projects/hihydrosoil-v2-0-global-	250 m	/	/	66%

				maps-of-soil-hydraulic-properties-at-250m-resolution/				
Saturated hydraulic conductivity of topsoil (0-30 cm)	soil_saturated_conductivity_0-30_cm_cm_d-1	Geosphere	HiHydroSoil v2.0[12] (Model)	https://www.futurewater.eu/projects/hihydrosoil-v2-0-global-maps-of-soil-hydraulic-properties-at-250m-resolution/	250 m	cm/ day	cm/ day	>99%
Saturated hydraulic conductivity of subsoil (30-200 cm)	soil_saturated_conductivity_30-200_cm_cm_d-1	Geosphere	HiHydroSoil v2.0[12] (Model)	https://www.futurewater.eu/projects/hihydrosoil-v2-0-global-maps-of-soil-hydraulic-properties-at-250m-resolution/	250 m	cm/ day	cm/ day	>99%
Distance between perennial streams	distance_perennial_streams_m	Hydrosphere	Cuthbert, Gleeson et al.[13] - 0.1 cubic metres per second flow threshold (Observation-based)	https://figshare.com/articles/dataset/Global_water_table_ratio_and_groundwater_response_time_raster_data/7393304?file=28402347	1 km	m	m	>99%
Drainage density	drainage_density_m-1	Hydrosphere	HydroRivers[14]; BasinATLAS Level 9[15] (Observation-based)	https://www.hydrosheds.org/products/hydrorivers; https://www.hydrosheds.org/hydroatlas	*polygons + polylines*	m^{-1}	m^{-1}	>99%
Glacier cover in surface catchment	glacier_cover_fraction	Cyrosphere	BasinATLAS Level 9[15] (Observation-based)	https://www.hydrosheds.org/hydroatlas	*polygons*	/	/	>99%
Permafrost cover in surface catchment	permafrost_cover_fraction	Cyrosphere	BasinATLAS Level 9[15] (Model)	https://www.hydrosheds.org/hydroatlas	*polygons*	/	/	>99%
Average days per year with snow cover	days_with_snow_cover_average_days_year-1	Cyrosphere	ERA5-Land[16,17] (Model)	https://cds.climate.copernicus.eu/doi/10.24381/cds.e9c9c792	0.08°	m	days/ year	96%
Groundwater dependent ecosystems	groundwater_dependent_ecosystems_class	Biosphere	Huggins et al.[18] (Multi-source)	https://doi.org/10.5683/SP3/P3OU3A	30 arc sec	/	/	100%
Main land use	main_landuse_class	Anthropo-sphere	Volkholz & Ostberg 2024[19] (Model)	https://data.isimip.org/10.48364/ISIMIP.571261.3	0.5°	/	/	97%
Groundwaterscapes	groundwaterscapes_ID_class	Integrates multiple components	Huggins et al.[20] (Multi-source)	https://doi.org/10.5683/SP3/MFYCWV	5 arc min	/	/	93%

Table S.2-2: Overview of all added time-varying Earth system variables, their data source, and characteristics. *Record coverage refers to the percentage of records in GROW's time series table that contain a value for that variable. The source type "Observation-based" also includes remote sensing products.*

Earth system variable	Column name in GROW	Earth system component	Data source (Source type)	Data access	Spatial resolution	Temporal resolution	Temporal coverage	Original Unit	Unit in GROW	Record coverage
Precipitation	precipitation_mswep_mm_year-1	Atmosphere	MSWEP V2[21] (Multi-source)	https://drive.google.com/drive/folders/1Kok05OPVESTpyyan7NafR-2WwuSJ4TO9	0.25°	daily	1979-2025	mm/day	mm/year	98%
Precipitation	precipitation_gpcc_mm_year-1	Atmosphere	GPCC[22] (Observation-based)	https://opendata.dwd.de/climate_environment/GPCC/full_data_monthly_v2022/025/	0.25°	monthly	1891-2020	mm/month	mm/year	82%
Potential Evapo-transpiration	potential_evapotranspiration_era5_mm_year-1	Atmosphere	ERA5-Land[16,17] (Model)	https://cds.climate.copernicus.eu/doi/10.24381/cds.e9c9c792	0.08°	daily	1950-2025	mm/day	mm/year	97%
Potential Evapo-transpiration	potential_evapotranspiration_gleam_mm_year-1	Atmosphere	GLEAM4[23] (Model)	https://www.gleam.eu/	0.25°	daily	1980-2023	mm/day	mm/year	88%
Actual Evapo-transpiration	actual_evapotranspiration_mm_year-1	Atmosphere	GLEAM4[23] (Model)	https://www.gleam.eu/	0.25°	daily	1980-2023	mm/day	mm/year	88%
Interception loss	interception_mm_year-1	Biosphere	GLEAM4[23] (Model)	https://www.gleam.eu/	0.25°	daily	1980-2023	mm/day	mm/year	88%
Air temperature	air_temperature_°C	Atmosphere	ERA5-Land[16,17] (Reanalysis)	https://cds.climate.copernicus.eu/doi/10.24381/cds.e9c9c792	0.08°	daily	1950-2025	K	°C	97%
Snow depth	snow_depth_m	Cyrosphere	ERA5-Land[16,17] (Model)	https://cds.climate.copernicus.eu/doi/10.24381/cds.e9c9c792	0.08°	daily	1950-2025	m	m	97%
Days per year with snow cover	days_with_snow_cover_days_year-1	Cyrosphere	ERA5-Land[16,17] (Model)	https://cds.climate.copernicus.eu/doi/10.24381/cds.e9c9c792	0.08°	yearly	1950-2025	m	days/year	97%
NDVI	ndvi_ratio	Biosphere	1981-2013: AVHRR NDVI[24]; 2014-2024: VIIRS NDVI[25] (Observation-based)	https://www.ncei.noaa.gov/data/land-normalized-difference-vegetation-index/access/	0.05°	daily	1981-2025	/	/	93%
Leaf area index of low vegetation	lai_low_vegetation_ratio	Biosphere	ERA5-Land[16,17] (Model)	https://cds.climate.copernicus.eu/doi/10.24381/cds.e9c9c792	0.08°	daily	1950-2025	/	/	97%

Leaf area index of high vegetation	lai_high_vegetation_ ratio	Biosphere	ERA5-Land[16,17] (Model)	https://cds.climate.copernicus.eu/doi/10.24381/cds.e9c9c792	0.08°	daily	1950-2025	/	/	97%
Total water withdrawal for industrial use	withdrawal_industrial _m3_year-1	Anthropos-phere	Wada et al.[26] - indww_histsoc_annual (Model)	https://data.isimip.org/10.48364/ISIMIP.228996	0.5°	yearly	1901 - 2021	m³/ year	m³/ year	87%
Total water withdrawal for domestic use	withdrawal_domestic_ m3_year-1	Anthropo-sphere	Wada et al.[26] - domww_histsoc_annual (Model)	https://data.isimip.org/10.48364/ISIMIP.228996	0.5°	yearly	1901 - 2021	m³/ year	m³/ year	87%
Fraction of urban areas	urban_area_fraction	Anthropo-sphere	Volkholz & Ostberg[19] - landuse-urbanareas_histsoc_ annual (Model)	https://data.isimip.org/10.48364/ISIMIP.571261.3	0.5°	yearly	1901 - 2021	/	/	87%
Fraction of pastures	pastures_fraction	Anthropo-sphere	Volkholz & Ostberg[19] - landuse-totals_histsoc_annual (Model)	https://data.isimip.org/10.48364/ISIMIP.571261.3	0.5°	yearly	1901 - 2021	/	/	87%
Fraction of rainfed cropland	cropland_rainfed_ fraction	Anthropo-sphere	Volkholz & Ostberg[19] - landuse-totals_histsoc_annual (Model)	https://data.isimip.org/10.48364/ISIMIP.571261.3	0.5°	yearly	1901 - 2021	/	/	87%
Fraction of irrigated cropland	cropland_irrigated_ fraction	Anthropo-sphere	Volkholz & Ostberg[19] - landuse-totals_histsoc_annual (Model)	https://data.isimip.org/10.48364/ISIMIP.571261.3	0.5°	yearly	1901 -2021	/	/	87%
Fraction of forests and natural vegetation	forests_natural_ vegetation_fraction	Anthropo-sphere	Volkholz & Ostberg[19] - landuse-totals_histsoc_annual (Model)	https://data.isimip.org/10.48364/ISIMIP.571261.3	0.5°	yearly	1901 -2021	/	/	87%

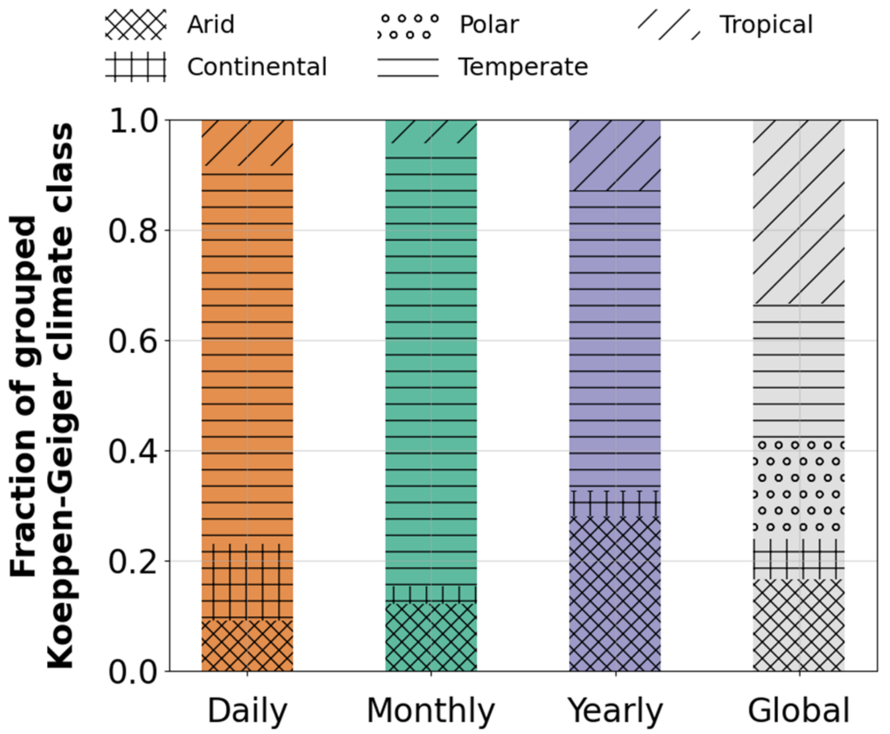

Fraction of grouped Koeppen-Geiger climate class in GROW and globally

	Daily	Monthly	Yearly	Global*
**Temperate**	0.69	0.80	0.55	0.25
**Arid**	0.09	0.12	0.28	0.17
**Continental**	0.14	0.03	0.05	0.07
**Tropical**	0.08	0.04	0.13	0.33
**Polar**	<0.01	<0.01	<0.01	0.18

Figure S.2-1: Distribution of grouped Koeppen-Geiger climate class in the GROW dataset compared with the global distribution. *This stacked bar chart is showing the distribution of the Koeppen-Geiger classification derived from CHELSA v2.1 (kg0)[3] in GROW classified by temporal resolution of the time series in comparison to the global fractions of the variable. For this display, the classification was grouped according to the main climate classes (A-Tropical, B-Dry, C-Temperate, D-Continental, E-Polar). *The global distribution is derived from all pixel values in the respective raster data with area-weighting to correct for the area distortion of the WGS 84 coordinate system.*

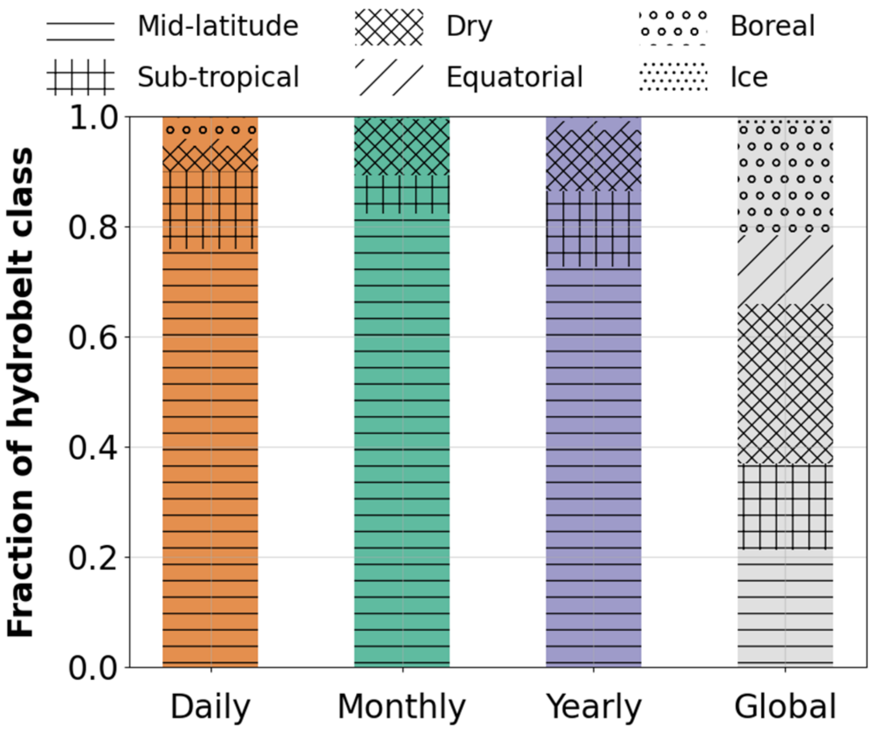

Fraction of Hydrobelt class in GROW and globally

	Daily	Monthly	Yearly	Global*
**Mid Latitude**	0.76	0.82	0.73	0.21
**Sub Tropical**	0.14	0.07	0.14	0.16
**Dry**	0.05	0.10	0.11	0.29
**Equatorial**	0.01	< 0.01	0.02	0.13
**Boreal**	0.04	< 0.01	0.01	0.20
**Ice**	0	0	0	0.02

Figure S.2-2: Distribution of grouped Hydrobelt class in the GROW dataset compared with the global distribution. *This stacked bar chart is showing the distribution of the Hydrobelt classification derived from Meybeck et al.*[4] *in GROW classified by temporal resolution of the time series in comparison to the global fractions of the variable. The classification was grouped to 5 classes (based on Table 1. in Meybeck et al.*[4]*) which are characterized by average annual temperature and runoff. To calculate the global distribution of the Hydrobelt classes, the polygon data was transformed into a 0.05° raster. *The global distribution is derived from all pixel values in the respective raster data with area-weighting to correct for the area distortion of the WGS 84 coordinate system.*

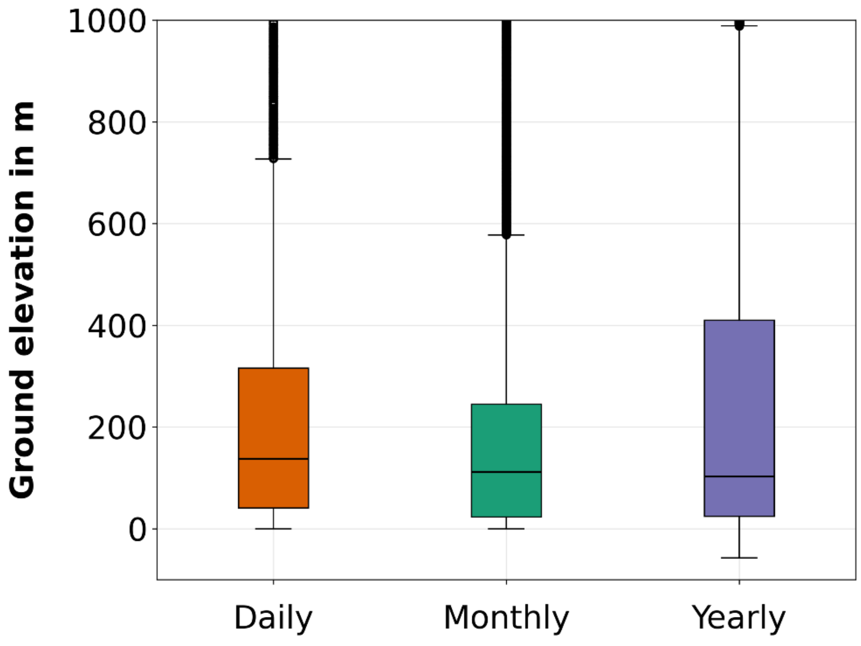

Descriptive statistics of ground elevation in m in GROW and globally

	Daily	Monthly	Yearly	Global
**min**	0	0	-58	- 154
**median**	137	112	103	366*
**max**	2771	2387	3847	8451

Figure S.2-3: Distribution of ground elevation in the GROW dataset compared with the global distribution. *This overview is showing a boxplot of the ground elevation derived from the MERIT DEM[5] in GROW classified by temporal resolution of the time series. Box plots show the median of a distribution as a black line inside the box. The upper and lower edges of the box are determined by the 25th and 75th percentiles (interquartile range). Whiskers indicate the farthest data point within 1.5 times the interquartile range. Outliers outside this range are displayed as dots. For readability, not all outliers are shown; thus, the tables below show the minimum, median, and maximum value of the ground elevation in GROW for every temporal resolution in comparison to the global statistics. *The median of the global distribution is derived from all pixel values in the respective raster data with area-weighting to correct for the area distortion of the WGS 84 coordinate system. To enable computational feasibility, the global raster was resampled to a spatial resolution of 30 arc seconds.*

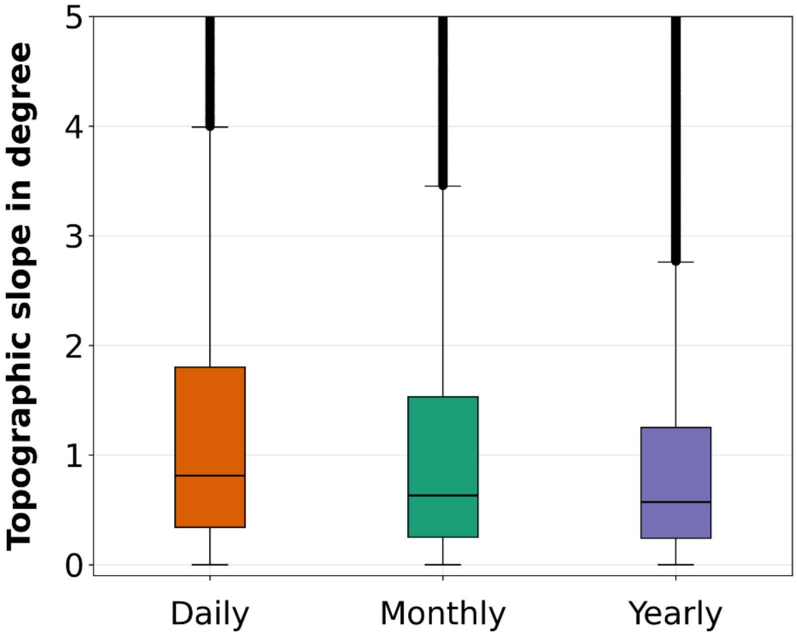

Descriptive statistics of topographic slope in degrees in GROW and globally

	Daily	Monthly	Yearly	Global
**min**	0	0	0	0
**median**	0.81	0.63	0.57	1.45*
**max**	41.59	30.82	43.43	71.91

Figure S.2-4: Distribution of topographic slope in the GROW dataset compared with the global distribution. *This overview is showing a boxplot of the topographic slope derived from* Geomorpho90m[6] *in GROW classified by temporal resolution of the time series. Box plots show the median of a distribution as a black line inside the box. The upper and lower edges of the box are determined by the 25th and 75th percentiles (interquartile range). Whiskers indicate the farthest data point within 1.5 times the interquartile range. Outliers outside this range are displayed as dots. For readability, not all outliers are shown; thus, the tables below show the minimum, median, and maximum value of the topographic slope in GROW for every temporal resolution in comparison to the global statistics. *The median of the global distribution is derived from all pixel values in the respective raster data with area-weighting to correct for the area distortion of the WGS 84 coordinate system. To enable computational feasibility, the global raster was resampled to a spatial resolution of 30 arc seconds.*

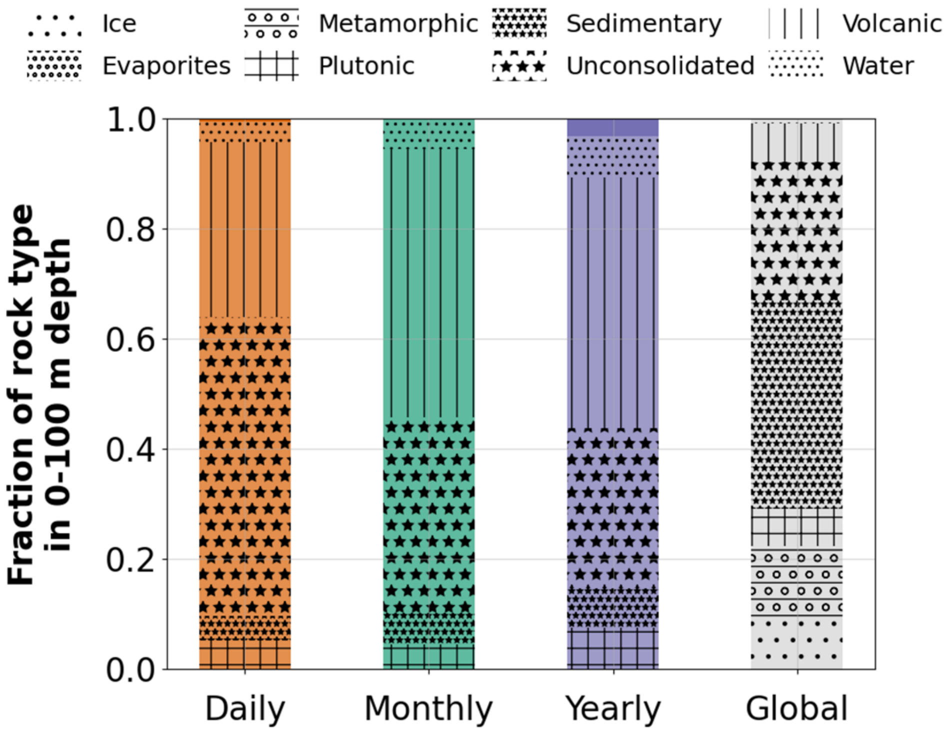

Fraction of grouped rock type in 0-100 m depth in GROW and globally

	Daily	Monthly	Yearly	Global*
**Ice**	0	0	0	0.09
**Evaporites**	0	<0.01	<0.01	<0.01
**Metamorphic**	0.05	0.04	0.07	0.13
**Plutonic**	0.05	0.06	0.07	0.07
**Sedimentary**	0.54	0.36	0.29	0.38
**Un-consolidated**	0.32	0.49	0.46	0.25
**Volcanic**	0.04	0.06	0.07	0.07
**Water**	<0.01	<0.01	0.03	<0.01

Figure S.2-5: Distribution of rock type in the GROW dataset compared with the global distribution. *This stacked bar chart is showing the distribution of the rock type in 0-100 m depth derived from the GLiM[7] in GROW classified by temporal resolution of the time series in comparison to the global fractions of the variable. For this display, all volcanic, plutonic and sedimentary rock types were grouped into one class. *The global distribution is derived from all pixel values in the respective raster data with area-weighting to correct for the area distortion of the WGS 84 coordinate system.*

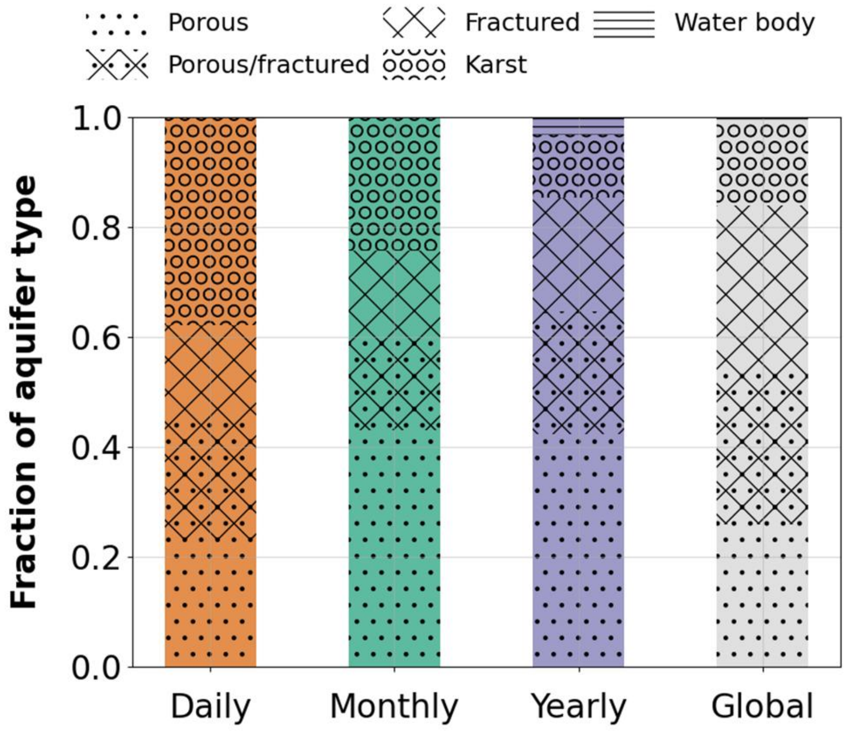

Fraction of aquifer type in GROW and globally

	Daily	Monthly	Yearly	Global*
**Porous**	0.23	0.43	0.42	0.26
**Porous/ fractured**	0.23	0.15	0.21	0.28
**Fractured**	0.16	0.18	0.22	0.30
**Karst**	0.38	0.24	0.11	0.15
**Water body**	<0.01	<0.01	0.03	0.01

Figure S.2-6: Distribution of aquifer type in the GROW dataset compared with the global distribution. *This stacked bar chart is showing the distribution of the aquifer type derived from WHYMAP WOKAM[8] and GLiM[7] in GROW classified by temporal resolution of the time series in comparison to the global fractions of the variable. To calculate the global distribution, the polygon data (WHYMAP WOKAM) were transformed into a 0.05° raster. *The global distribution is derived from all pixel values in the respective raster data with area-weighting to correct for the area distortion of the WGS 84 coordinate system.*

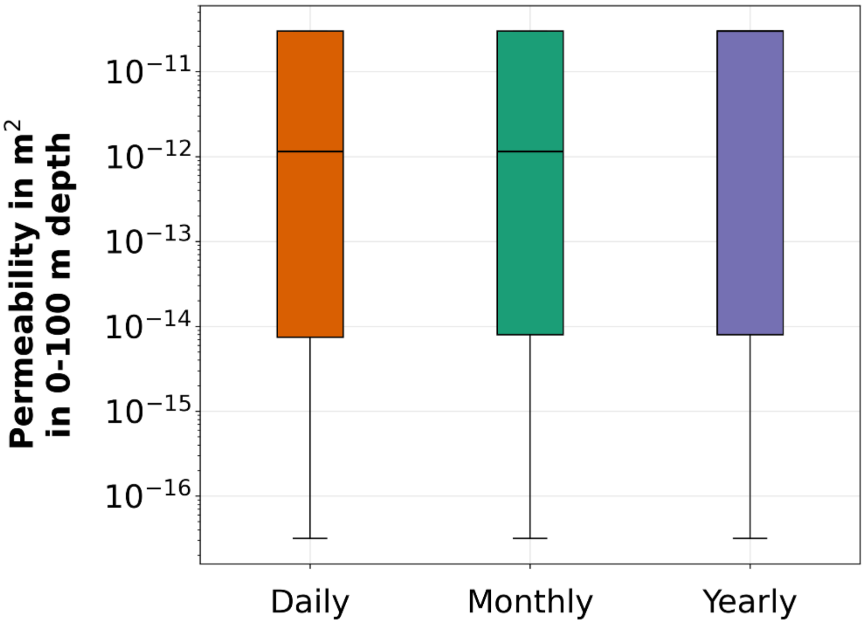

Descriptive statistics of permeability (0-100 m depth) in m² in GROW and globally

	Daily	Monthly	Yearly	Global
**min**	3.16e-17	3.16e-17	3.16e-17	3.16e-17
**median**	1.15e-12	1.15e-12	3.02e-11	3.16e-13*
**max**	3.02e-11	3.02e-11	3.02e-11	3.02e-11

Figure S.2-7: Distribution of permeability in the GROW dataset compared with the global distribution. *This overview is showing a boxplot of the permeability in 0-100 m depth derived from GLHYMPS2.0[10] in GROW classified by temporal resolution of the time series. Box plots show the median of a distribution as a black line inside the box. The upper and lower edges of the box are determined by the 25th and 75th percentiles (interquartile range). Whiskers indicate the farthest data point within 1.5 times the interquartile range. Outliers outside this range are displayed as dots. The tables below show the minimum, median, and maximum value of the permeability in GROW for every temporal resolution in comparison to the global statistics. To calculate the global distribution, the polygon data were transformed into a 0.05° raster. *The median of the global distribution is derived from all pixel values in the respective raster data with area-weighting to correct for the area distortion of the WGS 84 coordinate system.*

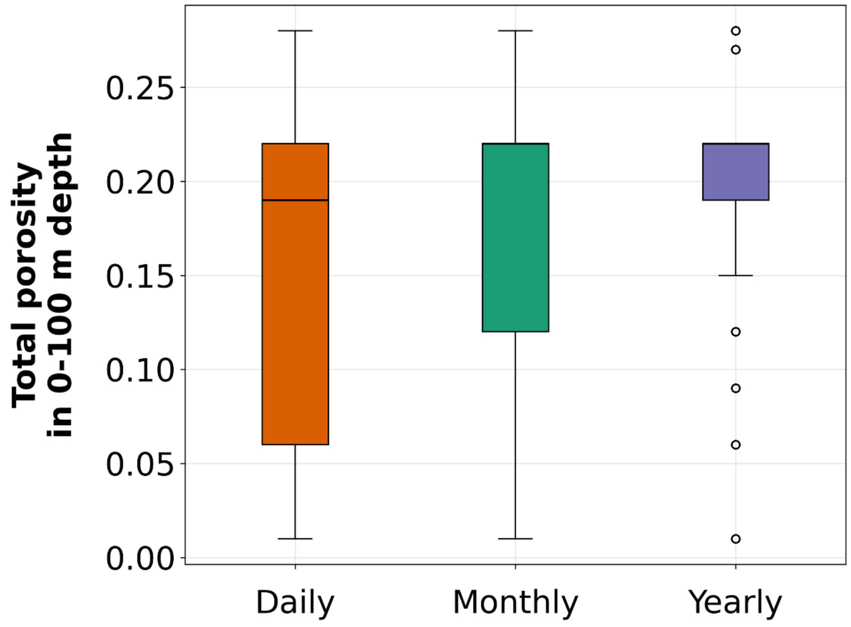

Descriptive statistics of total porosity (0-100 m depth) in GROW and globally

	Daily	Monthly	Yearly	Global
**min**	0.01	0.01	0.01	10e-5
**median**	0.19	0.22	0.22	0.19*
**max**	0.28	0.28	0.28	0.28

Figure S.2-8: Distribution of total porosity in the GROW dataset compared with the global distribution. *This overview is showing a boxplot of the total porosity in 0-100 m depth derived from GLHYMPS[11] in GROW classified by temporal resolution of the time series. Box plots show the median of a distribution as a black line inside the box. The upper and lower edges of the box are determined by the 25th and 75th percentiles (interquartile range). Whiskers indicate the farthest data point within 1.5 times the interquartile range. Outliers outside this range are displayed as dots. The tables below show the minimum, median, and maximum value of the total porosity in GROW for every temporal resolution in comparison to the global statistics. To calculate the global distribution, the polygon data were transformed into a 0.05° raster. *The median of the global distribution is derived from all pixel values in the respective raster data with area-weighting to correct for the area distortion of the WGS 84 coordinate system.*

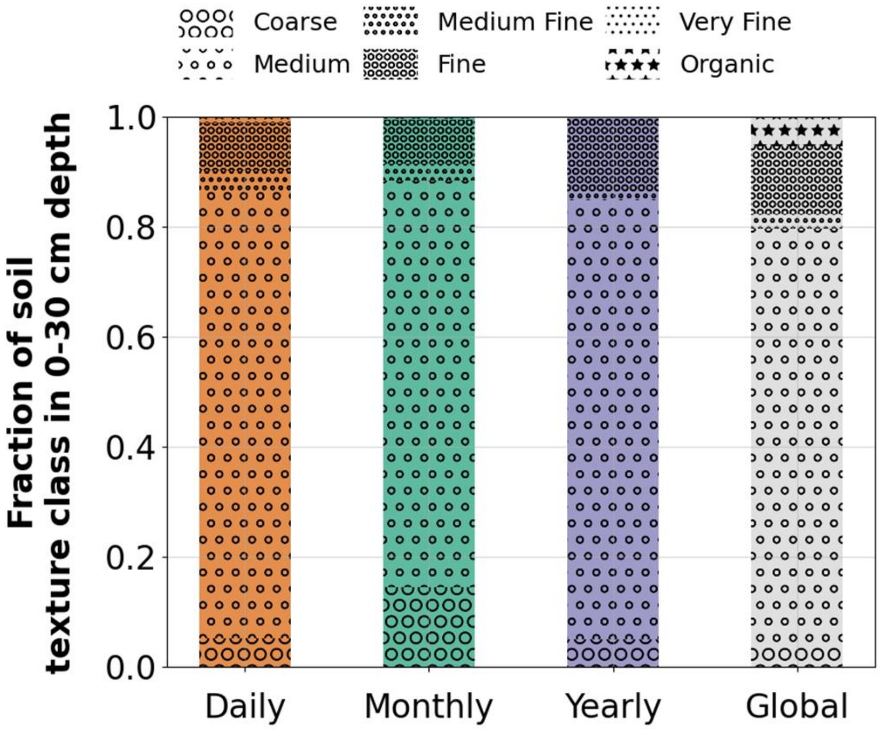

Fraction of soil texture class in 0-30 cm depth (topsoil) in GROW and globally

	Daily	Monthly	Yearly	Global*
**Coarse**	0.05	0.14	0.05	0.04
**Medium**	0.81	0.74	0.8	0.76
**Medium Fine**	0.04	0.03	0.02	0.03
**Fine**	0.09	0.08	0.13	0.12
**Very Fine**	<0.01	<0.01	<0.01	<0.01
**Organic**	0.01	<0.01	<0.01	0.05

Figure S.2-9: Distribution of soil texture class (topsoil) in the GROW dataset compared with the global distribution. *This stacked bar chart is showing the distribution of the soil texture class (0-30 cm depth) derived from HiHydroSoil[12] in GROW classified by temporal resolution of the time series in comparison to the global fractions of the variable. *The global distribution is derived from all pixel values in the respective raster data with area-weighting to correct for the area distortion of the WGS 84 coordinate system. To enable computational feasibility, the global raster was resampled to a spatial resolution of 30 arc seconds.*

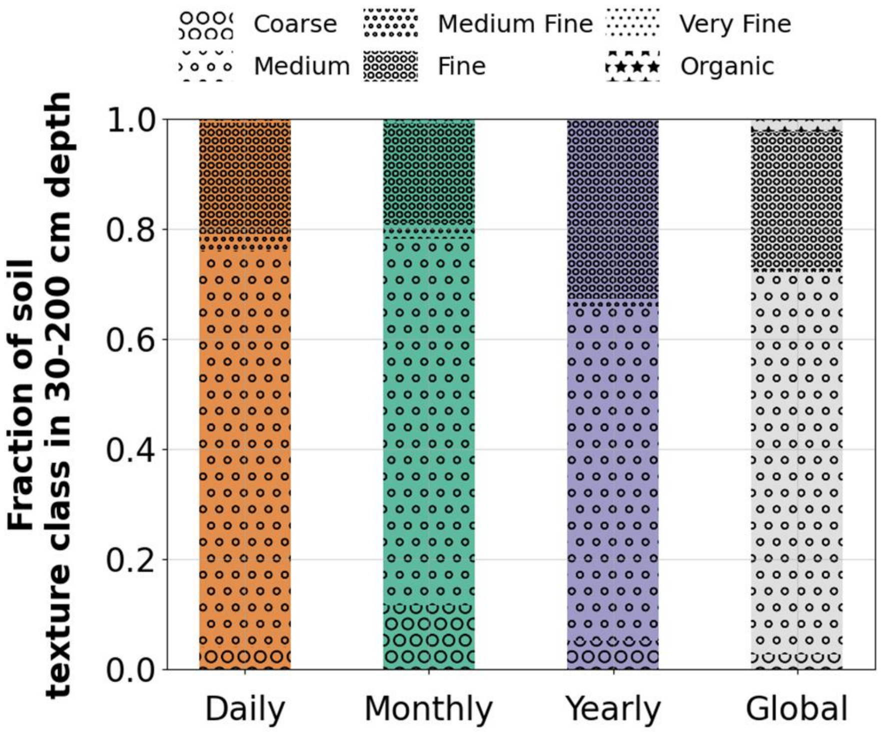

Fraction of soil texture class in 30-200 cm depth (subsoil) in GROW and globally

	Daily	Monthly	Yearly	Global*
**Coarse**	0.04	0.12	0.05	0.03
**Medium**	0.72	0.67	0.61	0.70
**Medium Fine**	0.03	0.03	0.01	<0.01
**Fine**	0.20	0.18	0.32	0.25
**Very Fine**	<0.01	0.01	<0.01	<0.01
**Organic**	<0.01	<0.01	<0.01	0.02

Figure S.2-10: Distribution of soil texture class (subsoil) in the GROW dataset compared with the global distribution. *This stacked bar chart is showing the distribution of the soil texture class (30-200 cm depth) derived from HiHydroSoil[12] in GROW classified by temporal resolution of the time series in comparison to the global fractions of the variable. *The global distribution is derived from all pixel values in the respective raster data with area-weighting to correct for the area distortion of the WGS 84 coordinate system. To enable computational feasibility, the global raster was resampled to a spatial resolution of 30 arc seconds.*

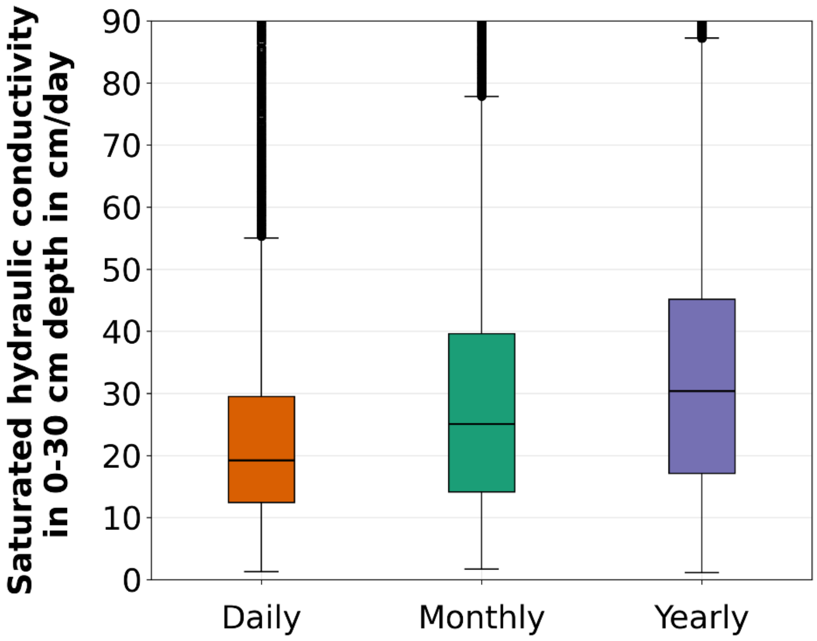

Descriptive statistics of saturated hydraulic conductivity in 0-30 cm depth (topsoil) in cm/day in GROW and globally

	Daily	Monthly	Yearly	Global
**min**	1.3	1.7	1.2	0
**median**	19.2	25.1	30.3	17.1*
**max**	1440.2	1440.2	1440.2	2961.5

Figure S.2-11: Distribution of saturated hydraulic conductivity (topsoil) in the GROW dataset compared with the global distribution. *This overview is showing a boxplot of the saturated hydraulic conductivity in (0 - 30 cm depth) derived from HiHydroSoil[12] in GROW classified by temporal resolution of the time series. Box plots show the median of a distribution as a black line inside the box. The upper and lower edges of the box are determined by the 25th and 75th percentiles (interquartile range). Whiskers indicate the farthest data point within 1.5 times the interquartile range. Outliers outside this range are displayed as dots. For readability, not all outliers are shown; thus, the tables below show the minimum, median, and maximum value of the saturated hydraulic conductivity in GROW for every temporal resolution in comparison to the global statistics. *The median of the global distribution is derived from all pixel values in the respective raster data with area-weighting to correct for the area distortion of the WGS 84 coordinate system. To enable computational feasibility, the global raster was resampled to a spatial resolution of 30 arc seconds.*

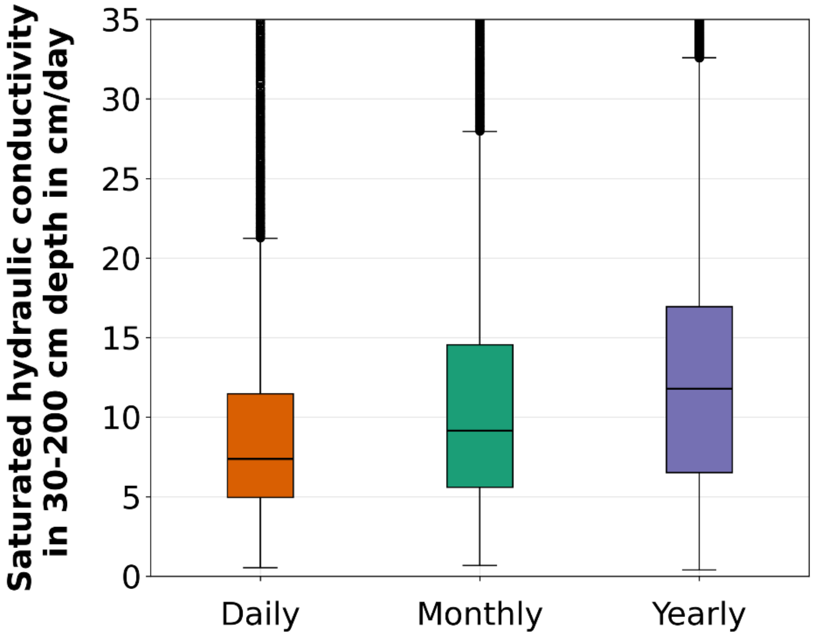

Descriptive statistics of saturated hydraulic conductivity in 30-200 cm depth (subsoil) in cm/day GROW and globally

	Daily	Monthly	Yearly	Global
**min**	0.5	0.7	0.4	0
**median**	7.4	9.2	11.8	8.5*
**max**	570.1	570.1	570.1	1504.6

Figure S.2-12: Distribution of saturated hydraulic conductivity (subsoil) in the GROW dataset compared with the global distribution. *This overview is showing a boxplot of the saturated hydraulic conductivity in (30 -200 cm depth) derived from HiHydroSoil*[12] *in GROW classified by temporal resolution of the time series. Box plots show the median of a distribution as a black line inside the box. The upper and lower edges of the box are determined by the 25th and 75th percentiles (interquartile range). Whiskers indicate the farthest data point within 1.5 times the interquartile range. Outliers outside this range are displayed as dots. For readability, not all outliers are shown; thus, the tables below show the minimum, median, and maximum value of the saturated hydraulic conductivity in GROW for every temporal resolution in comparison to the global statistics. *The median of the global distribution is derived from all pixel values in the respective raster data with area-weighting to correct for the area distortion of the WGS 84 coordinate system. To enable computational feasibility, the global raster was resampled to a spatial resolution of 30 arc seconds.*

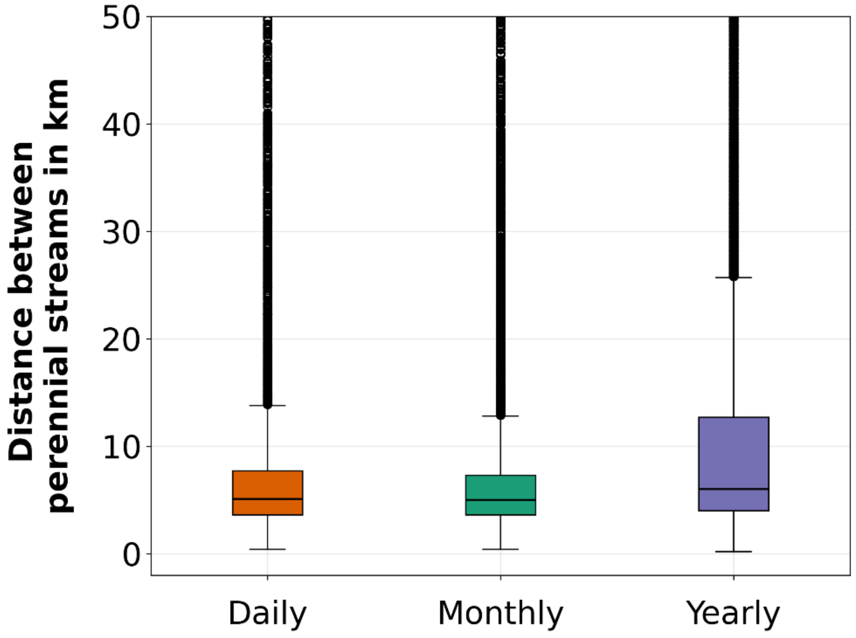

Descriptive statistics of distance between perennial streams in km in GROW and globally

	Daily	Monthly	Yearly	Global
**min**	0.4	0.4	0.2	0
**median**	5.1	5	6	7.9*
**max**	1215.1	1096	1215.5	1960.9

Figure S.2-13: Distribution of distance between perennial streams in the GROW dataset compared with the global distribution. *This overview is showing a boxplot of the distance between perennial streams derived from Cuthbert et al.*[27] *in GROW classified by temporal resolution of the time series. Box plots show the median of a distribution as a black line inside the box. The upper and lower edges of the box are determined by the 25th and 75th percentiles (interquartile range). Whiskers indicate the farthest data point within 1.5 times the interquartile range. Outliers outside this range are displayed as dots. For readability, not all outliers are shown; thus, the tables below show the minimum, median, and maximum value of the distance between perennial streams in GROW for every temporal resolution in comparison to the global statistics. *The median of the global distribution is derived from all pixel values in the respective raster data with area-weighting to correct for the area distortion of the WGS 84 coordinate system.*

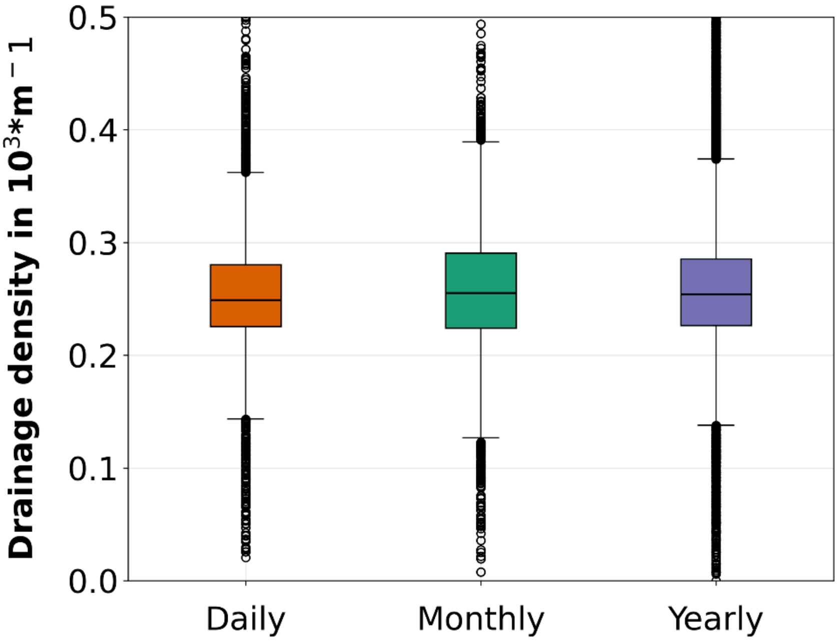

Descriptive statistics of drainage density in 10^3*m^{-1} in GROW and globally

	Daily	Monthly	Yearly	Global
**min**	2.1e-2	7.8e-3	6.2e-7	4.1e-9
**median**	0.25	0.25	0.25	0.25*
**max**	1.45	1.02	2.33	5.74

Figure S.2-14: Distribution of Drainage density in the GROW dataset compared with the global distribution. *This overview is showing a boxplot of the Drainage density derived from HydroRivers*[14] *and BasinATLAS Level 9*[15] *in GROW classified by temporal resolution of the time series. Box plots show the median of a distribution as a black line inside the box. The upper and lower edges of the box are determined by the 25th and 75th percentiles (interquartile range). Whiskers indicate the farthest data point within 1.5 times the interquartile range. Outliers outside this range are displayed as dots. For readability, not all outliers are shown; thus, the tables below show the minimum, median, and maximum value of the Drainage density in GROW for every temporal resolution in comparison to the global statistics. To calculate the global distribution, the polygon data were transformed into a 0.05° raster. *The median of the global distribution is derived from all pixel values in the respective raster data with area-weighting to correct for the area distortion of the WGS 84 coordinate system.*

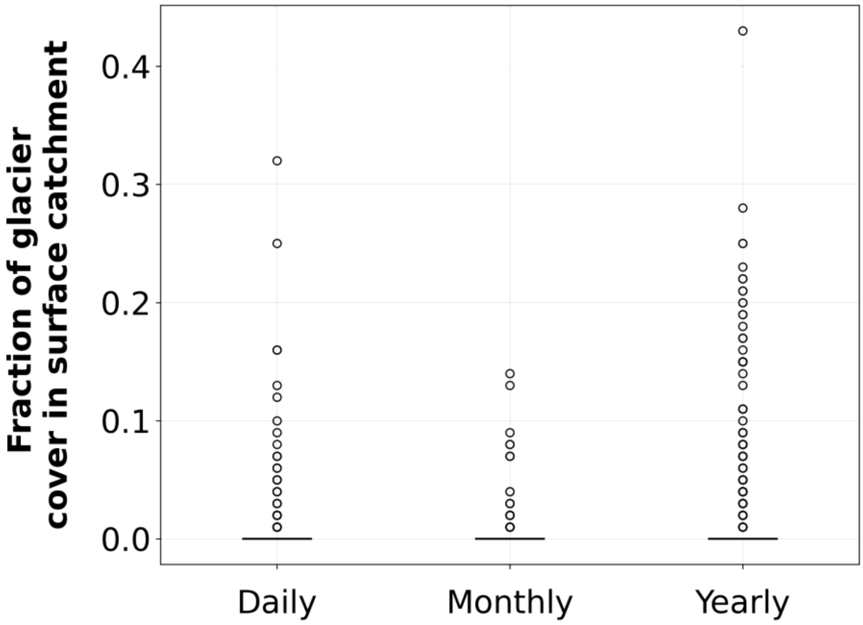

Descriptive statistics of glacier cover fraction in the surface catchment in which the well is located in GROW and globally

	Daily	Monthly	Yearly	Global
**min**	0	0	0	0
**median**	0	0	0	0*
**max**	0.32	0.14	0.43	1

Figure S.2-15: Distribution of glacier cover in the surface catchment in the GROW dataset compared with the global distribution. *This overview is showing a boxplot of the glacier cover in the surface catchments in which the wells are located derived from BasinATLAS Level 9*[15] in *GROW classified by temporal resolution of the time series. Box plots show the median of a distribution as a black line inside the box. The upper and lower edges of the box are determined by the 25th and 75th percentiles (interquartile range). Whiskers indicate the farthest data point within 1.5 times the interquartile range. Outliers outside this range are displayed as dots. The tables below show the minimum, median, and maximum value of the glacier cover in GROW for every temporal resolution in comparison to the global statistics. To calculate the global distribution, the polygon data were transformed into a 0.05° raster. *The median of the global distribution is derived from all pixel values in the respective raster data with area-weighting to correct for the area distortion of the WGS 84 coordinate system.*

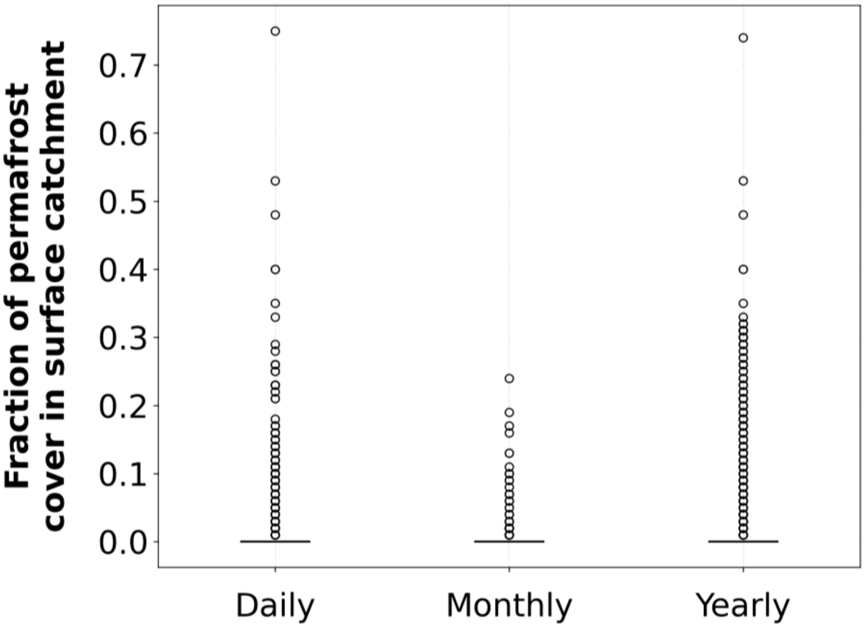

Descriptive statistics of the surface catchment's permafrost cover in GROW and globally

	Daily	Monthly	Yearly	Global
**min**	0	0	0	0
**median**	0	0	0	0*
**max**	0.75	0.24	0.74	1

Figure S.2-16: Distribution of permafrost cover in the surface catchment in the GROW dataset compared with the global distribution. *This overview is showing a boxplot of the permafrost cover in the surface catchments in which the wells are located derived from BasinATLAS Level 9*[15] in *GROW classified by temporal resolution of the time series. Box plots show the median of a distribution as a black line inside the box. The upper and lower edges of the box are determined by the 25th and 75th percentiles (interquartile range). Whiskers indicate the farthest data point within 1.5 times the interquartile range. Outliers outside this range are displayed as dots. The tables below show the minimum, median, and maximum value of the permafrost cover in GROW for every temporal resolution in comparison to the global statistics. To calculate the global distribution, the polygon data were transformed into a 0.05° raster. *The median of the global distribution is derived from all pixel values in the respective raster data with area-weighting to correct for the area distortion of the WGS 84 coordinate system.*

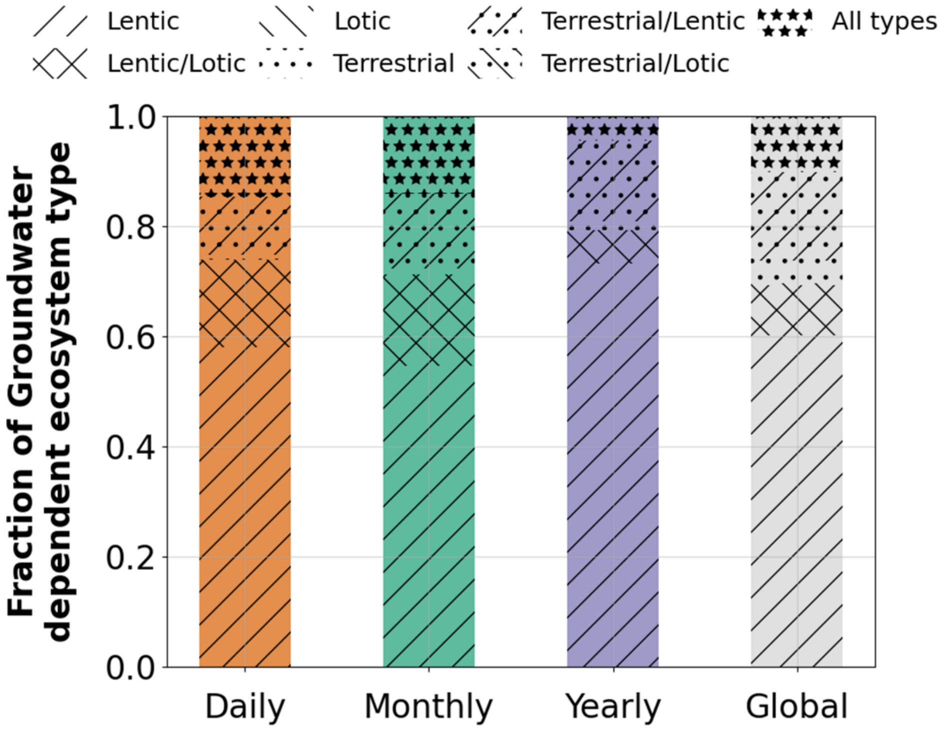

Fraction of groundwater dependent ecosystem type in GROW and globally

	Daily	Monthly	Yearly	Global*
**Lentic**	0.58	0.55	0.73	0.6
**Lentic/Lotic**	0.16	0.17	0.06	0.09
**Lotic**	<0.01	<0.01	<0.01	<0.01
**Terrestrial**	0.01	0.01	0.02	0.04
**Terrestrial/ Lentic**	0.11	0.13	0.15	0.16
**Terrestrial/ Lotic**	<0.01	<0.01	<0.01	<0.01
**All types**	0.15	0.14	0.04	0.1

Figure S.2-17: Distribution of groundwater dependent ecosystems in the GROW dataset compared with the global distribution. *This stacked bar chart is showing the distribution of the groundwater dependent ecosystems derived from Huggins et al.*[18] *in GROW classified by temporal resolution of the time series in comparison to the global fractions of the variable. *The global distributions are derived from all pixel values in the respective raster data with area-weighting to correct for the area distortion of the WGS 84 coordinate system.*

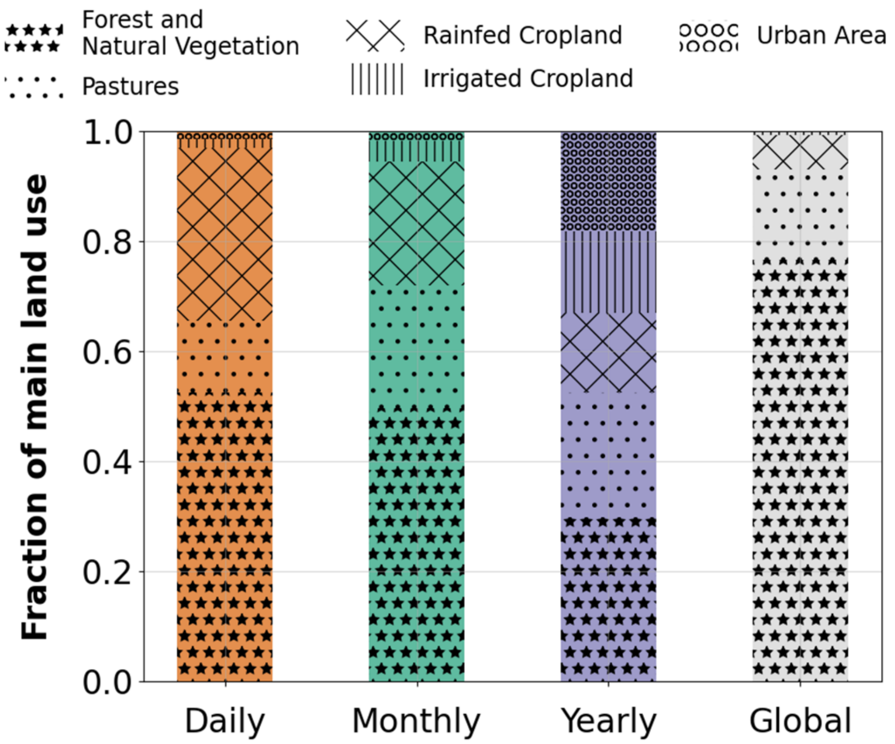

Fraction of main land use in GROW and globally

	Daily	Monthly	Yearly	Global*
**Forest and Natural Vegetation**	0.53	0.5	0.3	0.77
**Pastures**	0.13	0.22	0.23	0.16
**Rainfed Cropland**	0.31	0.22	0.14	0.06
**Irrigated Cropland**	0.02	0.04	0.15	< 0.01
**Urban Area**	0.02	0.02	0.18	< 0.01

Figure S.2-18: Distribution of main land use in the GROW dataset compared with the global distribution. *This stacked bar chart is showing the distribution of the main land use derived from Volkholz & Ostberg[19] in GROW classified by temporal resolution of the time series in comparison to the global fractions of the variable. *The global distributions are derived from all pixel values in the respective raster data with area-weighting to correct for the area distortion of the WGS 84 coordinate system.*

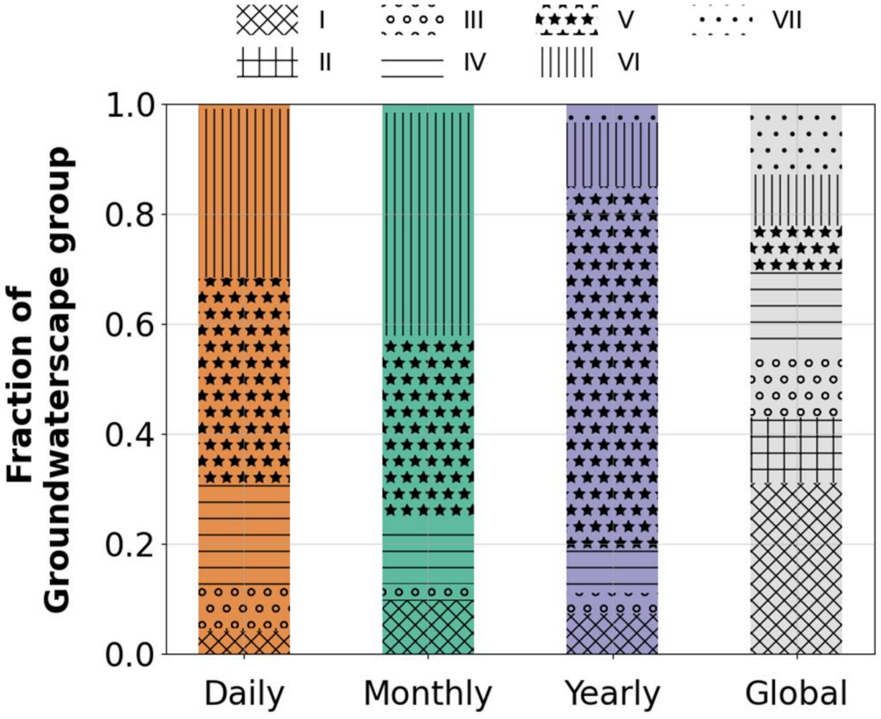

Fraction of groundwaterscape group in GROW and globally

	Daily	Monthly	Yearly	Global*
I	0.04	0.1	0.07	0.31
II	0.01	<0.01	<0.01	0.12
III	0.08	0.03	0.04	0.12
IV	0.18	0.12	0.08	0.15
V	0.38	0.34	0.66	0.08
VI	0.31	0.41	0.12	0.09
VII	0.01	0.02	0.03	0.13

Figure S.2-19: Distribution of Groundwaterscapes in the GROW dataset compared with the global distribution. *This stacked bar chart is showing the distribution of the Groundwaterscape groups derived from Huggins et al.*[28] *in GROW classified by temporal resolution of the time series in comparison to the global fractions of the variable. The different main groups are* ***I****-Arid and desert regions with minimal functions,* ***II****- Underserved populations and ineffective national governance,* ***III****- Earth system functions in non-agricultural regions,* ***IV****- Moderate GDEs and/or Earth system functions in nations with effective governance,* ***V****- Agricultural regions with high groundwater dependence,* ***VI****- Agricultural regions with low dependence on groundwater,* ***VII****- Extensive GDEs in non-agricultural regions. *The global distribution is derived from all pixel values in the respective raster data with area-weighting to correct for the area distortion of the WGS 84 coordinate system.*

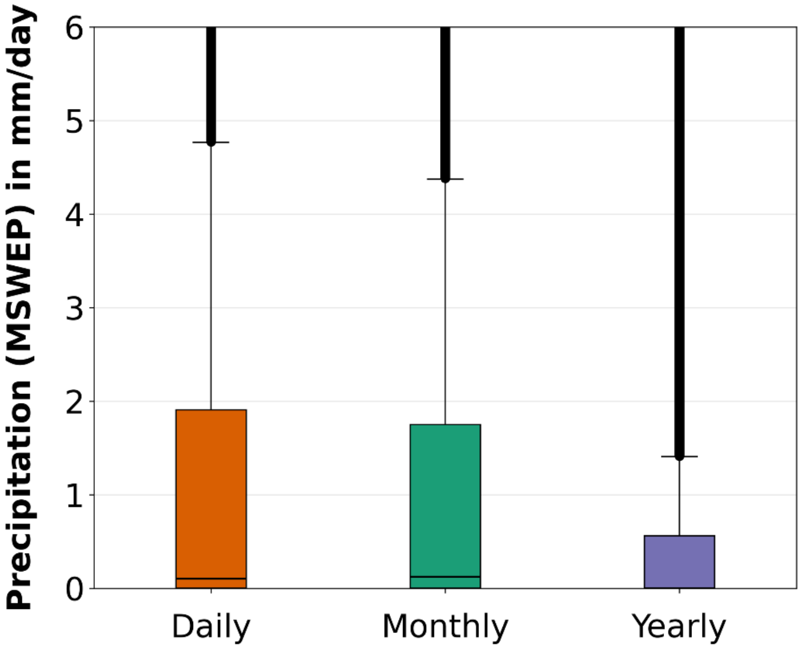

Descriptive statistics of daily precipitation (MSWEP) in mm/day in GROW and globally

	Daily	Monthly	Yearly	Global
**min**	0	0	0	0
**median**	0.1	0.13	0	0.38*
**max**	649.8	835.9	1642.6	1435.3

Figure S.2-20: Distribution of daily precipitation from MSWEP in the GROW dataset compared with the global distribution. *This overview is showing a boxplot of the daily precipitation derived from MSWEP V2*[21] *in GROW classified by temporal resolution of the time series. The GROW and global data was aggregated to a daily resolution to generate the figures. Therefore, the data is displayed in a daily resolution. Box plots show the median of a distribution as a black line inside the box. The upper and lower edges of the box are determined by the 25th and 75th percentiles (interquartile range). Whiskers indicate the farthest data point within 1.5 times the interquartile range. Outliers outside this range are displayed as dots. For readability, not all outliers are shown; thus, the tables below show the minimum, median, and maximum value of the precipitation in GROW for every temporal resolution in comparison to the global statistics.*The median of the global distribution is derived from all pixel values in the respective raster data with area-weighting to correct for the area distortion of the WGS 84 coordinate system. To enable computational feasibility only data from the year 2014, aggregated to a daily resolution (originally 3-hourly resolution), was used to calculate the global median.*

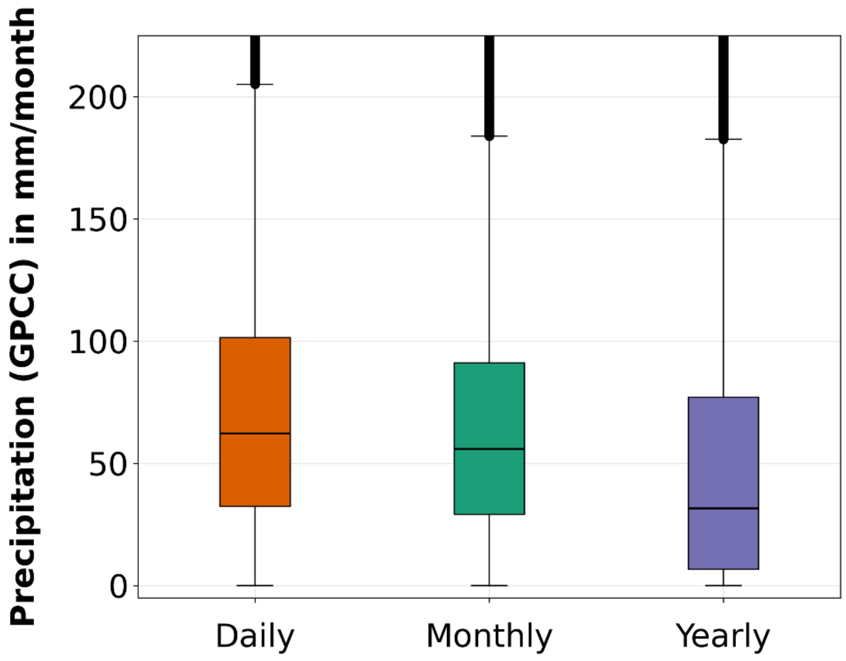

Descriptive statistics of monthly precipitation (GPCC) in mm/month in GROW and globally

	Daily	Monthly	Yearly	Global
**min**	0	0	0	0
**median**	62.3	55.9	31.6	32.5*
**max**	1826.3	2271.8	3357.8	5143.9

Figure S.2-21: Distribution of monthly precipitation from GPCC in the GROW dataset compared with the global distribution. *This overview is showing a boxplot of the monthly precipitation derived from GPCC[22] in GROW classified by temporal resolution of the time series. The GROW data before aggregation to the temporal resolution of the groundwater time series was used to generate the figures. Therefore, the data is displayed in the original resolution of the data source. Box plots show the median of a distribution as a black line inside the box. The upper and lower edges of the box are determined by the 25th and 75th percentiles (interquartile range). Whiskers indicate the farthest data point within 1.5 times the interquartile range. Outliers outside this range are displayed as dots. For readability, not all outliers are shown; thus, the tables below show the minimum, median, and maximum value of the precipitation in GROW for every temporal resolution in comparison to the global statistics. *The median of the global distribution is derived from all pixel values in the respective raster data with area-weighting to correct for the area distortion of the WGS 84 coordinate system.*

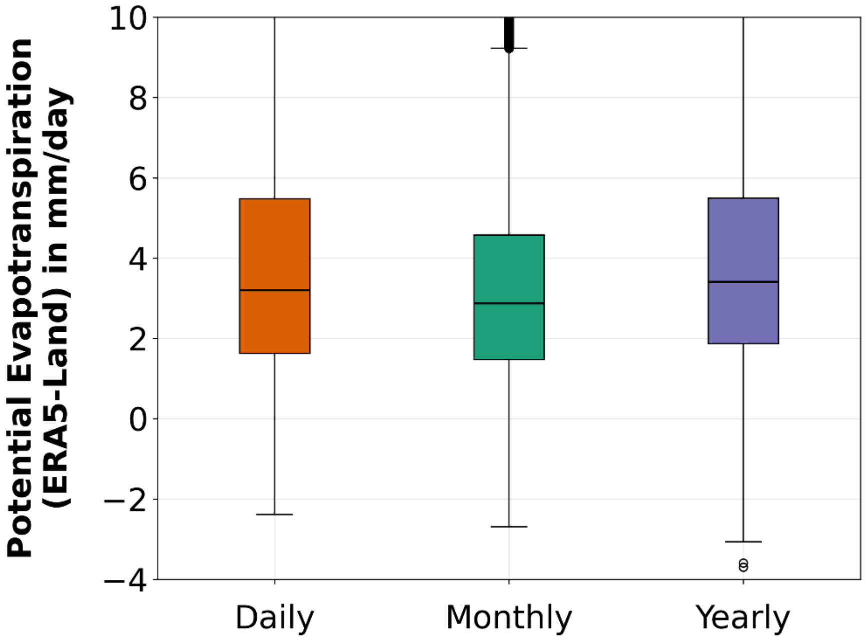

Descriptive statistics of daily potential evapotranspiration (ERA5-Land) in mm/day in GROW and globally

	Daily	Monthly	Yearly	Global
**min**	-4.30	-2.69	-5.98	-17.99
**median**	3.2	2.87	3.41	3.57*
**max**	73.91	74.65	98.1	88.4

Figure S.2-22: Distribution of daily potential evapotranspiration from ERA5-Land in the GROW dataset compared with the global distribution. *This overview is showing a boxplot of the daily potential evapotranspiration derived from ERA5-Land[16,17] in GROW classified by temporal resolution of the time series. The GROW data before aggregation to the temporal resolution of the groundwater time series was used to generate the figures. Therefore, the data is displayed in the original resolution of the data source. Box plots show the median of a distribution as a black line inside the box. The upper and lower edges of the box are determined by the 25th and 75th percentiles (interquartile range). Whiskers indicate the farthest data point within 1.5 times the interquartile range. Outliers outside this range are displayed as dots. For readability, not all outliers are shown; thus, the tables below show the minimum, median, and maximum value of the potential evapotranspiration in GROW for every temporal resolution in comparison to the global statistics.*The median of the global distribution is derived from all pixel values in the respective raster data with area-weighting to correct for the area distortion of the WGS 84 coordinate system. To enable computational feasibility only data from the year 2014 was used to calculate the global median.*

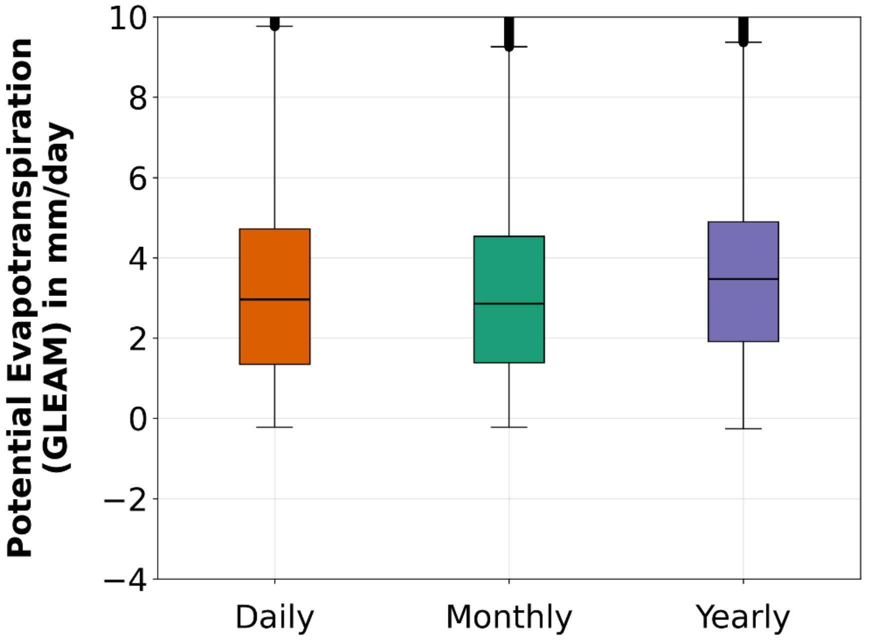

Descriptive statistics of daily potential evapotranspiration (GLEAM) in mm/day in GROW and globally

	Daily	Monthly	Yearly	Global
**min**	-0.22	-0.22	-0.26	-2
**median**	2.96	2.86	3.48	3.01*
**max**	34.95	97.1	96.62	29.28

Figure S.2-23: Distribution of daily potential evapotranspiration from GLEAM in the GROW dataset compared with the global distribution. *This overview is showing a boxplot of the potential evapotranspiration derived from GLEAM4[23] in GROW classified by temporal resolution of the time series. The GROW data before aggregation to the temporal resolution of the groundwater time series was used to generate the figures. Therefore, the data is displayed in the original resolution of the data source. Box plots show the median of a distribution as a black line inside the box. The upper and lower edges of the box are determined by the 25th and 75th percentiles (interquartile range). Whiskers indicate the farthest data point within 1.5 times the interquartile range. Outliers outside this range are displayed as dots. For readability, not all outliers are shown; thus, the tables below show the minimum, median, and maximum value of the potential evapotranspiration in GROW for every temporal resolution in comparison to the global statistics.*The median of the global distribution is derived from all pixel values in the respective raster data with area-weighting to correct for the area distortion of the WGS 84 coordinate system. To enable computational feasibility only data from the year 2014 was used to calculate the global median.*

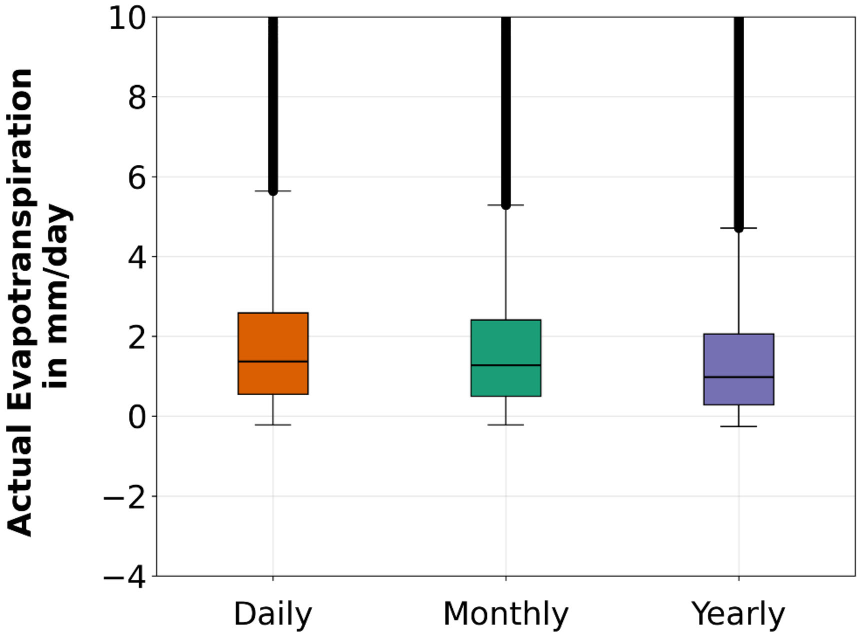

Descriptive statistics of daily actual evapotranspiration in mm/day in GROW and globally

	Daily	Monthly	Yearly	Global
min	-0.22	-0.22	-0.26	-2
median	1.37	1.28	0.98	0.68*
max	34.95	97.1	96.62	16.7

Figure S.2-24: Distribution of daily actual evapotranspiration from GLEAM in the GROW dataset compared with the global distribution. *This overview is showing a boxplot of the actual evapotranspiration derived from GLEAM4[23] in GROW classified by temporal resolution of the time series. The GROW data before aggregation to the temporal resolution of the groundwater time series was used to generate the figures. Therefore, the data is displayed in the original resolution of the data source. Box plots show the median of a distribution as a black line inside the box. The upper and lower edges of the box are determined by the 25th and 75th percentiles (interquartile range). Whiskers indicate the farthest data point within 1.5 times the interquartile range. Outliers outside this range are displayed as dots. For readability, not all outliers are shown; thus, the tables below show the minimum, median, and maximum value of the actual evapotranspiration in GROW for every temporal resolution in comparison to the global statistics.*The median of the global distribution is derived from all pixel values in the respective raster data with area-weighting to correct for the area distortion of the WGS 84 coordinate system. To enable computational feasibility only data from the year 2014 was used to calculate the global median.*

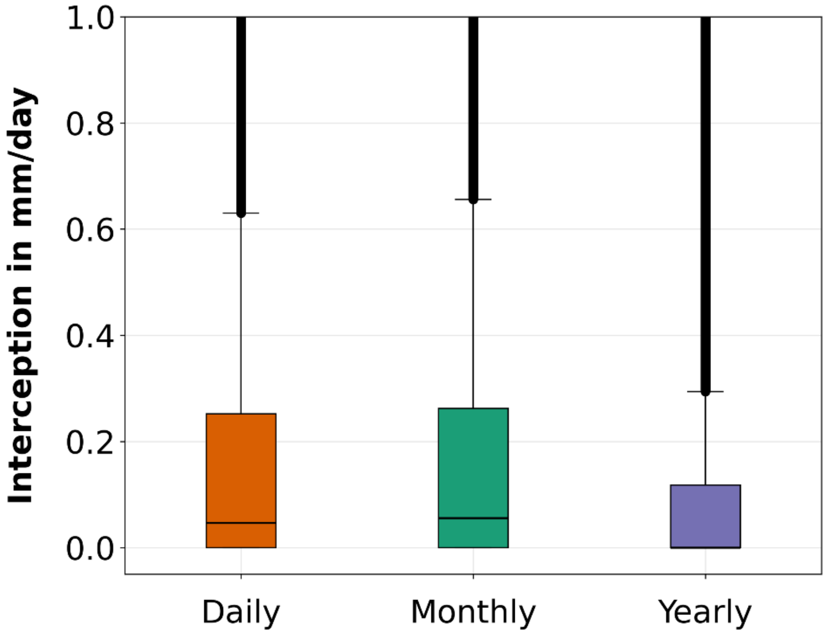

Descriptive statistics of daily interception in mm/day in GROW and globally

	Daily	Monthly	Yearly	Global
**min**	0	0	0	0
**median**	0.05	0.06	0	0*
**max**	34.17	97.06	96.46	42.79

Figure S.2-26: Distribution of daily interception in the GROW dataset compared with the global distribution. *This overview is showing a boxplot of interception derived from GLEAM4[23] in GROW classified by temporal resolution of the time series. The GROW data before aggregation to the temporal resolution of the groundwater time series was used to generate the figures. Therefore, the data is displayed in the original resolution of the data source. Box plots show the median of a distribution as a black line inside the box. The upper and lower edges of the box are determined by the 25th and 75th percentiles (interquartile range). Whiskers indicate the farthest data point within 1.5 times the interquartile range. Outliers outside this range are displayed as dots. For readability, not all outliers are shown; thus, the tables below show the minimum, median, and maximum value of the interception in GROW for every temporal resolution in comparison to the global statistics.*The median of the global distribution is derived from all pixel values in the respective raster data with area-weighting to correct for the area distortion of the WGS 84 coordinate system. To enable computational feasibility only data from the year 2014 was used to calculate the global median.*

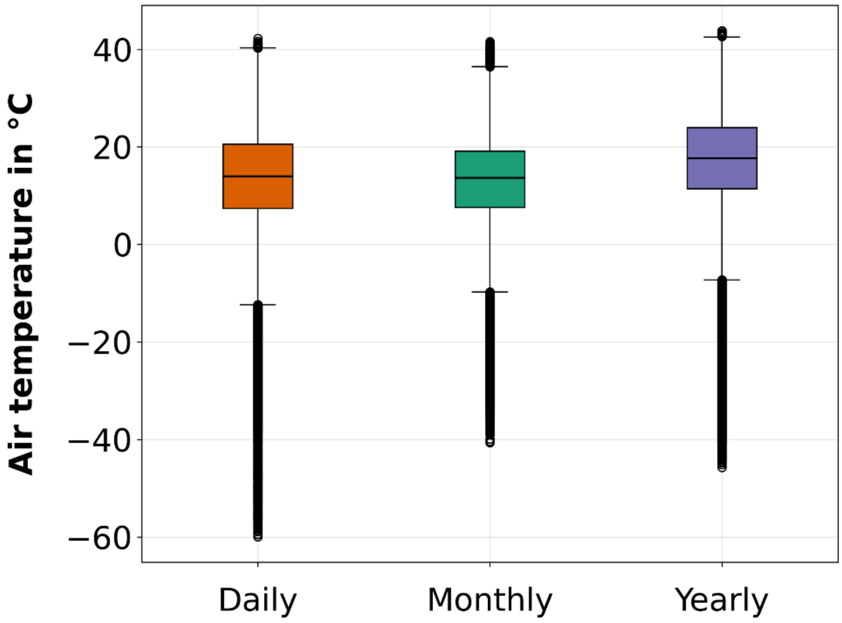

Descriptive statistics of daily average air temperature in GROW and globally

	Daily	Monthly	Yearly	Global
min	-59.9	-40.6	-45.7	-77.1
median	14	13.6	17.7	16.4*
max	42.3	41.6	43.8	43.7

Figure S.2-27: Distribution of daily average air temperature in the GROW dataset compared with the global distribution. *This overview is showing a boxplot of the air temperature derived from ERA5-Land[16,17] in GROW classified by temporal resolution of the time series. The GROW data before aggregation to the temporal resolution of the groundwater time series was used to generate the figures. Therefore, the data is displayed in the original resolution of the data source. Box plots show the median of a distribution as a black line inside the box. The upper and lower edges of the box are determined by the 25th and 75th percentiles (interquartile range). Whiskers indicate the farthest data point within 1.5 times the interquartile range. Outliers outside this range are displayed as dots. The tables below show the minimum, median, and maximum value of the air temperature in GROW for every temporal resolution in comparison to the global statistics.*The median of the global distribution is derived from all pixel values in the respective raster data with area-weighting to correct for the area distortion of the WGS 84 coordinate system. To enable computational feasibility only data from the year 2014 was used to calculate the global median.*

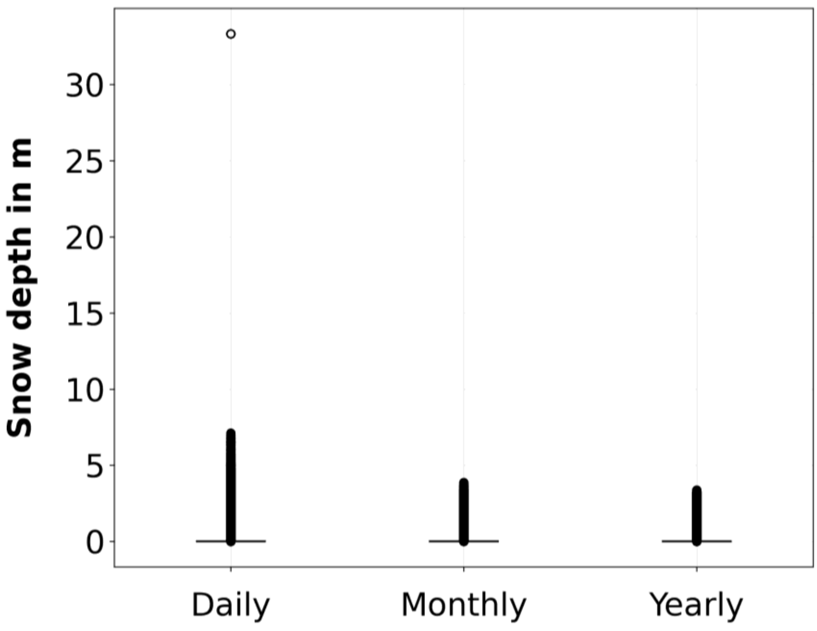

Descriptive statistics of daily average snow depth in m in GROW and globally

	Daily	Monthly	Yearly	Global
min	0	0	0	0
median	0	0	0	0*
max	33.33	3.87	3.39	33.33

Figure S.2-28: Distribution of daily average snow depth in the GROW dataset compared with the global distribution. *This overview is showing a boxplot of the snow depth derived from ERA5-Land[16,17] in GROW classified by temporal resolution of the time series. The GROW data before aggregation to the temporal resolution of the groundwater time series was used to generate the figures. Therefore, the data is displayed in the original resolution of the data source. Box plots show the median of a distribution as a black line inside the box. The upper and lower edges of the box are determined by the 25th and 75th percentiles (interquartile range). Whiskers indicate the farthest data point within 1.5 times the interquartile range. Outliers outside this range are displayed as dots. The tables below show the minimum, median, and maximum value of the snow depth in GROW for every temporal resolution in comparison to the global statistics.*The median of the global distribution is derived from all pixel values in the respective raster data with area-weighting to correct for the area distortion of the WGS 84 coordinate system. To enable computational feasibility only data from the year 2014 was used to calculate the global median.*

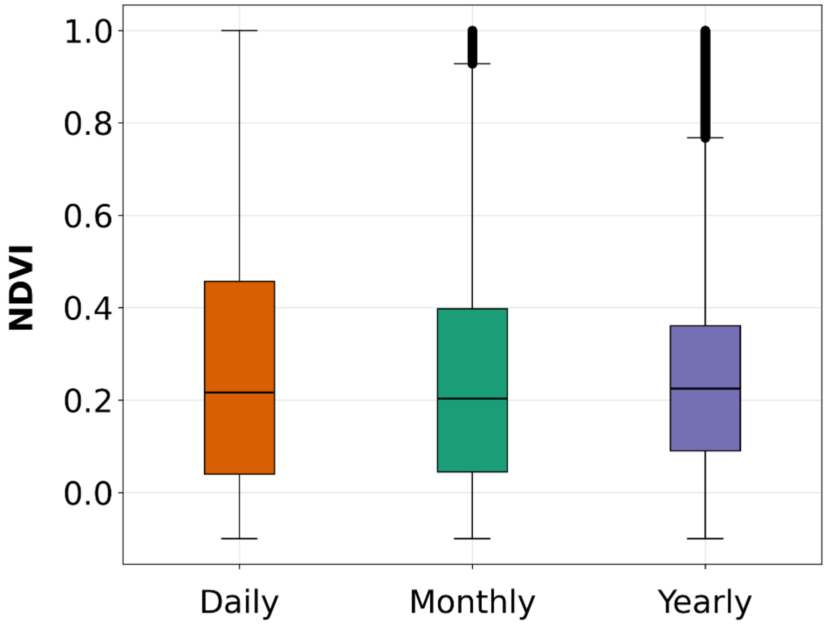

Descriptive statistics of daily average NDVI in GROW and globally

	Daily	Monthly	Yearly	Global
**min**	-0.1	-0.1	-0.1	-0.1
**median**	0.22	0.20	0.22	0.12*
**max**	1	1	1	1

Figure S.2-29: Distribution of daily average NDVI in the GROW dataset compared with the global distribution. *This overview is showing a boxplot of the NDVI derived from VIIRS NDVI[25] in GROW classified by temporal resolution of the time series. The GROW data before aggregation to the temporal resolution of the groundwater time series was used to generate the figures. Therefore, the data is displayed in the original resolution of the data source. Box plots show the median of a distribution as a black line inside the box. The upper and lower edges of the box are determined by the 25th and 75th percentiles (interquartile range). Whiskers indicate the farthest data point within 1.5 times the interquartile range. Outliers outside this range are displayed as dots. The tables below show the minimum, median, and maximum value of NDVI in GROW for every temporal resolution in comparison to the global statistics.*The median of the global distribution is derived from all pixel values in the respective raster data with area-weighting to correct for the area distortion of the WGS 84 coordinate system. To enable computational feasibility only data from the year 2014 was used to calculate the global median.*

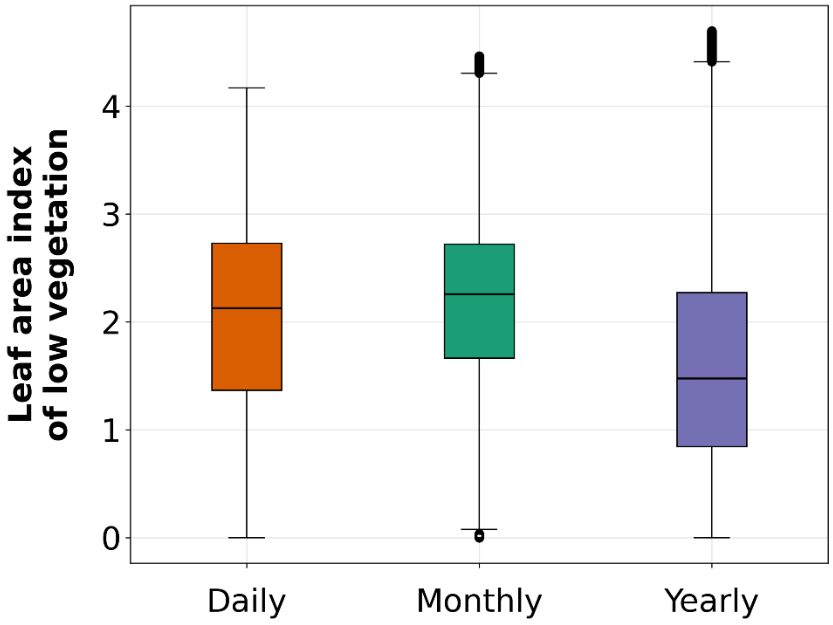

Descriptive statistics of daily average leaf area index of low vegetation in GROW and globally

	Daily	Monthly	Yearly	Global
**min**	0	0	0	0
**median**	2.13	2.26	1.48	0.82*
**max**	4.17	4.46	4.7	5.58

Figure S.2-30: Distribution of daily average leaf area index of low vegetation in the GROW dataset compared with the global distribution. *This overview is showing a boxplot of the leaf area index of low vegetation derived from ERA5-Land[16,17] in GROW classified by temporal resolution of the time series. The GROW data before aggregation to the temporal resolution of the groundwater time series was used to generate the figures. Therefore, the data is displayed in the original resolution of the data source. Box plots show the median of a distribution as a black line inside the box. The upper and lower edges of the box are determined by the 25th and 75th percentiles (interquartile range). Whiskers indicate the farthest data point within 1.5 times the interquartile range. Outliers outside this range are displayed as dots. The tables below show the minimum, median, and maximum value of the leaf area index in GROW for every temporal resolution in comparison to the global statistics.*The median of the global distribution is derived from all pixel values in the respective raster data with area-weighting to correct for the area distortion of the WGS 84 coordinate system. To enable computational feasibility only data from the year 2014 was used to calculate the global median.*

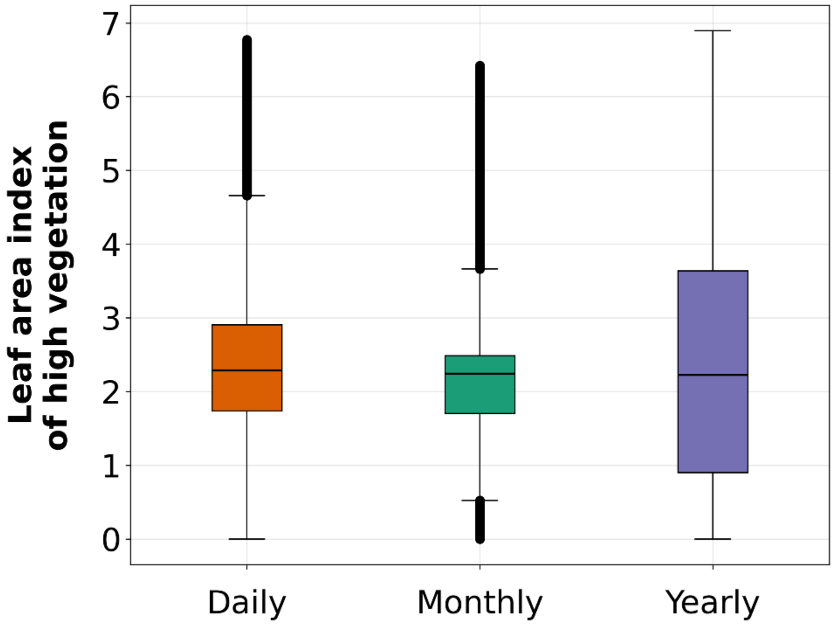

Descriptive statistics of daily average leaf area index of high vegetation in GROW and globally

	Daily	Monthly	Yearly	Global
**min**	0	0	0	0
**median**	2.29	2.24	2.23	1.26*
**max**	6.77	6.42	6.9	7.34

Figure S.2-31: Distribution of daily average leaf area index of high vegetation in the GROW dataset compared with the global distribution. *This overview is showing a boxplot of the leaf area index of high vegetation derived from ERA5-Land[16,17] in GROW classified by temporal resolution of the time series. The GROW data before aggregation to the temporal resolution of the groundwater time series was used to generate the figures. Therefore, the data is displayed in the original resolution of the data source. Box plots show the median of a distribution as a black line inside the box. The upper and lower edges of the box are determined by the 25th and 75th percentiles (interquartile range). Whiskers indicate the farthest data point within 1.5 times the interquartile range. Outliers outside this range are displayed as dots. The tables below show the minimum, median, and maximum value of the leaf area index in GROW for every temporal resolution in comparison to the global statistics.*The median of the global distribution is derived from all pixel values in the respective raster data with area-weighting to correct for the area distortion of the WGS 84 coordinate system. To enable computational feasibility only data from the year 2014 was used to calculate the global median.*

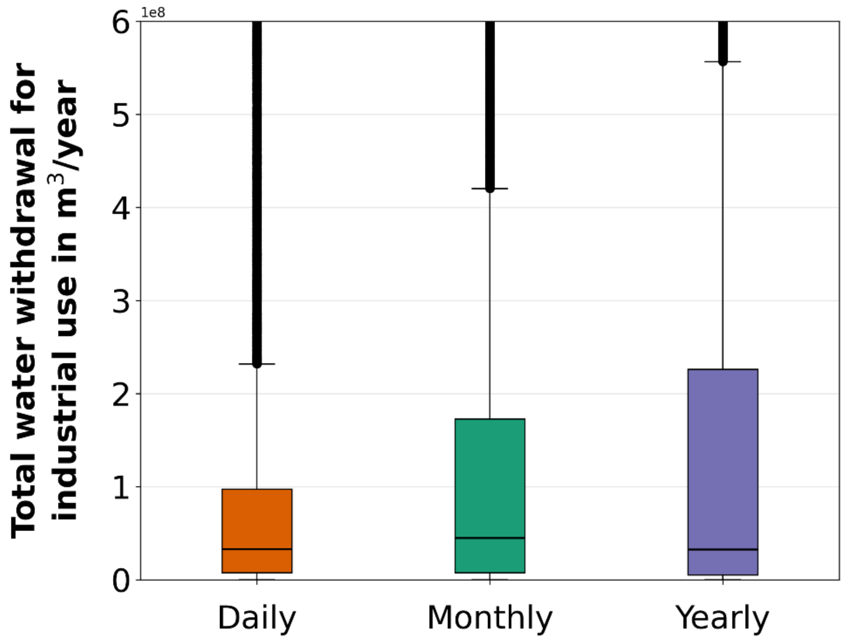

Descriptive statistics of annual total water withdrawal for industrial use in 10^9*m^3/year in GROW and globally

	Daily	Monthly	Yearly	Global
**min**	0	0	0	0
**median**	0.33	0.45	0.32	0*
**max**	5.3	3.3	5.3	6.36

Figure S.2-32: Distribution of annual total water withdrawal for industrial use in the GROW dataset compared with the global distribution. *This overview is showing a boxplot of the total water withdrawal for industrial use derived from Wada et al.[26] in GROW classified by temporal resolution of the time series. The GROW data before aggregation to the temporal resolution of the groundwater time series was used to generate the figures. Therefore, the data is displayed in the original resolution of the data source. Box plots show the median of a distribution as a black line inside the box. The upper and lower edges of the box are determined by the 25th and 75th percentiles (interquartile range). Whiskers indicate the farthest data point within 1.5 times the interquartile range. Outliers outside this range are displayed as dots. For readability, not all outliers are shown; thus, the tables below show the minimum, median, and maximum value of the total water withdrawal in GROW for every temporal resolution in comparison to the global statistics. *The median of the global distribution is derived from all pixel values in the respective raster data with area-weighting to correct for the area distortion of the WGS 84 coordinate system.*

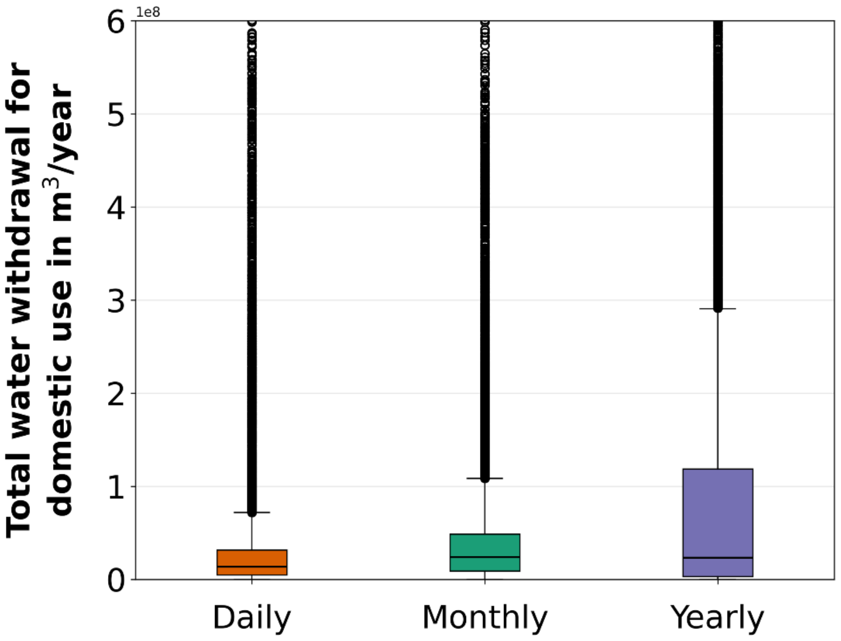

Descriptive statistics of annual total water withdrawal for domestic use in 10^9*m^3/year in GROW and globally

	Daily	Monthly	Yearly	Global
**min**	0	0	0	0
**median**	0.01	0.02	0.02	0*
**max**	2.16	1.11	2.16	2.28

Figure S.2-33: Distribution of annual total water withdrawal for domestic use in the GROW dataset compared with the global distribution. *This overview is showing a boxplot of the total water withdrawal for domestic use derived from Wada et al.*[26] *in GROW classified by temporal resolution of the time series. The GROW data before aggregation to the temporal resolution of the groundwater time series was used to generate the figures. Therefore, the data is displayed in the original resolution of the data source. Box plots show the median of a distribution as a black line inside the box. The upper and lower edges of the box are determined by the 25th and 75th percentiles (interquartile range). Whiskers indicate the farthest data point within 1.5 times the interquartile range. Outliers outside this range are displayed as dots. For readability, not all outliers are shown; thus, the tables below show the minimum, median, and maximum value of the total water withdrawal in GROW for every temporal resolution in comparison to the global statistics. *The median of the global distribution is derived from all pixel values in the respective raster data with area-weighting to correct for the area distortion of the WGS 84 coordinate system.*

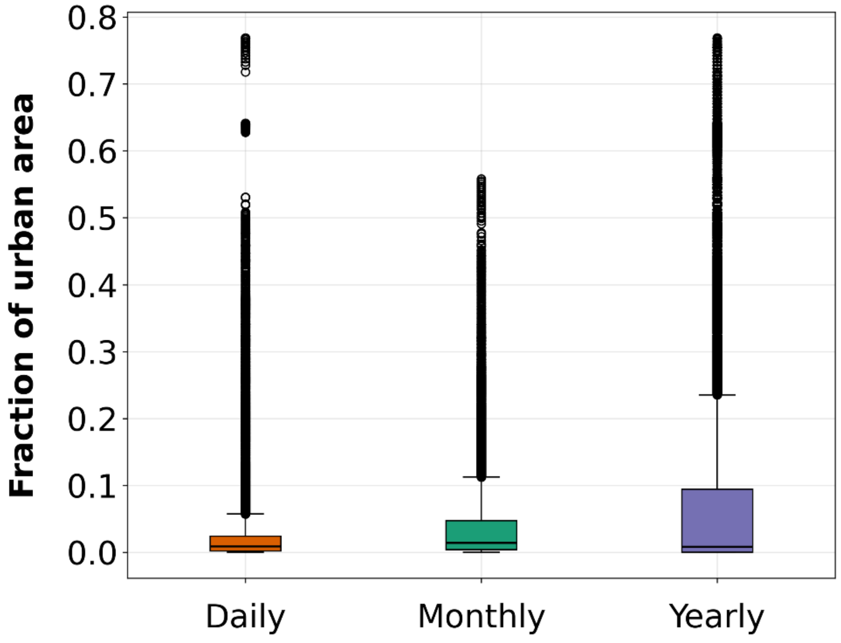

Descriptive statistics of annual urban area fraction in GROW and globally

	Daily	Monthly	Yearly	Global
**min**	0	0	0	0
**median**	0.01	0.01	0.01	0*
**max**	0.77	0.56	0.77	0.77

Figure S.2-34: Distribution of annual urban area fraction in the GROW dataset compared with the global distribution. *This overview is showing a boxplot of the fraction of urban area in the 0.5° raster pixel in which the GROW wells are located derived from Volkholz & Ostberg[19] and classified by temporal resolution of the time series. The GROW data before aggregation to the temporal resolution of the groundwater time series was used to generate the figures. Therefore, the data is displayed in the original resolution of the data source. Box plots show the median of a distribution as a black line inside the box. The upper and lower edges of the box are determined by the 25th and 75th percentiles (interquartile range). Whiskers indicate the farthest data point within 1.5 times the interquartile range. Outliers outside this range are displayed as dots. The tables below show the minimum, median, and maximum value of the urban area fraction in GROW for every temporal resolution in comparison to the global statistics.*The median of the global distribution is derived from all pixel values in the respective raster data with area-weighting to correct for the area distortion of the WGS 84 coordinate system.*

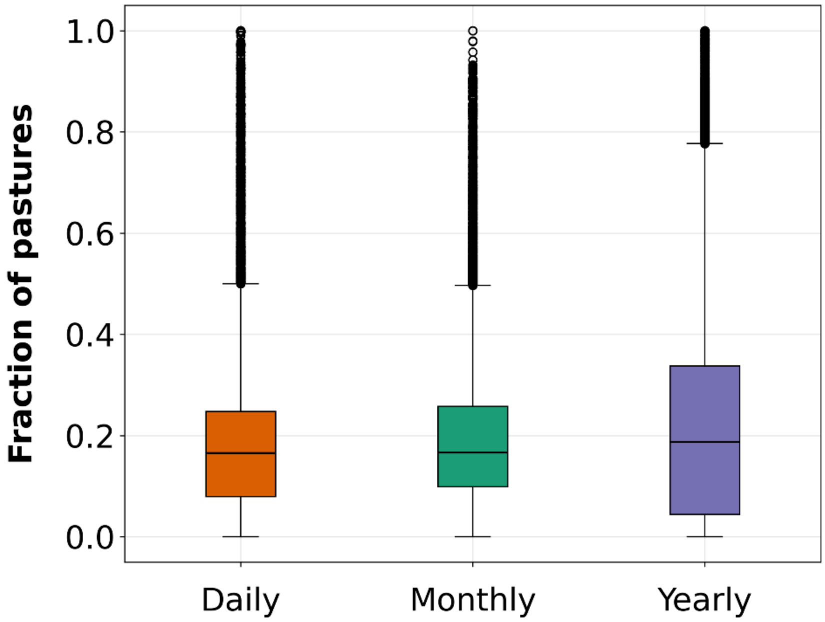

Descriptive statistics of annual pastures fraction in GROW and globally

	Daily	Monthly	Yearly	Global
**min**	0	0	0	0
**median**	0.17	0.17	0.19	0.05*
**max**	1	1	1	1

Figure S.2-35: Distribution of annual pastures fraction in the GROW dataset compared with the global distribution. *This overview is showing a boxplot of the fraction of pastures in the 0.5° raster pixel in which the GROW wells are located derived from Volkholz & Ostberg*[19] *and classified by temporal resolution of the time series. The GROW data before aggregation to the temporal resolution of the groundwater time series was used to generate the figures. Therefore, the data is displayed in the original resolution of the data source. Box plots show the median of a distribution as a black line inside the box. The upper and lower edges of the box are determined by the 25th and 75th percentiles (interquartile range). Whiskers indicate the farthest data point within 1.5 times the interquartile range. Outliers outside this range are displayed as dots. The tables below show the minimum, median, and maximum value of the pastures fraction in GROW for every temporal resolution in comparison to the global statistics.*The median of the global distribution is derived from all pixel values in the respective raster data with area-weighting to correct for the area distortion of the WGS 84 coordinate system.*

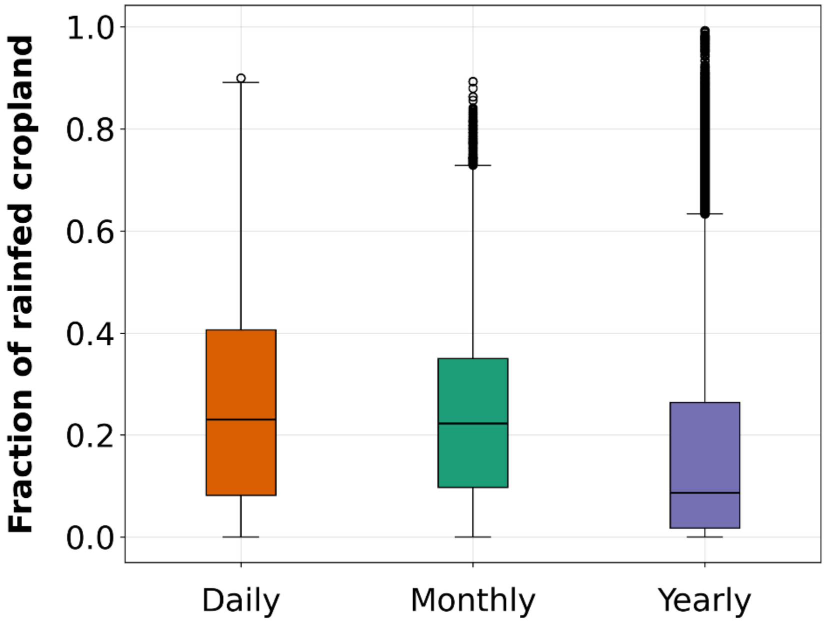

Descriptive statistics of annual rainfed cropland fraction in GROW and globally

	Daily	Monthly	Yearly	Global
**min**	0	0	0	0
**median**	0.23	0.22	0.09	<0.01*
**max**	0.9	0.89	0.99	1

Figure S.2-36: Distribution of annual rainfed cropland fraction in the GROW dataset compared with the global distribution. *This overview is showing a boxplot of the fraction of rainfed cropland in the 0.5° raster pixel in which the GROW wells are located derived from Volkholz & Ostberg[19] and classified by temporal resolution of the time series. The GROW data before aggregation to the temporal resolution of the groundwater time series was used to generate the figures. Therefore, the data is displayed in the original resolution of the data source. Box plots show the median of a distribution as a black line inside the box. The upper and lower edges of the box are determined by the 25th and 75th percentiles (interquartile range). Whiskers indicate the farthest data point within 1.5 times the interquartile range. Outliers outside this range are displayed as dots. The tables below show the minimum, median, and maximum value of the rainfed cropland fraction in GROW for every temporal resolution in comparison to the global statistics.*The median of the global distribution is derived from all pixel values in the respective raster data with area-weighting to correct for the area distortion of the WGS 84 coordinate system.*

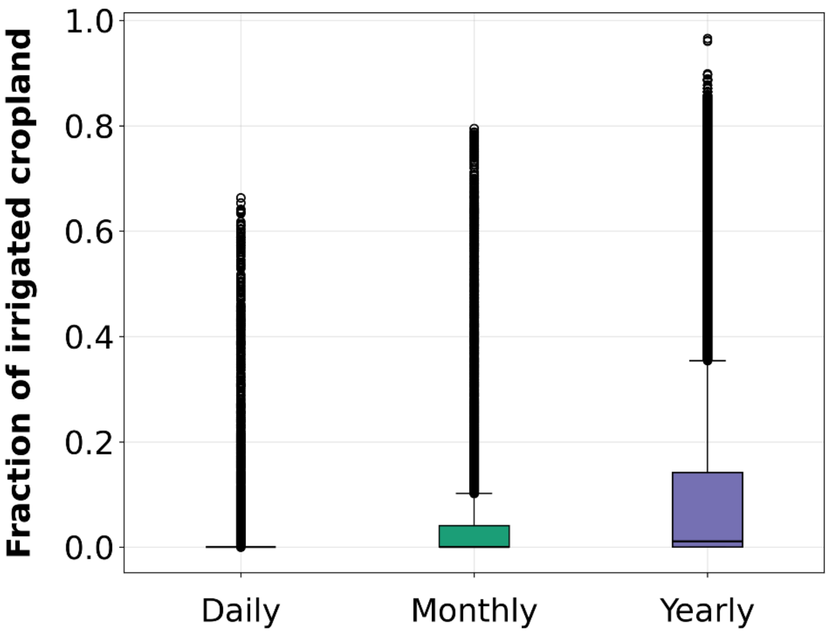

Descriptive statistics of annual irrigated cropland fraction in GROW and globally

	Daily	Monthly	Yearly	Global
**min**	0	0	0	0
**median**	0	0	0.01	0*
**max**	0.66	0.79	0.97	0.99

Figure S.2-37: Distribution of annual irrigated cropland fraction in the GROW dataset compared with the global distribution. *This overview is showing a boxplot of the fraction of irrigated cropland in the 0.5° raster pixel in which the GROW wells are located derived from Volkholz & Ostberg[19] and classified by temporal resolution of the time series. The GROW data before aggregation to the temporal resolution of the groundwater time series was used to generate the figures. Therefore, the data is displayed in the original resolution of the data source. Box plots show the median of a distribution as a black line inside the box. The upper and lower edges of the box are determined by the 25th and 75th percentiles (interquartile range). Whiskers indicate the farthest data point within 1.5 times the interquartile range. Outliers outside this range are displayed as dots. The tables below show the minimum, median, and maximum value of the irrigated cropland fraction in GROW for every temporal resolution in comparison to the global statistics.*The median of the global distribution is derived from all pixel values in the respective raster data with area-weighting to correct for the area distortion of the WGS 84 coordinate system.*

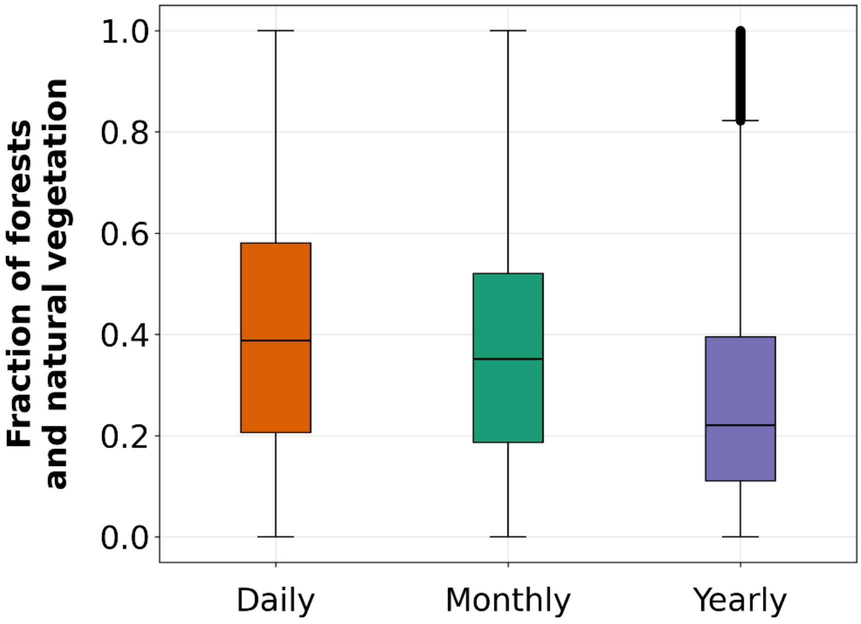

Descriptive statistics of annual forests and natural vegetation fraction in GROW and globally

	Daily	Monthly	Yearly	Global
**min**	0	0	0	0
**median**	0.39	0.35	0.22	0.67*
**max**	1	1	1	1

Figure S.2-38: Distribution of annual fraction of forests and natural vegetation in the GROW dataset compared with the global distribution. *This overview is showing a boxplot of the fraction of forests and natural vegetation in the 0.5° raster pixel in which the GROW wells are located derived from Volkholz & Ostberg[19] and classified by temporal resolution of the time series. The GROW data before aggregation to the temporal resolution of the groundwater time series was used to generate the figures. Therefore, the data is displayed in the original resolution of the data source. Box plots show the median of a distribution as a black line inside the box. The upper and lower edges of the box are determined by the 25th and 75th percentiles (interquartile range). Whiskers indicate the farthest data point within 1.5 times the interquartile range. Outliers outside this range are displayed as dots. The tables below show the minimum, median, and maximum value of the fraction of forests and natural vegetation in GROW for every temporal resolution in comparison to the global statistics.*The median of the global distribution is derived from all pixel values in the respective raster data with area-weighting to correct for the area distortion of the WGS 84 coordinate system.*

REFERENCES

1. Ester, M., Kriegel, H.-P., Sander, J. & Xu, X. A Density-Based Algorithm for Discovering Clusters in Large Spatial Databases with Noise. *Proc Second Int Conf Knowl Discov Data Min. KDD-96* 226–231 (1996).
2. Santini, M., Taramelli, A. & Sorichetta, A. ASPHAA: A GIS-Based Algorithm to Calculate Cell Area on a Latitude-Longitude (Geographic) Regular Grid: ASPHAA: A GIS-Based Algorithm. *Trans. GIS* 14, 351–377 (2010).
3. Karger, D. N. *et al.* Climatologies at high resolution for the earth's land surface areas CHELSA V2.1 (current). EnviDat https://doi.org/10.16904/ENVIDAT.228.V2.1 (2021).
4. Meybeck, M., Kummu, M. & Dürr, H. H. Global hydrobelts and hydroregions. PANGAEA https://doi.org/10.1594/PANGAEA.806957 (2013).
5. Yamazaki, D. *et al.* A high-accuracy map of global terrain elevations. *Geophys. Res. Lett.* 44, 5844–5853 (2017).
6. Amatulli, G. Geomorpho90m - Global high-resolution geomorphometry layers. PANGAEA https://doi.org/10.1594/PANGAEA.899135 (2019).
7. Hartmann, J. & Moosdorf, N. Global Lithological Map Database v1.0 (gridded to 0.5° spatial resolution). PANGAEA https://doi.org/10.1594/PANGAEA.788537 (2012).
8. Chen, Z. *et al.* World Karst Aquifer Map (WHYMAP WOKAM). BGR , IAH, KIT, UNESCO https://doi.org/10.25928/B2.21_SFKQ-R406 (2017).
9. Hartmann, J. & Moosdorf, N. The new global lithological map database GLiM: A representation of rock properties at the Earth surface. *Geochem. Geophys. Geosystems* 13, 2012GC004370 (2012).
10. Huscroft, J., Gleeson, T., Hartmann, J. & Börker, J. Compiling and mapping global permeability of the unconsolidated and consolidated Earth: GLobal HYdrogeology MaPS 2.0 (GLHYMPS 2.0). Borealis https://doi.org/10.5683/SP2/TTJNIU (2018).
11. Gleeson, T. GLobal HYdrogeology MaPS (GLHYMPS) of permeability and porosity. Borealis https://doi.org/10.5683/SP2/DLGXYO (2018).
12. Simons, G., Koster, R. & Droogers, P. HiHydroSoil v2.0 - A high resolution soil map of global hydraulic properties. https://www.futurewater.nl/wp-content/uploads/2020/10/HiHydroSoil-v2.0-High-Resolution-Soil-Maps-of-Global-Hydraulic-Properties.pdf (2020).
13. Cuthbert, M. O. *et al.* Observed controls on resilience of groundwater to climate variability in sub-Saharan Africa. *Nature* 572, 230–234 (2019).
14. Lehner, B. & Grill, G. Global river hydrography and network routing: baseline data and new approaches to study the world's large river systems. *Hydrol. Process.* 27, 2171–2186 (2013).
15. Linke, S. *et al.* Global hydro-environmental sub-basin and river reach characteristics at high spatial resolution. *Sci. Data* 6, 283 (2019).
16. Muñoz Sabater, J. *et al.* ERA5-land post-processed daily-statistics from 1950 to present. Copernicus Climate Change Service (C3S) Climate Data Store (CDS). https://doi.org/10.24381/cds.e9c9c792 (2024).
17. Copernicus Climate Change Service. ERA5-Land post-processed daily statistics from 1950 to present. ECMWF https://doi.org/10.24381/CDS.E9C9C792 (2024).
18. Huggins, X. *et al.* Data from: Overlooked risks and opportunities in groundwatersheds of the world's protected areas. Borealis https://doi.org/10.5683/SP3/P3OU3A (2023).
19. Volkholz, J. & Ostberg, S. ISIMIP3a landuse input data. ISIMIP Repository https://doi.org/10.48364/ISIMIP.571261.3 (2024).
20. Huggins, X., Gleeson, T., Villholth, K. G., Rocha, J. C. & Famiglietti, J. S. Data and code from: Groundwaterscapes: A global classification and mapping of groundwater's large-scale socioeconomic, ecological, and Earth system functions. Borealis https://doi.org/10.5683/SP3/MFYCWV (2024).

21. Beck, H. E. *et al.* MSWEP V2 Global 3-Hourly 0.1° Precipitation: Methodology and Quantitative Assessment. *Bull. Am. Meteorol. Soc.* 100, 473–500 (2019).
22. Schneider, U., Hänsel, S., Finger, P., Rustemeier, E. & Ziese, M. GPCC Full Data Monthly Version 2022 at 0.25°: Monthly Land-Surface Precipitation from Rain-Gauges built on GTS-based and Historic Data: Globally Gridded Monthly Totals. Global Precipitation Climatology Centre (GPCC) at Deutscher Wetterdienst https://doi.org/10.5676/DWD_GPCC/FD_M_V2022_025 (2022).
23. Miralles, D. G. *et al.* GLEAM4: global land evaporation and soil moisture dataset at 0.1° resolution from 1980 to near present. *Sci. Data* 12, 416 (2025).
24. Vermote, E. & NOAA CDR Program. NOAA Climate Data Record (CDR) of AVHRR Normalized Difference Vegetation Index (NDVI), Version 5. NOAA National Centers for Environmental Information https://doi.org/10.7289/V5ZG6QH9 (2018).
25. Vermote, E. & NOAA CDR Program. NOAA Climate Data Record (CDR) of VIIRS Normalized Difference Vegetation Index (NDVI), Version 1. NOAA National Centers for Environmental Information https://doi.org/10.25921/GAKH-ST76 (2022).
26. Wada, Y. *et al.* ISIMIP3a water abstraction input data. ISIMIP Repository https://doi.org/10.48364/ISIMIP.228996 (2022).
27. Cuthbert, M. O. *et al.* Global patterns and dynamics of climate–groundwater interactions. *Nat. Clim. Change* 9, 137–141 (2019).
28. Huggins, X., Gleeson, T., Villholth, K. G., Rocha, J. C. & Famiglietti, J. S. Groundwaterscapes: A Global Classification and Mapping of Groundwater's Large-Scale Socioeconomic, Ecological, and Earth System Functions. *Water Resour. Res.* **60**, e2023WR036287 (2024).
